# Quantitative MRI Characterization of Fluid-Related Phenotypes in Pediatric Epilepsy: Deep Learning-Assisted Assessment of Enlarged Perivascular Space Burden, Regional Distribution, and Clinical Associations

**DOI:** 10.3390/diagnostics16203304

**Published:** 2026-10-11

**Authors:** Adil Aytaç, Hilal Aydın

**Affiliations:** 1 Department of Radiology, Faculty of Medicine, Health Practice and Research Hospital, Balikesir University, 10145 Balıkesir, Turkey; 2 Department of Pediatric Neurology, Faculty of Medicine, Health Practice and Research Hospital, Balikesir University, 10145 Balıkesir, Turkey

**Keywords:** epilepsy, glymphatic system, cerebrospinal fluid, magnetic resonance imaging

## Abstract

**Objective:** To quantitatively characterize EPVS burden and regional distribution, together with CP and ventricular CSF structural imaging phenotypes, in pediatric epilepsy and to explore within-epilepsy clinical associations of EPVS measures differing from controls. **Methods:** This retrospective cross-sectional study included 123 children with epilepsy and 108 controls aged 10–17 years. Enlarged perivascular space (EPVS), choroid plexus (CP), and ventricular cerebrospinal fluid (CSF) structural imaging phenotypes were quantitatively assessed using SHIVA-PVS, FreeSurfer, and 3D Slicer with expert visual quality control. Primary between-group comparisons used general linear models adjusted for age and sex, with estimated total intracranial volume additionally included for absolute volumetric measures, and multiple comparisons were controlled using the false discovery rate (FDR). Exploratory within-epilepsy clinical-association analyses were subsequently performed for EPVS measures showing FDR-significant between-group differences. **Results:** Global EPVS burden was higher in the epilepsy group than in controls (*q* < 0.01), with the most pronounced regional differences in the basal ganglia (BG; *q* < 0.001). Relative distribution measures showed higher BG burden and lower centrum semiovale (CSO) burden and CSO/BG ratios in epilepsy (*q* < 0.01). No statistically significant between-group differences were observed in the CP or ventricular CSF phenotypes. In exploratory within-epilepsy analyses, longer disease duration and drug resistance were associated predominantly with BG-EPVS measures, whereas associations with antiseizure medication burden were limited. Bilateral epileptiform activity was also associated with higher BG-EPVS burden after FDR correction (*q* < 0.05). **Conclusions:** Primary between-group analyses identified greater EPVS burden in pediatric epilepsy, with the most pronounced regional differences involving the BG. Exploratory within-epilepsy analyses identified associations between selected EPVS measures and specific clinical characteristics. These cross-sectional structural imaging findings characterize a regional EPVS phenotype for further longitudinal and multimodal investigation but do not establish an underlying glymphatic mechanism.

## 1. Introduction

Epilepsy is one of the most common chronic neurological disorders of childhood [1]. The disorder represents a substantial health burden due to recurrent seizures and the cognitive, behavioral, and neurodevelopmental problems that may accompany them [1]. The pathophysiology of epilepsy has traditionally been explained in terms of neuronal hyperexcitability and abnormal synchronized electrical activity [1]. Consequently, research has focused predominantly on neuronal and synaptic mechanisms for many years [1]. However, emerging evidence suggests that this framework alone may not fully account for the pathophysiology of epilepsy [1,2]. There is growing recognition that processes such as astrocytic dysfunction, neuroinflammation, disruption of blood–brain barrier integrity, neurovascular unit dysfunction, and alterations in extracellular fluid homeostasis may be associated with the pathophysiological processes underlying epilepsy [1,2]. A common feature of these processes is their close relationship with biological mechanisms involved in maintaining the integrity of the brain microenvironment and tissue homeostasis [1,2]. Accordingly, epilepsy research is expanding beyond approaches centered primarily on neuronal excitability toward a more integrated evaluation of the mechanisms that regulate brain homeostasis [1,2]. Within this evolving biological paradigm, the glymphatic system is increasingly being investigated as one of the potential mechanisms associated with brain fluid homeostasis [3,4].

The glymphatic system is described as a perivascular fluid transport system that facilitates solute exchange between cerebrospinal fluid (CSF) and interstitial fluid and contributes to the maintenance of homeostasis within the brain microenvironment [5,6,7]. Within this system, CSF enters the brain parenchyma along periarterial spaces and interacts with interstitial fluid through aquaporin-4 water channels, which are highly enriched in astrocytic endfeet [5,6,7]. The glymphatic system also contributes to the clearance of metabolic waste products and other solutes through perivenous pathways and associated meningeal lymphatic drainage mechanisms [5,6,7]. Thus, the glymphatic system is implicated in metabolic clearance, ionic balance, extracellular fluid homeostasis, and the maintenance of the physiological integrity of the neuronal microenvironment [5,6]. Available experimental and clinical evidence suggests that glymphatic function may be influenced by several physiological and pathophysiological factors, including the sleep–wake cycle, vascular pulsatility, aquaporin-4 polarization, aging, and neuroinflammation [5,6,7]. Accordingly, the glymphatic system has emerged as an area of growing interest in investigations of the relationship between brain fluid dynamics and neurological disorders [6,7]. Accumulating evidence further suggests that glymphatic dysfunction may be associated with the development and progression of a range of neurological disorders [6,7].

Evidence of glymphatic system dysfunction in the current literature has been derived predominantly from studies of neurodegenerative disorders [8,9]. Findings suggestive of impaired glymphatic function have been reported in conditions such as Alzheimer disease, Parkinson disease, dementia with Lewy bodies, and traumatic brain injury [8,9,10]. In Alzheimer disease, impaired glymphatic transport has been associated with insufficient clearance of amyloid-β and tau proteins, whereas in Parkinson disease and dementia with Lewy bodies, impaired α-synuclein clearance and accompanying neuroinflammatory processes have been linked to glymphatic dysfunction [8,9]. Similarly, acute glymphatic dysfunction following traumatic brain injury has been shown to impair the clearance of metabolic waste products, potentially exacerbating secondary brain injury [10]. These associations are increasingly supported not only by experimental models but also by evidence from advanced imaging techniques and translational human studies [8,9,10]. Although glymphatic dysfunction has been studied predominantly in the context of neurodegenerative disorders, it is increasingly considered relevant to the pathophysiology of a broader range of neurological diseases [8,9,10]. Consequently, interest in investigating alterations in the glymphatic system has expanded beyond neurodegenerative disorders to other neurological conditions [8,9,10].

Several pathophysiological mechanisms described in epilepsy are thought to be associated with biological processes that may influence glymphatic system function [2,3,4,5,6,7]. In particular, recurrent seizure activity, astrocytic reactivity, impaired aquaporin-4 polarization, increased blood–brain barrier permeability, neuroinflammation, and alterations in CSF dynamics have been proposed to adversely affect perivascular fluid transport and interstitial solute clearance [2,3,4,5,6,7]. In this context, recent studies in adults with epilepsy have sought to indirectly assess glymphatic dysfunction using routine magnetic resonance imaging (MRI) findings and advanced imaging biomarkers [3,4,11,12,13,14,15,16,17,18]. Studies using diffusion tensor imaging analysis along the perivascular space (DTI-ALPS) have reported reduced DTI-ALPS indices or hemispheric asymmetry, particularly in patients with focal epilepsy and temporal lobe epilepsy [11,12,13,14,15]. In addition, regional alterations in enlarged perivascular space (EPVS) burden and increased choroid plexus (CP) volume have been proposed as imaging features potentially associated with glymphatic dysfunction [14,16,17,18]. However, most available studies and supporting evidence have been derived from adult populations, with substantial heterogeneity across studies in terms of patient populations, epilepsy subtypes, clinical variables, imaging biomarkers evaluated, and methodological approaches [3,4,11,12,13,14,15,16,17,18]. Taken together, the available findings suggest a potential association between epilepsy and biological alterations and imaging biomarkers related to glymphatic dysfunction; however, the nature and clinical significance of this relationship remain to be clarified through more comprehensive and standardized studies encompassing diverse epilepsy phenotypes [3,4].

Studies evaluating imaging biomarkers associated with glymphatic dysfunction in childhood epilepsy remain limited [3,4,19,20]. Nevertheless, pediatric populations may provide an opportunity to investigate the potential relationship between glymphatic dysfunction and epilepsy within a distinct biological context. During childhood, factors that may influence imaging findings related to glymphatic dysfunction, such as aging, neurodegeneration, and chronic cerebrovascular disease, are considerably less common than in adult populations. This may allow epilepsy-associated alterations related to glymphatic dysfunction to be evaluated in the presence of fewer potential confounding factors. However, it should also be recognized that imaging biomarkers potentially associated with glymphatic dysfunction may themselves be influenced by neurodevelopmental processes [21]. Therefore, the potential effects of age and sex on these imaging biomarkers should be carefully considered in studies of pediatric epilepsy.

No widely accepted and standardized imaging biomarker is currently available for the direct assessment of glymphatic system dysfunction in clinical practice. Consequently, various imaging approaches have been investigated to evaluate glymphatic-related fluid dynamics and their potential alterations [3,4,5,6,7]. Among these, the DTI-ALPS index has gained increasing use in recent years and is derived from water diffusion properties along directions corresponding to the expected orientation of perivascular spaces at specific anatomical levels [3,4,11,12,13,14,15,19,20,21,22]. Although its noninvasive nature and the possibility of retrospective application to datasets containing suitable diffusion tensor imaging have facilitated its widespread use, the DTI-ALPS index remains an indirect imaging biomarker that does not directly measure glymphatic fluid transport [22]. Moreover, its reliability may be influenced not only by acquisition parameters, such as imaging plane and diffusion protocol, but also by analysis-related factors, including image processing, anatomical alignment, and region-of-interest placement [22]. Reliable DTI-ALPS analysis therefore requires diffusion images of sufficient quality and consistent analytical approaches [22]. However, diffusion tensor imaging suitable for DTI-ALPS analysis may not be available in routine clinical datasets, and the technical characteristics of available images may vary across centers. These limitations highlight the need to investigate complementary structural and quantitative imaging biomarkers that can be derived from routine brain MRI for the assessment of alterations associated with glymphatic dysfunction. Accordingly, recent studies have increasingly explored whether various imaging biomarkers obtainable from routine brain MRI may indirectly reflect biological processes associated with glymphatic dysfunction [3,4,5,6,7,23].

Brain MRI-based approaches with the potential to capture alterations associated with glymphatic dysfunction include analyses of EPVS distribution and burden, CP morphometry, and volumetric measurements of ventricular CSF compartments. Among these, EPVS burden has been associated with perivascular fluid exchange and interstitial solute clearance; CP morphology with CSF production, blood–CSF barrier function, and neuroimmune regulation; and volumetric characteristics of ventricular CSF compartments with intracranial fluid distribution and neurofluid homeostasis [5,6,7,16,17,18,23,24]. However, because each of these approaches reflects a different physiological component of the glymphatic system, no single potential imaging biomarker would be expected to comprehensively represent glymphatic dysfunction. In contrast, the combined assessment of complementary potential imaging biomarkers may enable different aspects of glymphatic-related alterations to be examined within a shared biological framework [23,24,25,26]. Another important advantage of this approach is that these imaging biomarkers can be derived from routine brain MRI protocols, offering the potential for straightforward implementation in both retrospective research and routine clinical practice [23,24,25,26]. Furthermore, imaging alterations associated with glymphatic dysfunction have largely been evaluated in the existing literature using individual biomarkers or a single imaging approach [11,12,13,14,15,16,17,18,19,20]. In childhood epilepsy in particular, most studies have focused on diffusion-based methods such as DTI-ALPS, whereas studies jointly evaluating complementary potential imaging biomarkers obtainable from routine brain MRI remain limited [19,20,21,22]. However, neurofluid homeostasis is a multicomponent system that depends on the interplay between fluid dynamics across different anatomical compartments and the physiological processes that regulate them [5,6,7,23,26]. Therefore, the integrated assessment of complementary imaging biomarkers may provide a means of examining different components of neurofluid homeostasis within a unified biological framework.

The aim of this study was to comparatively evaluate potential imaging biomarkers derived from routine brain MRI that may be associated with glymphatic system dysfunction in children with epilepsy and controls. We also aimed to investigate the potential associations between imaging biomarkers indicative of glymphatic dysfunction and clinical variables related to pediatric epilepsy. We hypothesized that children with epilepsy would exhibit significant differences in potential imaging biomarkers of glymphatic dysfunction compared with controls and that imaging biomarkers showing significant between-group differences would be associated with specific epilepsy-related clinical variables.

## 2. Materials and Methods

### 2.1. Study Design and Ethical Approval

This single-center, retrospective, cross-sectional observational study was conducted at Balıkesir University Faculty of Medicine. The study protocol was approved by the Balıkesir University Faculty of Medicine Health Research Ethics Committee (Decision No. 2026/10-13; 21 May 2026). All study procedures were conducted in accordance with the principles of the Declaration of Helsinki, and the study was reported in accordance with the Strengthening the Reporting of Observational Studies in Epidemiology (STROBE) guidelines. Written informed consent was obtained from all participants or their legal guardians before inclusion in the study. The institutional Picture Archiving and Communication System (PACS) and relevant clinical records were retrospectively reviewed to identify pediatric patients who underwent brain MRI between 1 January 2018, and 31 March 2026.

### 2.2. Study Population

The study included 123 children aged 10–17 years with a diagnosis of epilepsy and 108 clinical controls with structurally normal brain MRI within the same age range. The diagnosis and classification of epilepsy were established according to the International League Against Epilepsy (ILAE) 2017 classification framework by a pediatric neurologist with 10 years of experience in pediatric neurology, based on an integrated assessment of clinical history, seizure characteristics, and electroencephalography (EEG) findings [27]. To minimize the potential confounding effects of neurological, developmental, systemic, and imaging-related factors unrelated to epilepsy on quantitative MRI analyses, inclusion and exclusion criteria for both the epilepsy and control groups were predefined [21,23,26]. The study population was restricted to children aged 10–17 years to reduce the potential confounding effects of rapid neurodevelopmental changes and developmental variability in CSF compartments during early childhood and to establish a more homogeneous population for EPVS, CP, and ventricular CSF analyses [21,28].

Inclusion Criteria for the Epilepsy Group:

- Age 10–17 years;
- Diagnosis of epilepsy according to the ILAE 2017 classification criteria, established by a pediatric neurologist with 10 years of experience based on an integrated assessment of clinical and EEG data [27];
- Availability of diagnostically adequate imaging acquired at the same institution and meeting the technical quality requirements of the study, including isotropic three-dimensional (3D) T1-weighted images and 3D T2-Fluid-Attenuated Inversion Recovery (T2-FLAIR) images suitable for volumetric analysis;
- No structural lesion on brain MRI that could account for the epilepsy;
- No structural, developmental, or acquired abnormalities on brain MRI that could affect EPVS, CP, or CSF analyses;
- Complete clinical and imaging data.

Inclusion Criteria for the Control Group:

- Age 10–17 years;
- Brain MRI performed for headache or dizziness in the absence of a documented neurological disorder accounting for these symptoms; the available clinical records contained no documented diagnosis of migraine, chronic tension-type headache, or a vestibular disorder among participants included in the control group;
- No diagnosis of epilepsy, history of seizures, or unexplained recurrent episodes of loss of consciousness;
- Normal findings on neurological examination;
- Structurally normal brain MRI findings;
- No history of neurological, neurodevelopmental, psychiatric, or chronic systemic disease;
- No significant structural abnormality on brain MRI that could affect EPVS, CP, or CSF analyses;
- Availability of diagnostically adequate imaging acquired at the same institution and meeting the technical quality requirements of the study, including isotropic 3D T1-weighted and T2-FLAIR images suitable for volumetric analysis;
- Complete clinical and imaging data.

Among the 108 included clinical controls, the indication for brain MRI was headache in 60 participants (55.6%) and dizziness in 48 (44.4%). These represented presenting complaints/referral indications rather than established final diagnoses.

All brain MRI examinations in the epilepsy and control groups were independently re-evaluated by a neuroradiologist with 9 years of experience to confirm eligibility according to the predefined inclusion criteria. As part of this imaging review, no participant included in the final epilepsy cohort demonstrated MRI evidence of cytotoxic or vasogenic edema on the examination used for quantitative analysis. Consistent with methodological approaches aimed at establishing reliable cohorts in pediatric neuroimaging studies, this review was intended to minimize the influence of potential confounding factors that could affect quantitative imaging measurements in both groups [29,30].

Exclusion Criteria Specific to the Epilepsy Group:

- Focal cortical dysplasia or other malformations of cortical development;
- Hippocampal sclerosis;
- Intracranial neoplasms potentially associated with epilepsy;
- Intracranial vascular malformations;
- Structural sequelae of previous cerebral infarction, intracranial hemorrhage, or traumatic brain injury;
- Encephalomalacia or focal gliosis;
- Major congenital brain anomalies;
- Intracranial structural abnormalities associated with neurocutaneous disorders;
- Hydrocephalus;
- Other structural brain lesions that could account for epilepsy;
- History of epilepsy surgery or other intracranial surgery.

Exclusion Criteria for Both Groups:

- Major congenital or acquired central nervous system abnormalities;
- Hydrocephalus or known disorders associated with alterations in intracranial pressure;
- History of central nervous system infection;
- Demyelinating or inflammatory central nervous system disorders;
- Previous cerebrovascular events;
- History of moderate or severe traumatic brain injury;
- History of intracranial surgery;
- Known systemic or neurological disorders that could affect EPVS, CP, or CSF compartments [5,6,7,23,26];
- Substantial anatomical deformities that could interfere with quantitative imaging analyses;
- Incomplete or insufficient clinical and/or imaging data;
- MRI examinations performed at an outside institution;
- Datasets unsuitable for reliable quantitative imaging analysis because of substantial motion artifacts, inadequate image quality, or inability to achieve anatomical validation during the quality-control process.

**Figure 1:**
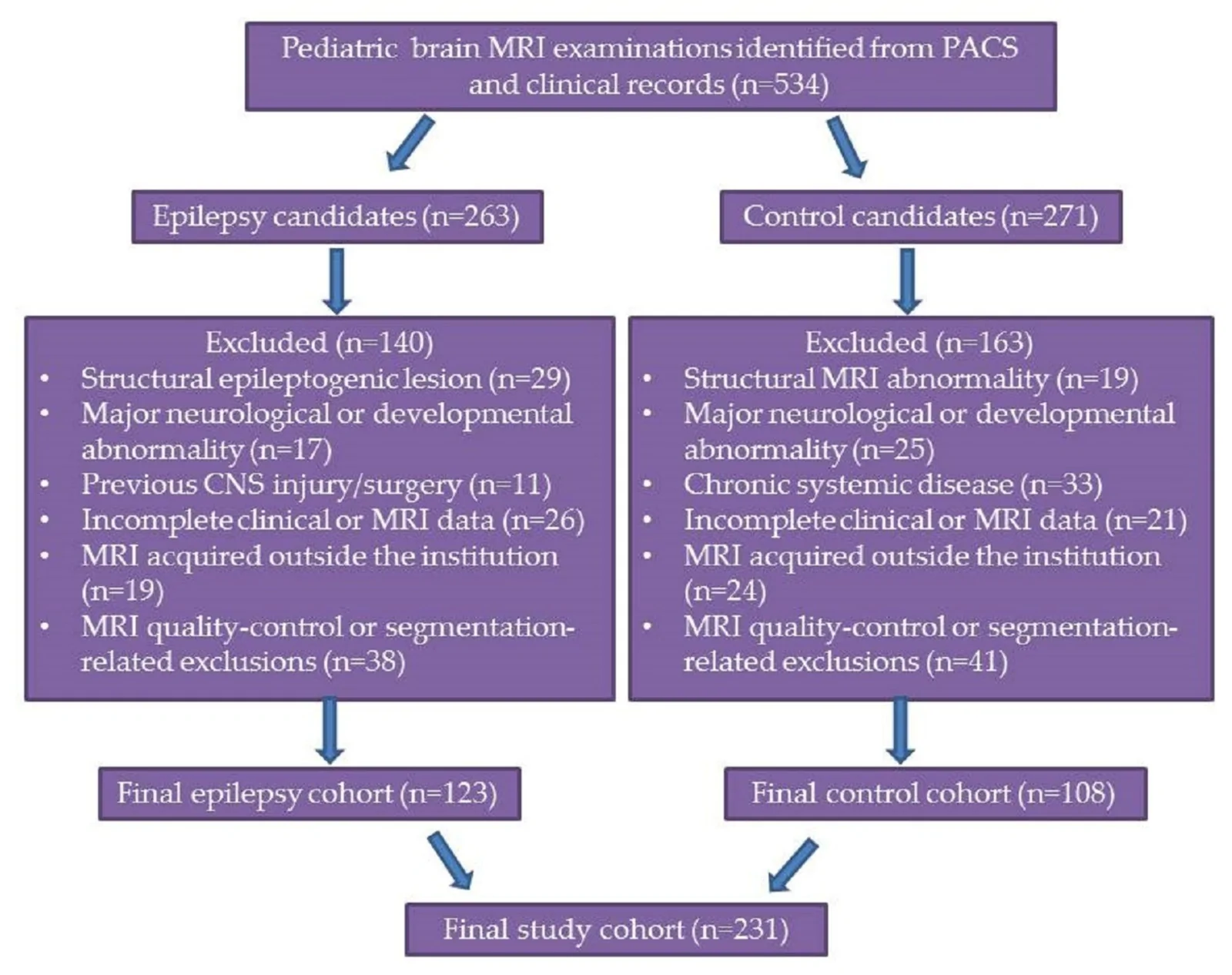
Flowchart of participant screening, eligibility assessment, exclusions, and final cohort formation. Pediatric brain MRI examinations identified from the institutional PACS and clinical records were screened according to predefined clinical, imaging, and quality-control criteria. The final study cohort comprised 123 children with epilepsy and 108 controls. Detailed reasons for exclusion and the corresponding numbers are provided within the flowchart. Abbreviations: CNS, central nervous system; MRI, magnetic resonance imaging; PACS, Picture Archiving and Communication System.

When more than one MRI examination was available for an otherwise eligible participant during the study period, the earliest examination satisfying all predefined clinical, acquisition, and image-quality eligibility criteria was designated as the index MRI. Each participant contributed only one index examination to the study; subsequent examinations from the same participant were not included in the quantitative dataset. The study population selection process, reasons for exclusion in each group, and the derivation of the final study cohorts are summarized in detail in Figure 1.

### 2.3. Clinical Variables

Demographic and epilepsy-related clinical data were obtained through retrospective review of institutional electronic medical records. Sex and age at the time of MRI examination were recorded for all participants. Because of the retrospective data structure, BMI/body-size measures, blood pressure, sleep-related variables, non-antiseizure medication use, and recent acute illness were not systematically and uniformly available across all participants. These variables were therefore not included as covariates in the between-group analyses. For the epilepsy group, age at epilepsy onset, disease duration, epilepsy type, drug resistance, number of antiseizure medications (ASMs) used at the time of MRI examination, and EEG findings were additionally recorded. However, the exact interval between the most recent seizure and MRI acquisition was not documented with sufficient consistency across all participants to establish a reliable, standardized minimum seizure-free interval for the epilepsy cohort. Epilepsy type was categorized as focal, generalized, combined generalized and focal, or unknown according to the ILAE 2017 classification [27]. Disease duration was calculated as the interval between the age at epilepsy onset and the date of MRI examination. The number of ASMs used at the time of MRI examination was recorded, and treatment was classified as monotherapy or polytherapy. Drug resistance was defined according to the ILAE consensus definition as failure to achieve sustained seizure freedom despite adequate trials of two appropriately chosen and tolerated ASM regimens, whether administered as monotherapy or in combination [31]. EEG findings were classified as focal, generalized, multifocal, or normal based on clinical EEG reports. To ensure temporal proximity between the electrophysiological and imaging assessments, EEG analyses were restricted to examinations performed within the 3 months preceding the index MRI, and when multiple eligible EEG examinations were available, the examination closest to the MRI date was selected. In patients with focal or multifocal epileptiform activity, EEG lateralization was categorized as right, left, or bilateral according to the hemispheric distribution of epileptiform activity, while anatomical localization was recorded as temporal, frontal, centroparietal/posterior, multifocal, or indeterminate.

### 2.4. MRI Acquisition Protocol

All brain MRI examinations were performed on the same 1.5-T MRI system (Ingenia, Philips Medical Systems, Best, The Netherlands) using the same standard 16-channel head coil. The technical consistency of the imaging protocol was assessed through retrospective review of scanner and protocol records covering the study period. No major hardware or software upgrades affecting the pediatric brain MRI acquisition protocol or the dedicated 3D sequences used for quantitative analysis were identified between 2018 and 2026. Minor software updates occurred intermittently as part of routine system maintenance; however, these updates were unrelated to the pediatric brain MRI acquisition protocol and did not result in changes to the dedicated isotropic 3D T1-weighted or 3D T2-FLAIR acquisitions used for quantitative analysis. No major hardware or software changes that could have affected the comparability of quantitative imaging analyses were identified during the study period.

The technical acquisition parameters of the brain MRI protocol were as follows:

- Axial T1-weighted spin-echo (SE): repetition time/echo time (TR/TE), 450/15 ms; field of view (FOV), 230 mm; slice thickness, 5 mm; matrix, 308 × 183.
- Axial fat-saturated T1-weighted SE: TR/TE, 633/15 ms; FOV, 230 mm; slice thickness, 5 mm; matrix, 308 × 183.
- Axial T2-weighted turbo spin-echo (TSE): TR/TE, 5240/100 ms; FOV, 230 mm; slice thickness, 5 mm; matrix, 384 × 237.
- Coronal T2-weighted TSE: TR/TE, 3027/100 ms; FOV, 200 mm; slice thickness, 3 mm; matrix, 336 × 217.
- Coronal T1-weighted inversion recovery: TR/TE, 3079/15 ms; FOV, 200 mm; slice thickness, 3.5 mm; matrix, 336 × 211.
- Coronal FLAIR: TR/TE, 11,000/130 ms; FOV, 230 mm; slice thickness, 3 mm; matrix, 256 × 157.
- Isotropic 3D T2-FLAIR: TR/TE/inversion time (TI), 4800/315/1650 ms; FOV, 250 mm; slice thickness, 1.0 mm; matrix, 216 × 218; native voxel dimensions, 1 × 1 × 1 mm^3^.
- Axial diffusion-weighted imaging (DWI): b-values, 0 and 1000 s/mm^2^; FOV, 230 mm; slice thickness, 5 mm; matrix, 152 × 106.
- Isotropic 3D T1-weighted imaging: TR/TE, 2500/4 ms; FOV, 230 mm; slice thickness, 1.0 mm; matrix, 256 × 256; native voxel dimensions, 1 × 1 × 1 mm^3^.

None of the participants in either the epilepsy or control group underwent pharmacological sedation or general anesthesia for MRI acquisition; all examinations included in the final cohort were therefore performed under non-sedated conditions. No participant received intravenous contrast material. The conventional two-dimensional sequences formed part of the routine clinical brain MRI protocol and were used for clinical structural assessment, whereas the high-resolution isotropic 3D acquisitions provided the datasets required for the quantitative imaging analyses. The conventional 5-mm axial T2-weighted acquisition was part of the routine clinical sequence set and should be distinguished from a dedicated high-resolution epilepsy MRI acquisition; it was not used as an input to the quantitative imaging analyses. The dedicated isotropic 3D T1-weighted and 3D T2-FLAIR acquisitions required for the quantitative workflow were consistently available for all participants included in the final cohort, and conventional thick-slice acquisitions were not substituted for these datasets in any participant. EPVS analysis was performed using the isotropic 3D T1-weighted and 3D T2-FLAIR datasets, whereas the isotropic 3D T1-weighted datasets were used for the remaining quantitative volumetric analyses. Conventional MRI sequences were additionally reviewed for structural assessment, image quality control, and anatomical validation.

### 2.5. Image Preprocessing

Eligibility of MRI examinations for the quantitative workflow was assessed according to predefined technical and anatomical quality criteria, including image quality and anatomical coverage, adequacy of the required 3D datasets, processing and registration integrity, segmentation reliability, and final anatomical validity. Because diagnostic group membership was available during retrospective clinical eligibility screening, the overall image-quality and processing-exclusion workflow was not considered formally blinded to group status. However, exclusion decisions were based on these predefined technical and anatomical criteria rather than on the direction or magnitude of subsequent between-group quantitative findings, which were not used as criteria for participant inclusion or exclusion.

**Figure 2:**
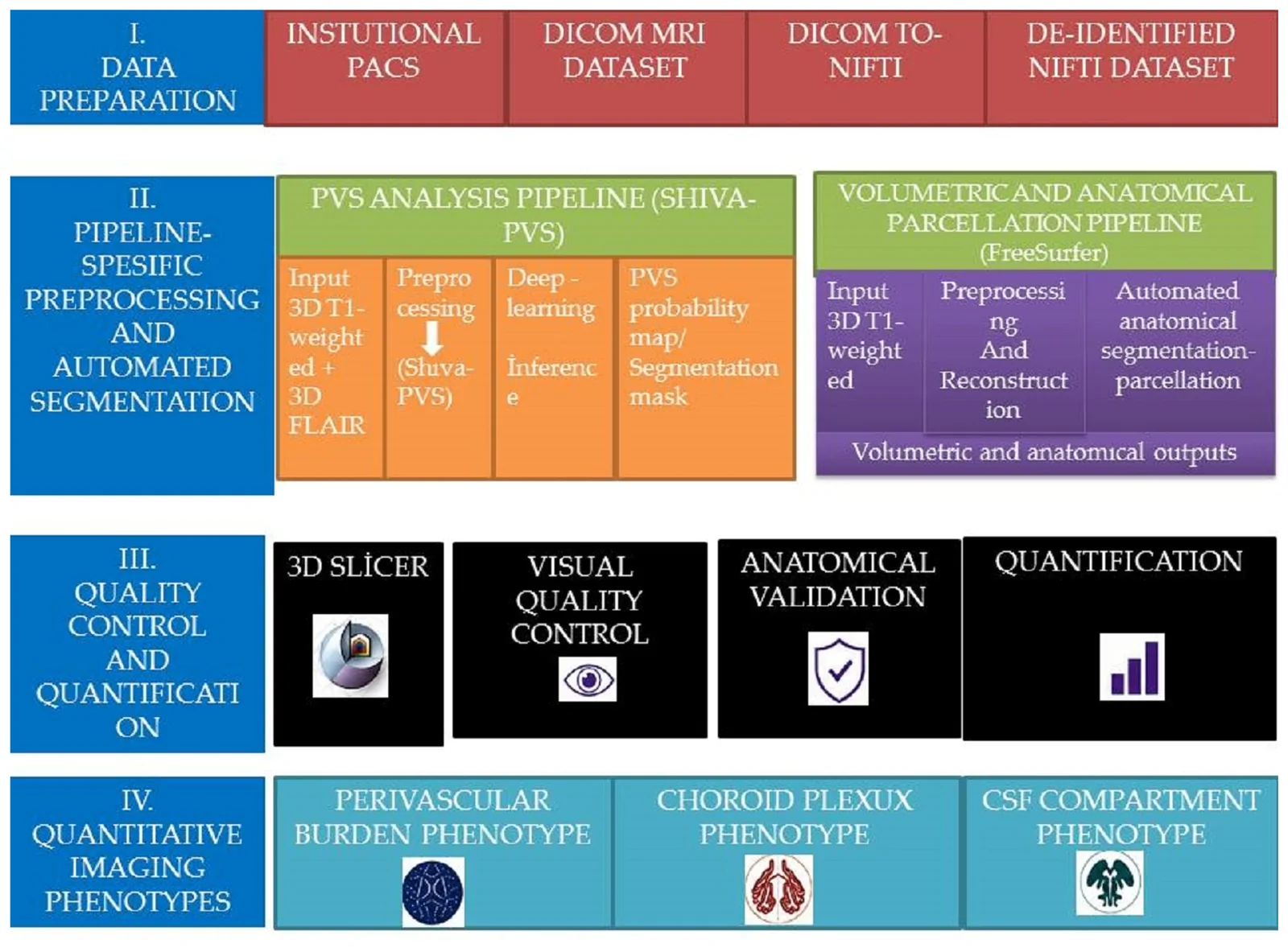
MRI processing and quantitative imaging analysis workflow. DICOM images were converted to NIfTI format and de-identified before quantitative analysis. SHIVA-PVS and FreeSurfer pipelines were used for automated EPVS segmentation and volumetric and anatomical segmentation/parcellation, respectively. Automated outputs underwent visual quality control, anatomical validation, and quantification in 3D Slicer, yielding EPVS burden, choroid plexus, and CSF compartment phenotypes. Abbreviations: CSF, cerebrospinal fluid; DICOM, Digital Imaging and Communications in Medicine; EPVS, enlarged perivascular spaces; MRI, magnetic resonance imaging; NIfTI, Neuroimaging Informatics Technology Initiative; PACS, Picture Archiving and Communication System; PVS, perivascular spaces.

Imaging datasets that successfully passed the quality-control process were anonymized and converted from Digital Imaging and Communications in Medicine (DICOM) format to Neuroimaging Informatics Technology Initiative (NIfTI) format using MRIcroGL (version 1.2.20220720; Chris Rodden, University of South Carolina, Columbia, SC, USA) before quantitative image analysis [32]. Quantitative image analysis was performed using three complementary image-processing approaches. EPVS analysis was performed using the automated SHIVA-PVS pipeline (v4 T1 + FLAIR-PVS; ResUNet3D, 5-fold model) [33]. Volumetric analyses of brain structures, CSF compartments, and the CP were performed using FreeSurfer (version 7.4.1; Martinos Center for Biomedical Imaging, Massachusetts General Hospital, Boston, MA, USA) [34,35]. Visual quality control, anatomical validation, and quantitative evaluation of the automated segmentation outputs were performed using 3D Slicer (version 5.10.0; Brigham and Women’s Hospital, Harvard Medical School, Boston, MA, USA) [36]. The image-processing and quantitative analysis workflow used in this study is summarized in Figure 2.

### 2.6. Image Processing

The image-processing workflow was structured to integrate complementary tools according to the analytical requirements of the different quantitative imaging components. The SHIVA-PVS pipeline (v4 T1+FLAIR-PVS; ResUNet3D, 5-fold model) was used because it enables deep learning-based EPVS segmentation across different MRI sequences and subsequent quantitative analysis using imaging software [33,35]. FreeSurfer was used for automated anatomical segmentation and volumetric analysis of brain structures and CSF compartments, quantitative assessment of CP volume, and generation of the anatomical parcellations required for regional EPVS analysis [34,37]. 3D Slicer was used for multiplanar and three-dimensional review of the automated segmentation outputs, visual assessment of their anatomical accuracy, and extraction of the required quantitative measurements from the segmentation masks [36].

#### 2.6.1. Automated EPVS Segmentation Using SHIVA-PVS, Visual Quality Control, and Manual Correction

The multimodal v4 T1+FLAIR-PVS model was used in this study. The model is based on a three-dimensional ResUNet3D architecture and performs inference using a five-fold model ensemble [33,35]. For each participant, isotropic 3D T1-weighted and 3D T2-FLAIR images were used as multimodal model inputs. T2-FLAIR images were co-registered to the T1-weighted image space, and both images were preprocessed according to the SHIVA-PVS analysis requirements. The co-registered T1-weighted and T2-FLAIR images were jointly provided to the multimodal model as two separate imaging channels. Model inputs were resampled to a standardized isotropic spatial resolution of 1 × 1 × 1 mm^3^, and voxel intensity values were normalized to the [0,1] range [33]. This standardized model-input resolution represents a preprocessing parameter separate from the native acquisition resolution reported in Section 2.4.

Segmentation inference was performed independently for each of the five folds, and an ensemble prediction was generated by averaging the fold-specific predictions. The resulting output was a probability map representing the probability of EPVS at each voxel as a continuous value within the [0,1] range. In this study, a probability threshold of 0.5 was applied to convert the probability maps into binary EPVS segmentation masks [33]. To facilitate reproducibility, the commands, software versions, and key processing parameters used to prepare the images for SHIVA-PVS analysis, perform model inference, combine predictions from the five folds, and generate the final EPVS masks are provided in detail in Supplementary Material S1.

The methodological basis of SHIVA-PVS was established in the original development and validation study, in which manual EPVS segmentations served as the reference standard [35]. In that study, voxel-level Sørensen–Dice coefficients of 0.51 and 0.66 were reported for the deep white matter and basal ganglia, respectively, while the corresponding cluster-level Dice coefficients were 0.64 and 0.71. Because these performance metrics were derived from the development and validation cohorts of the original SHIVA-PVS model, they were not considered performance estimates for the v4 T1+FLAIR-PVS model in the pediatric cohort of the present study.

The v4 T1+FLAIR-PVS model was used in its pretrained form, without cohort-specific retraining, transfer learning, or fine-tuning. Because the aim of this study was neither to develop a new segmentation algorithm nor to revalidate model performance, an independent manually segmented reference dataset was not generated and cohort-specific Dice coefficients were not calculated. Instead, to assess the anatomical validity of the automated segmentation outputs and their reliability before quantitative analysis, all cases underwent expert-supervised quality control and, when necessary, manual mask correction [38,39,40].

All EPVS probability maps and binary segmentation masks generated by SHIVA-PVS were systematically reviewed by a neuroradiologist with 9 years of experience using 3D Slicer. During this primary quality-control and manual-correction procedure, the neuroradiologist was blinded to participants’ epilepsy/control group assignment and clinical information. The maps and masks were overlaid on the source 3D T1-weighted and T2-FLAIR images and evaluated in the axial, coronal, and sagittal planes. The anatomical location and morphological characteristics of the automated masks were assessed in conjunction with their corresponding findings on the source images. Segmentations inconsistent with EPVS anatomy, erroneous labeling of ventricular or subarachnoid CSF spaces, masks extending into cortical sulci, false-positive foci related to imaging artifacts, and isolated segmentation clusters inconsistent with EPVS morphology were considered false positives and removed from the masks. Conversely, regions demonstrating morphological and anatomical characteristics consistent with EPVS on the source images but incompletely represented in the automated masks were manually completed.

**Figure 3:**
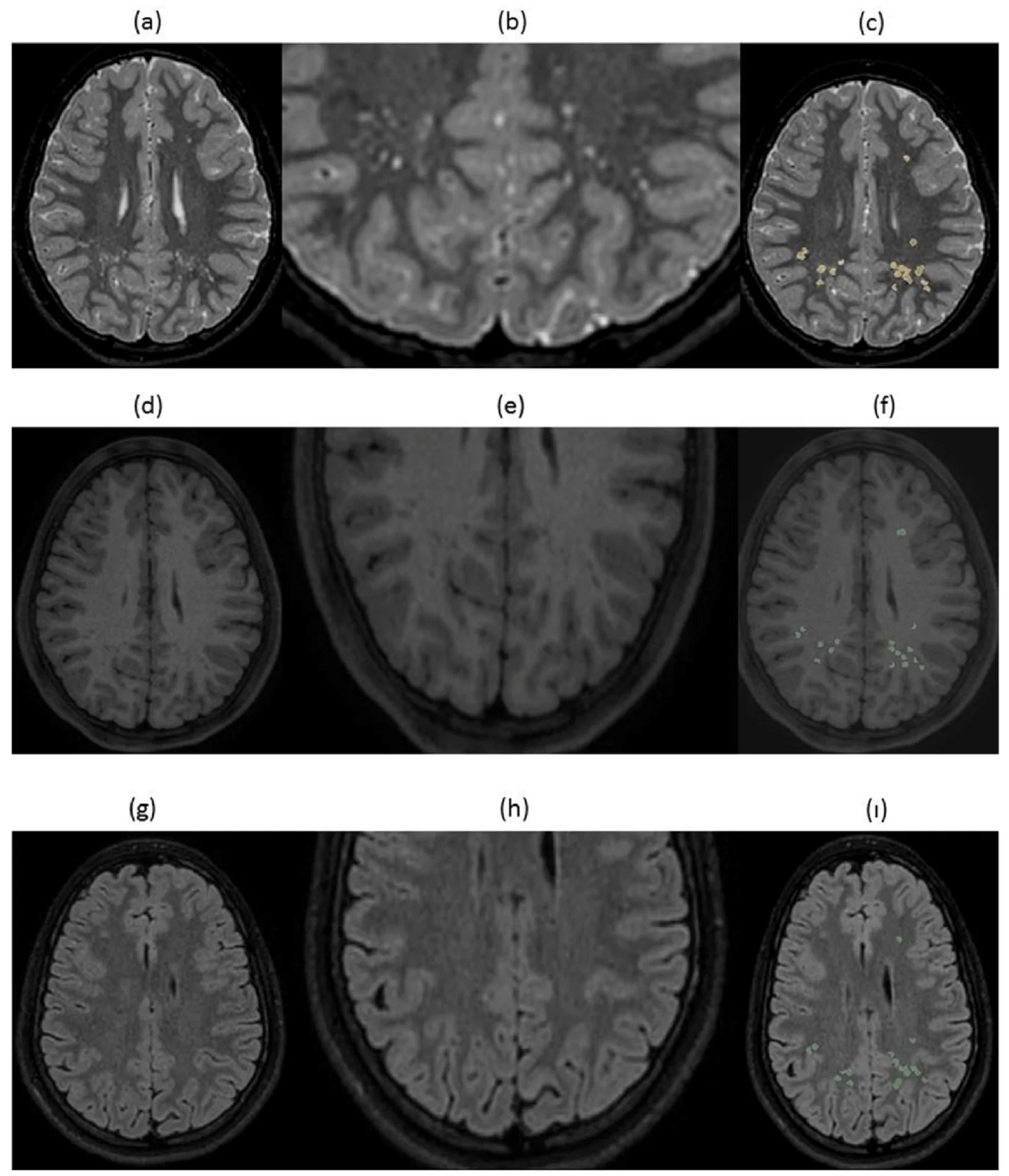
Representative MRI appearance, visual assessment, and SHIVA-PVS-assisted segmentation of enlarged perivascular spaces in a 13-year-old girl with epilepsy. All images are displayed in the axial plane. (**a**) T2-weighted image used for visual assessment of enlarged perivascular spaces (EPVS), with (**b**) a magnified view of a region showing a high concentration of visually identifiable EPVS and (**c**) the corresponding segmentation overlay used for visual quality and quantification control. (**d**) Raw 3D T1-weighted image and (**e**) magnified view of the EPVS-rich region, with (**f**) the corresponding SHIVA-PVS-assisted EPVS segmentation overlay. (**g**) Raw 3D T2-FLAIR image and (**h**) magnified view of the EPVS-rich region, with (**i**) the corresponding SHIVA-PVS-assisted EPVS segmentation overlay. The segmentation overlays demonstrate the spatial correspondence between EPVS identified on the source images and the segmented EPVS masks used in the image-analysis workflow. Abbreviations: EPVS, enlarged perivascular spaces; MRI, magnetic resonance imaging; SHIVA-PVS, Segmentation of Hippocampal subfields, Intracranial Volume, and perivascular spaces using deep learning; T2-FLAIR, T2-weighted fluid-attenuated inversion recovery.

All manual edits were performed by the same neuroradiologist using the segmentation tools available in 3D Slicer. Corrections were made only when there was clear anatomical inconsistency, an unequivocal false-positive segmentation, or incomplete EPVS representation; subjective expansion of the automated masks in equivocal regions was avoided. Quantitative analyses were performed exclusively on the final EPVS masks after completion of visual quality control and any necessary manual corrections. This procedure was implemented as an anatomical quality-control and standardization step before quantification rather than as a revalidation of the model. EPVS mapping derived from the final segmentation mask is illustrated in Figure 3 using a representative case from the epilepsy group.

#### 2.6.2. FreeSurfer-Based Automated Anatomical Segmentation and Volumetric Analysis

Automated segmentation and volumetric analysis of brain structures, ventricular CSF compartments, and the CP were performed using isotropic 3D T1-weighted images with FreeSurfer (version 7.4.1; Martinos Center for Biomedical Imaging, Massachusetts General Hospital, Boston, MA, USA) [34,37]. FreeSurfer is a widely used neuroimaging software package that performs cortical surface reconstruction, cortical anatomical parcellation, and automated segmentation of subcortical structures from isotropic 3D T1-weighted MRI within an integrated and standardized processing framework [37,41]. The ability to derive cortical and subcortical anatomical labels, ventricular CSF structures, and volumetric measurements from the same imaging dataset enables different neuroanatomical compartments to be evaluated within a common image-processing framework [37,41,42].

Cortical anatomical parcellation was performed using the Desikan–Killiany atlas, which is based on gyral and sulcal morphology [43]. In addition to cortical parcellation, subcortical structures, cerebral white matter, and the ventricular system were labeled using FreeSurfer’s automated anatomical segmentation approach [41]. This approach enabled volumetric assessment of brain structures and ventricular CSF compartments, as well as delineation of the anatomical regions required for subsequent regional analyses [37,41].

**Figure 4:**
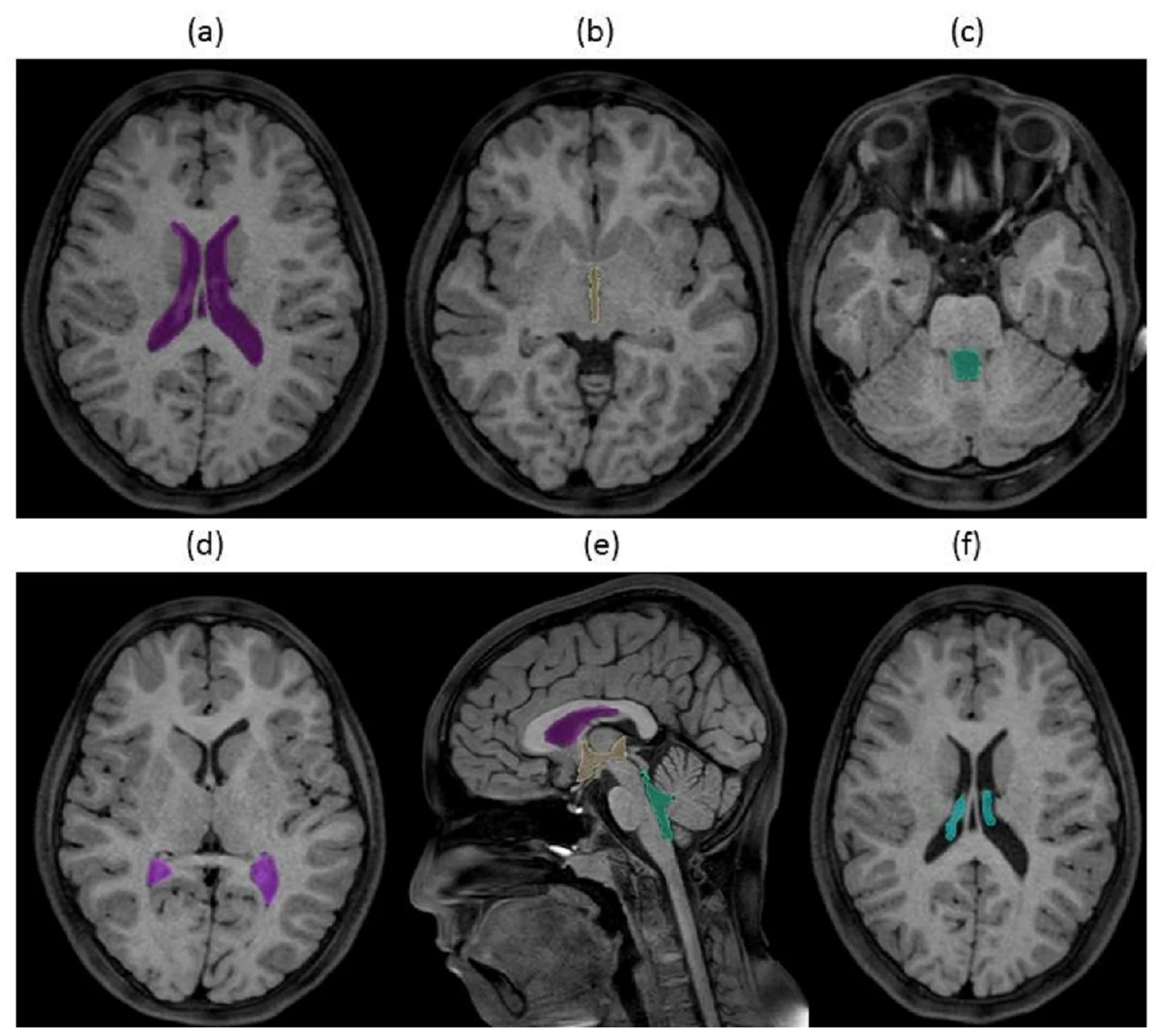
Representative FreeSurfer-based automated segmentation of ventricular cerebrospinal fluid compartments and the choroid plexus in a 15-year-old boy with epilepsy. FreeSurfer-derived anatomical labels are overlaid on the corresponding 3D T1-weighted images to demonstrate the structures included in the volumetric analyses. (**a**) Axial image showing the FreeSurfer-derived lateral ventricular segmentations in purple at the level of the bilateral ventricular trigones. (**b**) Axial image showing the third ventricle in beige. (**c**) Axial image showing the fourth ventricle in turquoise-green. (**d**) Axial image demonstrating the bilateral inferior lateral ventricles in light purple. (**e**) Sagittal image providing a combined anatomical overview of the ventricular compartments, including the lateral ventricle (purple), third ventricle (beige), and fourth ventricle (turquoise-green). (**f**) Axial image showing the bilateral choroid plexus segmentations in cyan. These FreeSurfer-derived segmentations underwent visual quality control and anatomical validation on the source 3D T1-weighted images before the corresponding volumetric measurements were included in the quantitative analyses. Abbreviations: CP, choroid plexus; CSF, cerebrospinal fluid.

For volumetric assessment of the ventricular CSF compartments, segmentations of the bilateral lateral ventricles and inferior lateral ventricles, as well as the third and fourth ventricles, were used. For bilateral structures, right- and left-sided volumes were recorded separately and retained to allow calculation of total bilateral volumes. FreeSurfer’s standard volumetric anatomical labeling system defines the lateral and inferior lateral ventricles and the third and fourth ventricles as distinct anatomical structures [41]. For volumetric assessment of the CP, the automatically generated right and left CP segmentations provided by FreeSurfer were used [34]. CP volumes for each hemisphere were obtained separately and recorded to allow calculation of total bilateral CP volume. Volumetric measurements of all anatomical structures were recorded as absolute volumes in mm^3^. In addition, estimated total intracranial volume (eTIV) derived from FreeSurfer was recorded to account for interindividual differences in intracranial volume in subsequent quantitative analyses [37]. The key processing parameters and commands used in the FreeSurfer-based image-processing workflow are provided in detail in Supplementary Material S2 to support reproducibility. FreeSurfer-derived automated anatomical segmentation, including the ventricular CSF compartments and CP segmentations, is illustrated in Figure 4 using a representative case from the epilepsy group.

#### 2.6.3. Quantitative Analysis of Regional EPVS Burden

To evaluate the regional distribution of EPVS within an anatomically standardized framework, EPVS segmentations generated by SHIVA-PVS were analyzed in conjunction with FreeSurfer-based anatomical segmentation and parcellation outputs. Whole-brain EPVS segmentations were separated by hemisphere to allow independent assessment of EPVS burden in the right and left cerebral hemispheres. Regional analysis was performed at the level of the basal ganglia (BG) and centrum semiovale (CSO), which represent the principal and most established anatomical regions for EPVS assessment on MRI [23,26,44]. This approach was used to anatomically partition whole-brain EPVS segmentations and generate hemisphere- and region-specific EPVS masks for subsequent quantitative analyses.

For hemispheric partitioning, the right and left cerebral hemisphere segmentations generated by FreeSurfer were used as anatomical references. Right- and left-hemisphere EPVS masks were generated by intersecting the whole-brain EPVS segmentation with the corresponding hemispheric anatomical masks. For regional anatomical delineation, subcortical anatomical segmentations and cerebral white matter segmentations derived from each participant’s isotropic 3D T1-weighted images using FreeSurfer served as anatomical references. BG masks were generated separately for the right and left hemispheres by combining the automated anatomical segmentations of the caudate nucleus, putamen, and globus pallidus within each hemisphere; the thalamus was not included in the BG. Thus, hemisphere-specific BG anatomical masks were obtained from the union of these three deep gray matter structures.

For delineation of the CSO, the right and left supratentorial cerebral white matter segmentations generated by FreeSurfer were used as the anatomical basis. The inferior boundary of the CSO was defined according to the most superior extent of an anatomical reference mask comprising the lateral ventricles and corpus callosum [44]. Cerebral white matter located superior to this boundary was identified separately in the right and left hemispheres to generate hemisphere-specific CSO anatomical masks.

**Figure 5:**
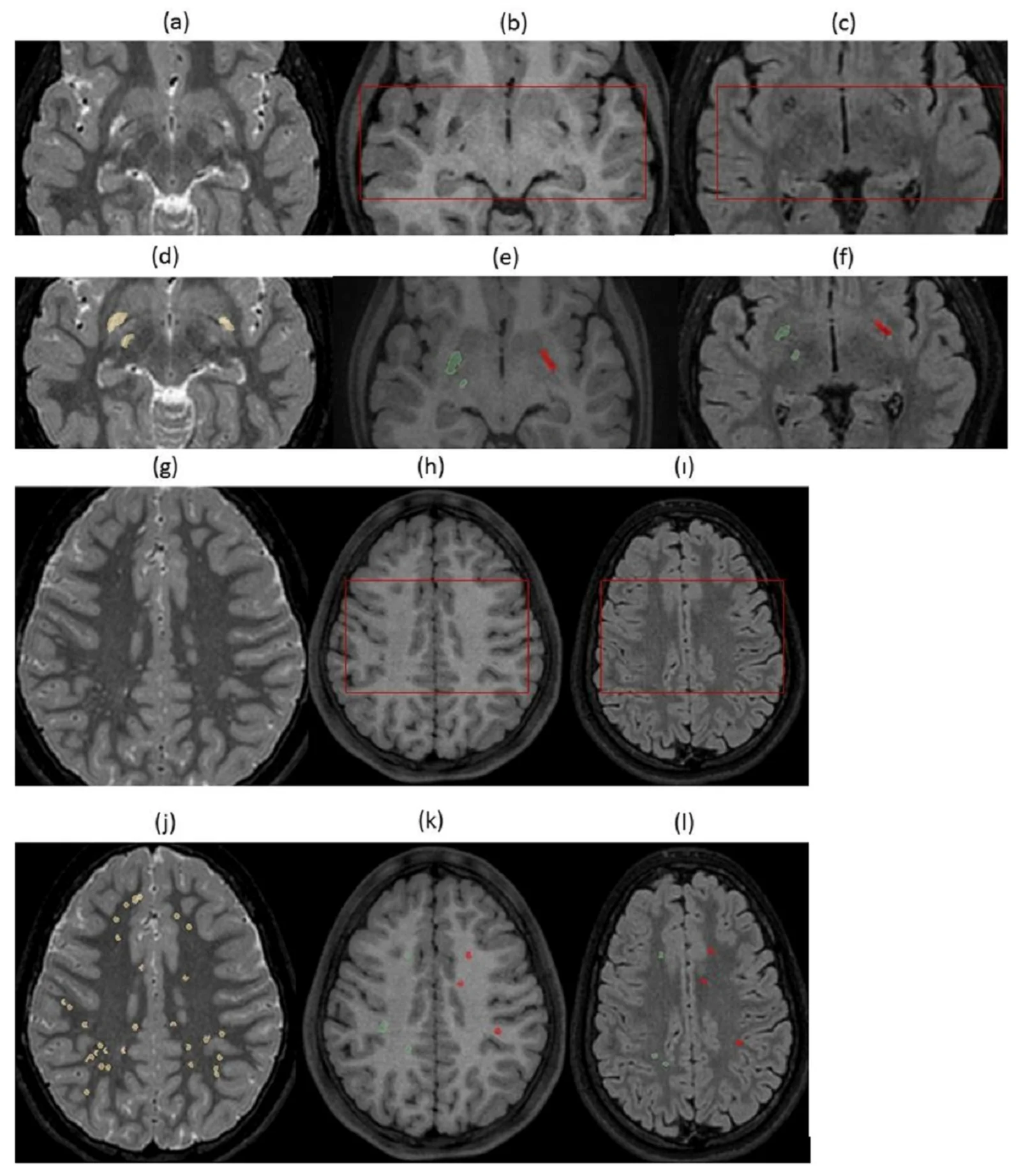
Representative anatomical regionalization and hemisphere-specific segmentation of enlarged perivascular spaces in the basal ganglia and centrum semiovale in an 11-year-old boy with epilepsy. All images are displayed in the axial plane. The upper two rows (**a**–**f**) illustrate basal ganglia (BG) assessment. (**a**) T2-weighted image demonstrates visually identifiable enlarged perivascular spaces (EPVS) at the level of the BG. Corresponding 3D T1-weighted (**b**) and 3D T2-FLAIR (**c**) images demonstrate the anatomical reference boundaries used to illustrate the regional extent of BG assessment (red rectangular outlines). (**d**) For visual presentation only, the final EPVS segmentation mask is displayed as an overlay on the conventional axial T2-weighted image. On the 3D T1-weighted (**e**) and 3D T2-FLAIR (**f**) images, colored overlays demonstrate hemisphere-specific BG-EPVS segmentations. The lower two rows (**g**–**l**) illustrate centrum semiovale (CSO) assessment. (**g**) T2-weighted image demonstrates visually identifiable EPVS at the level of the CSO. Corresponding 3D T1-weighted (**h**) and 3D T2-FLAIR (**i**) images demonstrate the anatomical reference boundaries used to illustrate the regional extent of CSO assessment (red rectangular outlines). (**j**) For visual presentation only, the final EPVS segmentation mask is displayed as an overlay on the conventional axial T2-weighted image. On the 3D T1-weighted (**k**) and 3D T2-FLAIR (**l**) images, colored overlays demonstrate hemisphere-specific CSO-EPVS segmentations. Green and red overlays distinguish right- and left-hemispheric regional EPVS segmentations, respectively. Yellow overlays in (**d**,**j**) highlight EPVS without hemisphere-specific color coding. Regional BG-EPVS and CSO-EPVS masks used for quantitative analysis were generated after geometric alignment by voxelwise intersection of the final anatomically validated EPVS segmentation with the corresponding FreeSurfer-derived anatomical masks. The conventional 5-mm axial T2-weighted sequence was used only for visual presentation in this figure and was not part of the SHIVA-PVS segmentation or quantitative regionalization pipeline. Abbreviations: BG, basal ganglia; CSO, centrum semiovale; EPVS, enlarged perivascular spaces; MRI, magnetic resonance imaging; T2-FLAIR, T2-weighted fluid-attenuated inversion recovery.

Before regional EPVS partitioning, the FreeSurfer-based anatomical masks were resampled to the image geometry of the final SHIVA-PVS mask using nearest-neighbor interpolation. After geometric alignment was verified, regional EPVS masks were generated by voxelwise intersection of the final EPVS mask with the corresponding anatomical masks. EPVS segmentations located within the BG anatomical masks were classified as BG-EPVS, whereas those located within the CSO anatomical masks were classified as CSO-EPVS. This procedure was performed separately for each hemisphere, yielding right and left BG-EPVS masks and right and left CSO-EPVS masks. The final regional EPVS segmentations at the BG and CSO levels are illustrated in Figure 5 using a representative case from the epilepsy group.

Regional EPVS analysis was restricted to the BG and CSO. This selection was based not only on their status as established anatomical regions for assessing EPVS burden on MRI, but also on the ability of the SHIVA-PVS segmentation approach combined with FreeSurfer-based anatomical regionalization to delineate these regions in a standardized and reproducible manner across the study cohort [26,44,45]. Other EPVS locations, including the temporal and hippocampal regions and the brainstem, were not included in region-specific analyses because, within the current imaging protocol and automated segmentation–anatomical regionalization framework, they could not be defined as standardized regions with a level of consistency comparable to that achieved for the BG and CSO [44,45]. EPVS segmentations potentially located within these regions were therefore not used to derive independent regional EPVS phenotypes.

The processing steps, key parameters, and commands used to generate the hemispheric, BG, and CSO anatomical masks, anatomically align the SHIVA-PVS and FreeSurfer outputs, and derive hemisphere- and region-specific EPVS masks are provided in detail in Supplementary Material S3 to support transparency and reproducibility of the analytical workflow.

#### 2.6.4. Imaging Quality Control and Volumetric Analysis Using 3D Slicer

Following completion of automated segmentation and regional partitioning, final quality control and anatomical validation of the imaging outputs, as well as volumetric analysis of the EPVS segmentations, were performed using 3D Slicer (version 5.10.0; Surgical Planning Laboratory, Brigham and Women’s Hospital, Boston, MA, USA) [36]. The source 3D T1-weighted and 3D T2-FLAIR images were evaluated together with the SHIVA-PVS- and FreeSurfer-based segmentation outputs. Segmentation masks were overlaid on the source anatomical images and reviewed along the x, y, and z axes in the axial, coronal, and sagittal planes. This assessment was performed to verify the anatomical validity of the automated segmentations and to determine the final imaging outputs to be used in quantitative analyses.

EPVS segmentations generated by SHIVA-PVS were visually assessed by the same experienced neuroradiologist for spatial and morphological concordance with the source anatomical images. Following quality control and any necessary manual corrections, the final anatomically validated EPVS masks were included in the quantitative analysis. Volumetric analysis of the EPVS segmentations was performed using the Segment Statistics module in 3D Slicer. Volumes of the final whole-brain EPVS mask, right and left hemispheric EPVS masks, and hemisphere-specific BG-EPVS and CSO-EPVS masks were calculated in mm^3^. Right and left hemispheric EPVS volumes were recorded separately. Right and left BG-EPVS and CSO-EPVS volumes were also obtained separately, and bilateral regional volumes were calculated as the sum of the corresponding hemispheric measurements. These volumetric measurements were used for quantitative assessment of EPVS burden at the whole-brain and regional levels.

**Figure 6:**
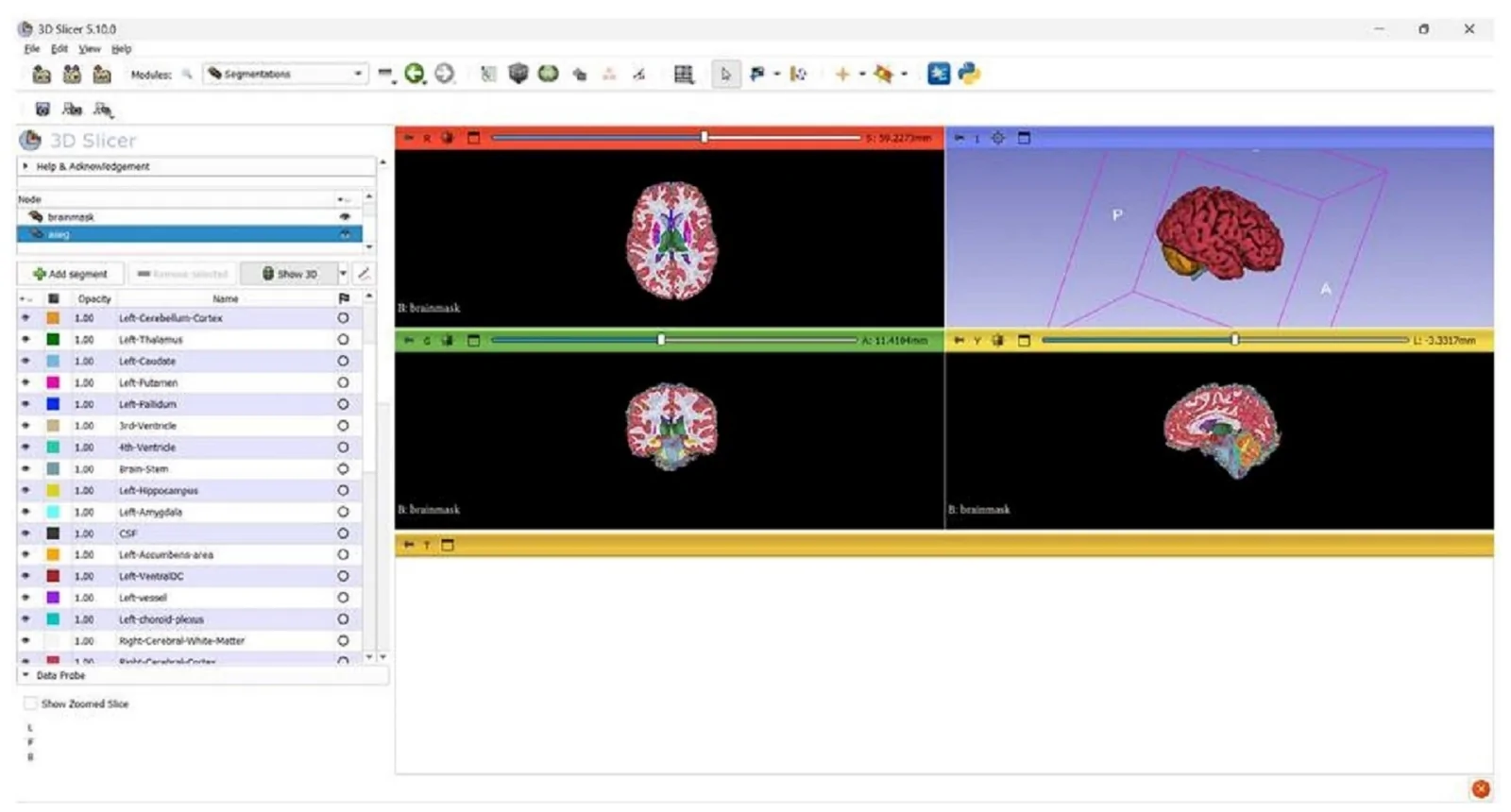
Representative visual quality control and anatomical validation of FreeSurfer-derived segmentation and parcellation outputs in 3D Slicer. FreeSurfer-derived anatomical labels are overlaid on the source 3D T1-weighted image and simultaneously reviewed in axial, coronal, and sagittal planes, together with three-dimensional surface rendering, to assess spatial correspondence and anatomical plausibility of the automated segmentation outputs. Multiplanar inspection enables evaluation of segmentation boundaries, structural continuity, and conformity of labeled structures with the underlying anatomy, while the three-dimensional view provides complementary assessment of the overall spatial configuration of the reconstructed anatomical structures. This quality-control procedure was performed before quantitative analysis to identify substantial anatomical discrepancies or segmentation failures that could compromise the reliability of volumetric measurements. Abbreviations: 3D, three-dimensional.

**Figure 7:**
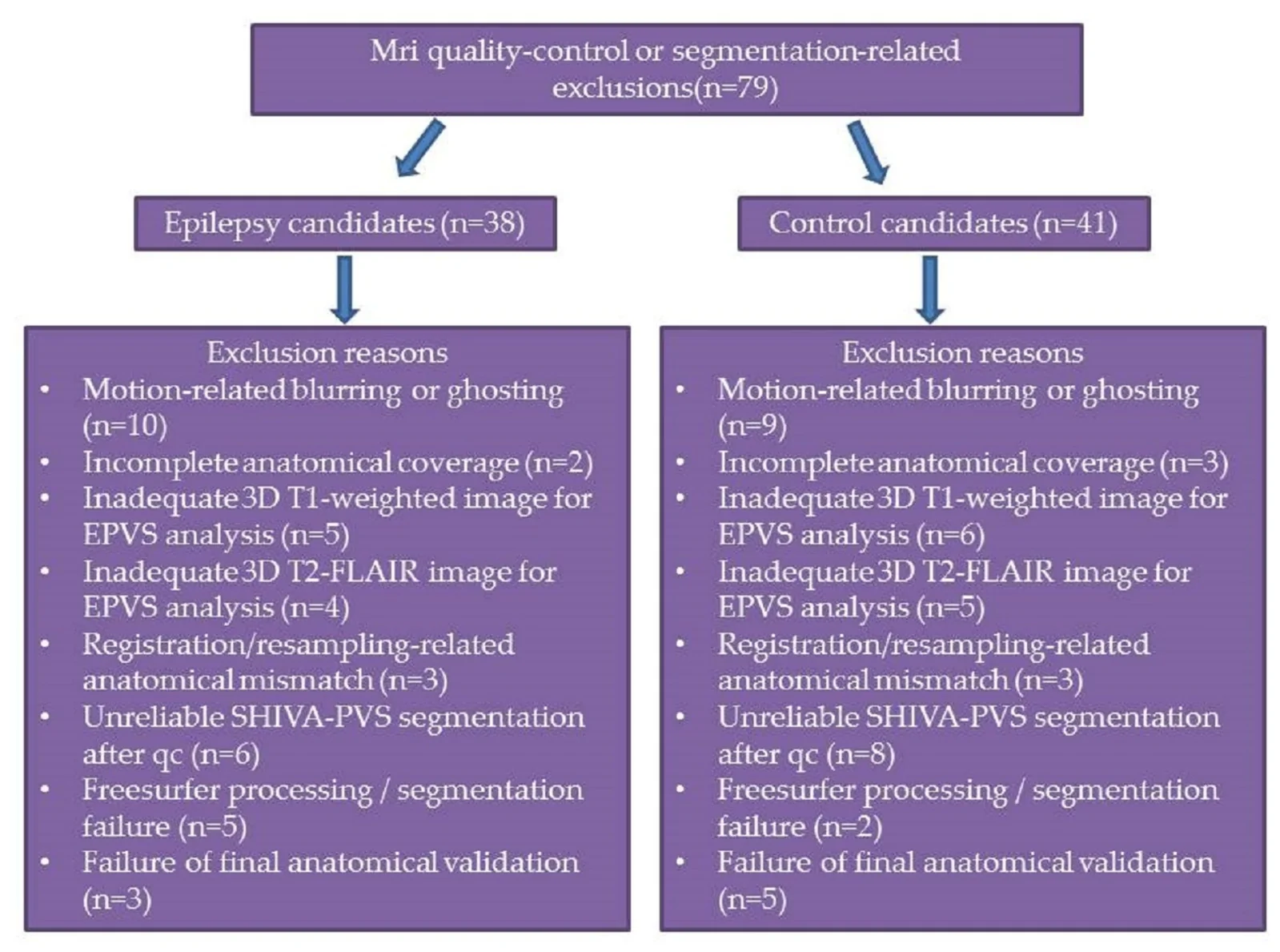
Detailed distribution of MRI quality-control and segmentation-related exclusions. Of the 79 examinations excluded because of image-quality, processing, segmentation, or anatomical-validation limitations, 38 were epilepsy candidates and 41 were control candidates. Specific reasons for exclusion and the corresponding numbers in each group are provided within the flowchart and include motion-related blurring or ghosting, incomplete anatomical coverage, inadequate 3D T1-weighted or 3D T2-FLAIR images for EPVS analysis, registration/resampling-related anatomical mismatch, unreliable SHIVA-PVS segmentation after quality control, FreeSurfer processing or segmentation failure, and failure of final anatomical validation. Abbreviations: EPVS, enlarged perivascular spaces; MRI, magnetic resonance imaging; QC, quality control; SHIVA-PVS, Segmentation of Hippocampal subfields, Intracranial Volume, and perivascular spaces using deep learning; T2-FLAIR, T2-weighted fluid-attenuated inversion recovery.

FreeSurfer-generated anatomical segmentations were overlaid on the source 3D T1-weighted images and subjected to visual quality control in the axial, coronal, and sagittal planes using 3D Slicer [37,41]. Particular attention was given to the anatomical structures included in the quantitative analyses, especially the CP and ventricular CSF compartments, with assessment of boundary conformity, segmentation integrity, and their relationship to adjacent anatomical structures. The suitability of the volumetric outputs provided by FreeSurfer for quantitative analysis was determined by visually confirming the anatomical validity of the underlying segmentations. Examinations with substantial anatomical discrepancies or segmentation failures that precluded reliable quantitative assessment were excluded from the analysis. A representative example of visual quality control of FreeSurfer-based segmentations in 3D Slicer is shown in Figure 6. The distribution of examinations considered unsuitable for quantitative assessment because of image quality or segmentation reliability, stratified by specific reasons for exclusion, is presented in Figure 7.

### 2.7. Definition of Quantitative Imaging Phenotypes

#### 2.7.1. Enlarged Perivascular Space (EPVS) Phenotype

The EPVS phenotype was structured to characterize global and hemispheric EPVS burden, regional EPVS burden, right–left asymmetry, and the relative distribution of EPVS burden across anatomical regions [26,35,44,45]. This approach was designed to quantify not only the absolute magnitude of EPVS burden but also its hemispheric lateralization and its distribution between the BG and CSO regions [26,44,45]. The imaging biomarkers used in the analysis to characterize the EPVS phenotype are summarized in Table 1.

Total EPVS volume was used as the principal measure of global EPVS burden. Bilateral regional aggregate measures were used as anatomically defined complementary measures of regional burden, whereas hemispheric and lateralized regional measures, asymmetry indices, relative regional burden measures, and CSO-to-BG ratios were used to characterize complementary or supportive dimensions of the EPVS phenotype, including anatomical distribution and lateralization, rather than as statistically independent co-primary outcomes. The regional EPVS phenotype was defined on the basis of EPVS volumes derived from the right and left CSO and BG regions [26,44,45]. Total bilateral CSO-EPVS and BG-EPVS volumes were calculated by summing the corresponding right and left regional volumes. This framework enabled characterization of global EPVS burden together with its regional distribution and lateralization while explicitly recognizing the mathematical relationships among several derived measures [16,18,26,44,45].

To quantitatively assess the lateralization of EPVS burden between the hemispheres, asymmetry indices (AI) were calculated at the hemispheric, CSO, and BG levels [18]. For each anatomical level, the directional asymmetry index was calculated using the formula AI = (R − L)/(R + L), where *R* and *L* represent the corresponding right and left EPVS volumes, respectively. An AI of zero indicates bilateral symmetry, positive values indicate right-sided predominance, and negative values indicate left-sided predominance. An increasing absolute AI value reflects a greater degree of lateralization. These measures enabled assessment of both the direction and magnitude of interhemispheric distribution, independently of the overall magnitude of EPVS burden.

To evaluate the anatomical distribution of EPVS burden relative to global and hemispheric EPVS burden, relative EPVS burden measures were calculated [26,44]. At the hemispheric level, CSO-EPVS and BG-EPVS volumes were divided by the total EPVS volume within the same hemisphere to determine the relative contribution of each anatomical region to the hemispheric EPVS burden. At the whole-brain level, total bilateral CSO-EPVS and BG-EPVS volumes were divided by total EPVS volume. Similarly, right and left hemispheric EPVS volumes were divided by total EPVS volume to determine the relative contribution of each hemisphere to the global EPVS burden. In addition to absolute EPVS volumes, these measures enabled quantitative characterization of the anatomical distribution of EPVS burden.

Finally, a separate CSO-to-BG EPVS volume ratio was calculated for each hemisphere to assess the relative distribution of EPVS burden between the CSO and BG regions [26,44,45]. This measure, calculated by dividing the CSO-EPVS volume by the BG-EPVS volume within the same hemisphere, was used to characterize the relative distribution of EPVS burden between the CSO and deep gray matter compartments. Calculating the CSO-EPVS-to-BG-EPVS ratio within each hemisphere enabled hemisphere-specific characterization of the relative distribution of EPVS burden between these two anatomical compartments. An additional total bilateral CSO-EPVS-to-BG-EPVS ratio was not calculated because both relative total CSO-EPVS and relative total BG-EPVS burdens were normalized to the same total EPVS volume; consequently, the ratio of these two measures is mathematically equivalent to the total CSO-EPVS/total BG-EPVS ratio. Therefore, an additional bilateral CSO-to-BG EPVS ratio was considered unlikely to provide distributional information beyond an algebraic re-expression of the existing relative measures. A ratio > 1 indicates relative CSO predominance, a ratio < 1 indicates relative BG predominance, and values approaching 1 indicate a more balanced EPVS distribution between the two compartments. For ratio and asymmetry measures, variables with a zero denominator were not calculated and were designated as missing values (NA); no constant addition or imputation was applied in these cases.

#### 2.7.2. Choroid Plexus (CP) Phenotype

The CP phenotype was structured to characterize absolute CP volume, CP volume normalized to intracranial volume, hemispheric CP asymmetry, and the relative distribution of CP volume in relation to the corresponding ventricular CSF compartments. This approach was designed to quantitatively assess not only the absolute volumetric size of the CP but also its normalized volume after accounting for interindividual differences in intracranial volume, its right–left lateralization, and its proportional characteristics relative to ventricular system size [17,24,34]. Right and left CP volumes derived from FreeSurfer, together with measurements of the lateral and inferior lateral ventricles, third ventricle, fourth ventricle, and eTIV, were used to derive these phenotypic measures [34,37,41]. The imaging biomarkers used in the analysis to characterize the CP phenotype are summarized in Table 2.

Absolute CP measures were defined as right and left CP volumes and total CP volume, calculated as the sum of the two hemispheric measurements. All absolute CP volumes were recorded in mm^3^. To reduce the potential influence of interindividual differences in intracranial volume on CP measurements, total, right, and left CP volumes were normalized to eTIV [46]. eTIV-normalized total CP volume was calculated by dividing total CP volume by eTIV, whereas hemisphere-specific normalized CP volumes were calculated by dividing the corresponding right or left CP volume by eTIV [46]. Thus, in addition to absolute CP volumes, the relative volumetric burden of CP tissue within the individual’s total intracranial volume was assessed.

To evaluate hemispheric lateralization of CP volume, a directional CP asymmetry index was calculated using the formula AI = (R − L)/(R + L), where *R* and *L* represent right and left CP volumes, respectively. An AI of zero indicates bilateral symmetry, positive values indicate right-sided predominance, and negative values indicate left-sided predominance. To quantify the magnitude of asymmetry irrespective of its direction, the absolute asymmetry index |AI| was also calculated. Accordingly, directional AI represented the direction of lateralization, whereas |AI| represented the degree of lateralization regardless of the predominant hemisphere. Because these two measures were derived from the same underlying volumes, they were not considered independent anatomical markers but rather complementary measures characterizing the directional and magnitude components of CP asymmetry.

To evaluate CP volume relative to the size of the anatomically corresponding lateral ventricular compartment, hemisphere-specific CP-to-lateral ventricle volume ratios were calculated [34]. Right CP volume was divided by right lateral ventricle volume, and left CP volume by left lateral ventricle volume, yielding hemisphere-specific ratios [34]. In addition, the total CP-to-total lateral ventricle volume ratio was calculated by dividing total bilateral CP volume by the sum of the right and left lateral ventricle volumes. These measures were not intended to characterize CP size independently of ventricular volume but rather to provide a proportional assessment of CP volume relative to the size of the anatomically corresponding lateral ventricular compartment.

Finally, the total CP-to-total ventricular CSF volume ratio was calculated to evaluate CP volume relative to the broader ventricular CSF compartment. Total ventricular CSF volume was defined as the sum of the bilateral lateral ventricular, bilateral inferior lateral ventricular, third ventricular, and fourth ventricular volumes [37,41]. The ratio of total CP volume to this aggregate ventricular CSF volume was considered a complementary imaging biomarker characterizing the volumetric relationship between global CP volume and the ventricular CSF compartment [24].

The CP-to-lateral ventricle and CP-to-total ventricular CSF ratios were defined not as substitutes for CP volume measurements but as complementary imaging biomarkers that may provide additional information beyond absolute and eTIV-normalized CP volumes. Accordingly, the CP phenotype was evaluated within a multidimensional quantitative framework encompassing absolute volume, intracranial volume–normalized volume, hemispheric asymmetry, and relative volumetric relationships between the CP and ventricular CSF compartments. For ratio and asymmetry measures with a zero denominator, the corresponding variable was not calculated and was designated as missing (NA); no constant addition or imputation was applied in such cases.

#### 2.7.3. Cerebrospinal Fluid (CSF) Phenotype

The ventricular CSF phenotype was defined to characterize the compartment-specific volumetric features of the ventricular system. Accordingly, right and left lateral ventricular volumes, right and left inferior lateral ventricular volumes, and third and fourth ventricular volumes derived from FreeSurfer were analyzed [37,41,47]. Given that the ventricular system represents one of the principal anatomical compartments of CSF distribution, these measures were considered not as direct indicators of glymphatic dysfunction but as structural imaging biomarkers potentially associated with glymphatic dysfunction and were evaluated alongside EPVS and CP measures in this study [3,4,23,24,25]. Analyses were performed at three levels: absolute ventricular volumes, eTIV-normalized ventricular volumes, and bilateral ventricular asymmetry. The quantitative measures evaluated in the ventricular CSF analysis and their definitions are presented in Table 3.

Right and left lateral ventricular volumes, right and left inferior lateral ventricular volumes, and third and fourth ventricular volumes were recorded in mm^3^ [37,41,47]. Given that ventricular volumes may be influenced by intracranial size, the volume of each compartment was additionally normalized to eTIV [28,29,47]. This enabled assessment of the relative size of the ventricular compartments with respect to intracranial volume in addition to their absolute volumes. Total bilateral lateral ventricular and inferior lateral ventricular volumes were calculated as the sum of the corresponding right and left compartment volumes. In addition, total ventricular CSF volume was defined as the sum of the bilateral lateral ventricular, bilateral inferior lateral ventricular, third ventricular, and fourth ventricular volumes.

To characterize right–left volume differences in the bilateral lateral and inferior lateral ventricles independently of absolute ventricular size, directional asymmetry indices were calculated [48]. The lateral ventricular and inferior lateral ventricular asymmetry indices were defined as (R_LV − L_LV)/(R_LV + L_LV) and (R_InfLV − L_InfLV)/(R_InfLV + L_InfLV), respectively [48,49]. A value of zero indicates bilateral symmetry, positive values indicate right-sided predominance, and negative values indicate left-sided predominance, whereas an increasing absolute value of the index reflects a greater degree of asymmetry. These measures were used as complementary metrics to absolute and eTIV-normalized ventricular volumes for characterizing the bilateral distribution of ventricular volume.

### 2.8. Interobserver Reproducibility Analysis

To assess the interobserver reproducibility of quantitative imaging measurements and the reproducibility of visual quality control and, when necessary, manual corrections applied to EPVS segmentations, an independent second-observer analysis was performed by a neuroradiologist with 7 years of experience in a randomly selected subset of 40 participants, comprising 20 participants each from the epilepsy and control groups. Within each diagnostic group, participants were selected randomly without selection based on EPVS burden, age, sex, or imaging findings. For EPVS assessment, both observers independently reviewed the same source 3D T1-weighted and 3D T2-FLAIR images and the initial SHIVA-PVS segmentations while blinded to participants’ clinical information, group assignment, and the other observer’s assessments. The same visual quality-control and manual correction criteria defined for the primary analysis were applied to all EPVS assessments. This analysis was designed to evaluate the reproducibility of the observer-based quality-control and manual correction step performed after automated segmentation rather than to revalidate the segmentation performance of the SHIVA-PVS model.

From the final EPVS masks independently reviewed and manually corrected as necessary by each observer, total, right and left hemispheric, right and left BG-EPVS, and right and left CSO-EPVS volumes were obtained. Interobserver volumetric reproducibility of CP and ventricular CSF segmentations was also assessed in the same participant subset. For these measurements, both observers independently started from the same original FreeSurfer-derived automated segmentations and performed anatomical validation and, when necessary, correction using the same anatomical definitions and quality-control criteria applied in the primary analysis, without access to the other observer’s final quality-controlled segmentations. Observer-specific final segmentations were then used to obtain right, left, and total CP volumes; right, left, and total lateral ventricular volumes; right, left, and total inferior lateral ventricular volumes; third and fourth ventricular volumes; and total ventricular CSF volume. Interobserver agreement for these quantitative volumetric measurements of EPVS, CP, and ventricular CSF was assessed using the intraclass correlation coefficient (ICC) with 95% confidence intervals [50]. ICC analysis was performed using a two-way mixed-effects, absolute-agreement, single-measurement model [50]. ICC values < 0.50 were interpreted as indicating poor reproducibility, 0.50–0.75 as moderate, 0.75–0.90 as good, and >0.90 as excellent reproducibility [50].

Spatial overlap between the final EPVS segmentations generated by the two observers was additionally assessed using the Dice similarity coefficient (DSC) [51]. DSC analysis was performed separately for the final whole-brain EPVS masks and for the bilateral BG-EPVS and bilateral CSO-EPVS masks to assess regional spatial agreement. This additional analysis enabled interobserver reproducibility of EPVS segmentation to be evaluated in terms of both volumetric agreement and spatial overlap at the whole-brain, BG, and CSO levels.

### 2.9. Statistical Analysis

All statistical analyses and sample size calculations were performed using IBM SPSS Statistics for Windows (version 29.0; IBM Corp., Armonk, NY, USA) and R (version 4.3.3; R Foundation for Statistical Computing, Vienna, Austria). Before cohort assembly and analysis of the study outcomes, a sample-size calculation was performed to estimate the number of evaluable participants required to detect a small-to-moderate standardized between-group difference. An investigator-specified Cohen’s d of 0.42 was used for this calculation; this value was not derived from the observed results of the present cohort. With a two-sided α of 0.05 and 80% statistical power (1 − β = 0.80), the calculation indicated a minimum requirement of approximately 90 evaluable participants per group. Accordingly, the minimum target sample size was set at 180 participants. The final study cohort comprised 123 patients with epilepsy and 108 controls, thereby exceeding the minimum required sample size in both groups.

Statistical analyses were conducted using a hierarchical approach. In the first stage, quantitative imaging measures constituting the EPVS, CP, and ventricular CSF phenotypes were compared between the epilepsy and control groups. Clinical association analyses were subsequently restricted to imaging measures that showed statistically significant between-group differences after false discovery rate (FDR) correction and were performed exclusively within the epilepsy group. Thus, the clinical association analyses were conducted within a hierarchical analytical framework designed to evaluate the clinical correlates of quantitative imaging phenotypes supported by group-level differences.

Continuous variables were summarized as mean ± standard deviation or median and interquartile range (IQR), as appropriate to their distributions, whereas categorical variables were presented as counts and percentages. Between-group comparisons were performed for demographic variables applicable to both the epilepsy and control groups. Continuous variables were compared using the independent-samples *t* test or Welch *t* test, whereas categorical variables were compared using the Pearson χ^2^ test or Fisher exact test, as appropriate. No epilepsy–control group comparisons were performed for clinical or EEG characteristics specific to the epilepsy group.

To assess potential selection effects associated with cohort formation, available demographic characteristics were additionally compared between included participants and excluded candidates separately within the epilepsy and control groups. Age was compared using Welch’s *t* test, and sex distribution was compared using the Pearson χ^2^ test.

Between-group differences in quantitative imaging measures comprising the EPVS, CP, and ventricular CSF phenotypes were evaluated using general linear models, with group status entered as the primary independent variable. All models were adjusted for age and sex. For models evaluating absolute volumetric measures, eTIV was additionally included as a covariate to account for interindividual differences in intracranial volume. eTIV was not additionally included in models evaluating eTIV-normalized volumetric measures, asymmetry indices, or dimensionless relative distribution measures. Group-specific estimated marginal means (EMMs), adjusted between-group differences calculated as epilepsy minus control, and corresponding 95% confidence intervals were derived from the adjusted models. Two-sided unadjusted *p* values for the group effect were reported. To account for multiple comparisons, the EPVS, CP, and ventricular CSF phenotypes were treated as three separate imaging hypothesis families. Unadjusted *p* values from the quantitative comparisons within each imaging family were subjected to FDR correction using the Benjamini–Hochberg procedure within the corresponding family. FDR correction was performed independently for the EPVS, CP, and ventricular CSF imaging families. Within the EPVS imaging family, all quantitative phenotype comparisons, including global, hemispheric, regional, asymmetry, relative-distribution, and ratio-derived measures, were included within the same FDR correction framework. Both unadjusted *p* values and corresponding FDR-adjusted *q* values were reported, with *q* < 0.05 considered statistically significant.

Model assumptions were systematically evaluated for all EPVS outcomes included in the between-group analyses, irrespective of statistical significance. Residual-versus-fitted plots and normal Q–Q plots were visually inspected for systematic residual-pattern and distributional departures, heteroscedasticity was assessed using the Breusch–Pagan test, and potentially influential observations were evaluated jointly using studentized residuals, leverage, and Cook’s distance. Multicollinearity was assessed using variance inflation factors (VIFs). Diagnostic findings were used to identify observations and models requiring further examination rather than as automatic exclusion criteria.

To assess the robustness of model-based inference, HC3 heteroscedasticity-consistent inference was evaluated uniformly across all EPVS between-group models. Participant-level nonparametric bootstrap inference with 5000 resamples and bias-corrected and accelerated (BCa) 95% confidence intervals was additionally used to assess the stability of the adjusted group contrasts. For outcomes showing the clearest departures from conventional model assumptions, transformation-based sensitivity analyses were additionally performed; specifically, right and left CSO-to-BG ratios were evaluated after logarithmic transformation.

Adjusted standardized between-group effect sizes (d_adj) were calculated for all EPVS comparisons as the model-adjusted epilepsy-minus-control contrast divided by the residual standard deviation of the corresponding model. The 95% confidence limits for d_adj were obtained by standardizing the lower and upper confidence limits of the corresponding adjusted group contrast using the residual standard deviation from the same model.

To characterize the extent of expert modification of the SHIVA-PVS outputs and assess whether the correction burden differed between groups, the initial automated and final corrected EPVS masks were additionally compared. Correction frequency was defined as the presence of any voxel-level modification between the initial and final masks. Correction magnitude was quantified as the voxelwise symmetric difference between the initial automated mask (A) and final corrected mask (C), normalized to the initial automated mask volume and expressed as a percentage [100 × |A Δ C|/|A|], thereby accounting for both added and removed voxels. Correction frequency was compared between the epilepsy and control groups using the Pearson chi-square test, whereas correction magnitude was compared using the Mann–Whitney U test.

Agreement between the initial automated and final corrected masks was additionally assessed to characterize the extent of modification introduced during expert quality control. Volumetric agreement was evaluated using a two-way mixed-effects, absolute-agreement, single-measure intraclass correlation coefficient (ICC) with 95% confidence intervals, and voxelwise spatial overlap was assessed using the Dice similarity coefficient (DSC) for whole-brain, bilateral CSO, and bilateral BG EPVS masks. These analyses were used to characterize automated-to-corrected mask agreement and were not considered estimates of segmentation accuracy against an independent reference standard. As a sensitivity analysis, the primary EPVS between-group analyses were repeated using the initial unedited SHIVA-PVS masks to assess whether the principal findings were sensitive to the expert manual-correction step. The same statistical models, covariate structure, and multiple-comparison framework used for the corresponding primary EPVS analyses were retained. A separate threshold-sensitivity analysis was performed using the retained SHIVA-PVS probability maps. The corresponding EPVS analyses were repeated after binarization at probability thresholds of 0.40 and 0.60, representing more permissive and more conservative alternatives, respectively, symmetrically positioned around the primary threshold of 0.50. The same statistical models, covariate structure, and FDR correction framework used for the corresponding primary analyses were applied at each alternative threshold.

Clinical association analyses were restricted to EPVS measures that showed significant between-group differences after FDR correction and were performed exclusively within the epilepsy group. These individual adjusted clinical-association analyses were considered exploratory and were not intended to estimate independent effects of mathematically or clinically interrelated clinical characteristics. Adjusted associations of age at epilepsy onset, disease duration, and the number of ASMs used at the time of MRI examination with FDR-significant EPVS measures were examined using multivariable linear regression models, with each clinical variable evaluated as a separate explanatory variable. All models were adjusted for age and sex; eTIV was additionally included as a covariate when the outcome was an absolute EPVS volume. eTIV was not additionally included in models of relative EPVS burden or CSO-to-BG ratios. Effects of continuous clinical variables were reported as unstandardized regression coefficients (*B*), representing the expected change in the corresponding EPVS measure per one-unit increase in the clinical variable, together with 95% confidence intervals.

To characterize the interrelationships among the major clinical predictors, pairwise associations were assessed using Spearman rank correlation coefficients within the epilepsy group. Collinearity was additionally evaluated using tolerance and variance inflation factor (VIF) values for a temporal predictor set comprising age at MRI, age at epilepsy onset, and disease duration and for a treatment-related predictor set comprising the number of ASMs at MRI, treatment regimen, and drug resistance. The temporal predictor set was evaluated for diagnostic purposes to quantify the collinearity arising from the mathematical relationship among age at MRI, age at epilepsy onset, and disease duration and was not used as an inferential model to estimate their simultaneous clinical effects.

A limited set of exploratory sensitivity analyses was performed to assess the direction, magnitude, and stability of the principal clinical associations under alternative adjustment for major related clinical predictors. These analyses focused on total EPVS volume as the principal measure of global EPVS burden and total BG-EPVS volume as the bilateral aggregate measure of the regional phenotype; right and left BG-EPVS volumes were additionally evaluated for disease duration to assess directional consistency across hemispheres. For disease duration, models were repeated using an alternative specification in which age at MRI was not included as a covariate, while sex and, for absolute-volume outcomes, eTIV were retained. For treatment-related characteristics, limited mutual-adjustment models were used to evaluate the stability of associations involving drug resistance, the number of ASMs, and treatment regimen after accounting for closely related treatment characteristics. These sensitivity analyses were exploratory and were not intended to estimate independent causal or clinical effects.

Associations of binary clinical characteristics, including drug resistance and treatment regimen (monotherapy versus polytherapy), with FDR-significant EPVS measures were evaluated using general linear models. Models were adjusted for age and sex and, when absolute EPVS volume was the outcome, additionally for eTIV. Adjusted between-group differences were reported with corresponding 95% confidence intervals. For the drug-resistance analysis, positive differences were defined as indicating higher EPVS values in patients with drug-resistant epilepsy, whereas for the treatment-regimen analysis, positive differences indicated higher EPVS values in the polytherapy group than in the monotherapy group.

Associations of multicategorical clinical variables, including epilepsy type and EEG characteristics, with FDR-significant EPVS measures were evaluated using covariate-adjusted general linear models. All models were adjusted for age and sex and, when absolute EPVS volume was the outcome, additionally for eTIV. Category-specific results were derived as adjusted EMMs with 95% confidence intervals, and the overall effect of each categorical variable on the corresponding EPVS measure was evaluated using an omnibus *F* test. Analyses of EEG lateralization and EEG anatomical localization were restricted to participants in whom the relevant characteristic could be defined and for whom category sizes were sufficient for formal statistical analysis. Category-specific EMMs were not interpreted as pairwise group differences when the corresponding omnibus effect did not remain significant after FDR correction.

The within-epilepsy clinical-association analyses were exploratory and were restricted to EPVS measures that showed FDR-significant epilepsy–control differences in the preceding between-group analysis. Accordingly, entry of EPVS outcomes into the clinical-association stage was data-dependent and constituted a post-selection analytical step. The two-sided raw *p* values from these analyses were subjected to Benjamini–Hochberg FDR correction within a single exploratory clinical-association family comprising 64 inferential tests. This family consisted of 30 tests for continuous clinical predictors, 20 tests for binary clinical predictors, and 14 omnibus tests for multicategorical clinical and EEG predictors. Specifically, the FDR family included the *p* values for the corresponding clinical-variable coefficients from adjusted linear regression models for continuous clinical variables, the *p* values for the group effects from adjusted general linear models for binary clinical variables, and the *p* values from omnibus F tests for multicategorical clinical variables. Category-specific estimated marginal means did not constitute additional tests in this FDR family. Both raw *p* values and corresponding FDR-adjusted *q* values were reported, with *q* < 0.05 considered statistically significant. For multicategorical variables, post hoc pairwise comparisons based on estimated marginal means were performed only when the corresponding omnibus effect remained significant after FDR correction; *p* values for these comparisons were adjusted separately using the Tukey procedure and were not included in the 64-test Benjamini–Hochberg family.

To evaluate the influence of the data-dependent EPVS outcome-selection step, an additional exploratory sensitivity analysis removed the requirement for a preceding FDR-significant epilepsy–control difference and evaluated the corresponding clinical associations across an expanded 18-measure EPVS outcome set while otherwise retaining the predictor-specific analytical architecture of the primary clinical analyses. This expanded sensitivity-analysis FDR family comprised 114 inferential tests: 54 tests for continuous clinical predictors, 36 tests for binary clinical predictors, and 24 omnibus tests for multicategorical clinical and EEG predictors. The 114 raw *p* values were adjusted jointly using the Benjamini–Hochberg procedure.

For the interobserver reproducibility analysis, agreement between EPVS, CP, and ventricular CSF volumetric measurements obtained independently by the two observers was assessed using a two-way mixed-effects, absolute-agreement, single-measurement ICC with 95% confidence intervals. For EPVS, total, right and left hemispheric, right and left BG-EPVS, and right and left CSO-EPVS volumes were evaluated. For CP, right, left, and total volumes were assessed. For ventricular CSF, right, left, and total lateral ventricular volumes; right, left, and total inferior lateral ventricular volumes; third and fourth ventricular volumes; and total ventricular CSF volume were evaluated. Interobserver spatial overlap of the final EPVS segmentations was additionally assessed using the DSC separately for the whole-brain, bilateral BG-EPVS, and bilateral CSO-EPVS masks. DSC values were reported as continuous similarity measures.

No imputation was performed for missing data, and each analysis was conducted using participants with complete data for the variables required for the corresponding model or statistical test. Derived ratio or asymmetry measures that could not be calculated because of a zero denominator were treated as missing values; no constant was added and no imputation was performed for these values. All statistical tests were two-sided. Statistical significance was defined as *q* < 0.05 for analyses subjected to FDR correction and *p* < 0.05 for analyses not subjected to FDR correction.

## 3. Results

### 3.1. Demographic and Clinical Characteristics

No significant differences were observed between the epilepsy and control groups in age at MRI examination, sex distribution, or eTIV (*p* = 0.10, *p* = 0.69, and *p* = 0.13, respectively). In the epilepsy group, focal epilepsy was the most common epilepsy type, and focal epileptiform activity was the most frequent EEG finding. Detailed clinical characteristics and EEG findings of the epilepsy group are presented in Table 4. MRI examinations from both groups were distributed throughout the study period. The numbers of epilepsy/control examinations by acquisition year were 10/8 in 2018, 13/11 in 2019, 14/12 in 2020, 11/10 in 2021, 16/14 in 2022, 17/15 in 2023, 21/19 in 2024, 15/14 in 2025, and 6/5 from January through March 2026, respectively. Although the number of examinations varied across calendar years, the descriptive temporal pattern was similar between the groups, without marked differential concentration of either group in the earlier or later portion of the study period. Available demographic characteristics were also compared between included participants and excluded candidates within each diagnostic group. In the epilepsy group, included participants were modestly older than excluded candidates (14.4 ± 2.2 vs. 13.7 ± 1.6 years, respectively; *p* = 0.004), whereas sex distribution did not differ significantly (female, 49.6% vs. 40.7%, respectively; *p* = 0.149). Similarly, included controls were modestly older than excluded control candidates (13.9 ± 2.3 vs. 13.1 ± 1.9 years, respectively; *p* = 0.003), while sex distribution did not differ significantly (female, 47.2% vs. 42.3%, respectively; *p* = 0.427).

### 3.2. Enlarged Perivascular Space (EPVS) Phenotype Analysis

Global EPVS burden was significantly higher in the epilepsy group than in the control group, and this difference remained significant in both hemispheres (all FDR-adjusted *q* < 0.01). Regional analyses showed the most pronounced differences in the BG, with significantly higher bilateral BG-EPVS burden in the epilepsy group (all *q* < 0.001), whereas no significant between-group differences were observed in absolute CSO-EPVS burden or EPVS asymmetry indices (all *q* > 0.05). Significant between-group differences were also observed in relative regional EPVS burden and CSO-to-BG distribution ratios; the epilepsy group exhibited a higher relative BG burden, together with a lower relative CSO burden and lower bilateral CSO-to-BG ratios (all *q* < 0.01). Detailed results of the EPVS phenotype analysis are presented in Table 5. Any voxel-level modification between the initial automated and final corrected SHIVA-PVS masks was observed in 68/123 (55.3%) participants with epilepsy and 56/108 (51.9%) controls, with no significant between-group difference in correction frequency (*p* = 0.60). The median correction magnitude was 5.2% (IQR, 3.1–7.6%) in the epilepsy group and 4.9% (IQR, 2.9–7.1%) in controls, with no significant between-group difference (*p* = 0.45). Volumetric agreement between the initial automated and final corrected masks was high (ICC = 0.91; 95% CI, 0.89–0.93). Spatial overlap between the initial automated and final corrected masks yielded DSC values of 0.82 for whole-brain EPVS, 0.78 for bilateral CSO-EPVS, and 0.72 for bilateral BG-EPVS. Detailed correction-burden and automated-to-corrected mask agreement results are provided in Supplementary Table S1. When the primary EPVS analyses were repeated using the initial unedited SHIVA-PVS masks, the principal between-group pattern was preserved. Total EPVS burden remained higher in the epilepsy group, with an adjusted between-group difference of 240 mm^3^ (*p* = 0.0041). The strongest regional difference remained localized to the BG (*p* ≤ 0.001), whereas absolute CSO-EPVS measures remained nonsignificant after FDR correction (*p*/*q* > 0.05). The directions of the principal EPVS findings were consistent with those obtained using the final corrected masks. Detailed results of the corrected- and unedited-mask analyses are provided in Supplementary Table S2. The principal EPVS findings were also preserved across the alternative probability thresholds. For total EPVS burden, the adjusted epilepsy–control difference was 306 mm^3^ at a threshold of 0.40 (*p* = 0.0014, *q* = 0.004), 284.7 mm^3^ at the primary threshold of 0.50 (*p* = 0.001, *q* = 0.003), and 262 mm^3^ at a threshold of 0.60 (*p* = 0.0032, *q* = 0.007). The BG-predominant difference remained the strongest regional finding at both alternative thresholds, with right BG differences of approximately 89 mm^3^ at 0.40 and 78 mm^3^ at 0.60 (both *p* < 0.001). Absolute CSO-EPVS measures remained nonsignificant after FDR correction at both alternative thresholds (*p*/*q* > 0.05), as did the asymmetry measures. The directions of the principal relative-distribution findings were preserved, although the statistical strength of individual secondary relative measures varied across thresholds. Detailed threshold-sensitivity results are provided in Supplementary Table S3.

Model diagnostics and robustness analyses for the EPVS between-group comparisons are summarized in Supplementary Table S4. Multicollinearity was low across the fitted models (maximum VIF, 1.24), and no participant was excluded solely on the basis of model diagnostics. HC3 heteroscedasticity-consistent inference and participant-level BCa bootstrap analyses yielded conclusions concordant with the principal models, without altering the substantive pattern of FDR-supported versus unsupported findings. The right and left CSO-to-BG ratio models showed the clearest departures from conventional model assumptions; targeted analyses using log-transformed ratios yielded adjusted group effects of −0.48 (95% CI, −0.69 to −0.27; *p* < 0.001) and −0.47 (95% CI, −0.68 to −0.26; *p* < 0.001), respectively, consistent in direction and inference with the original-scale analyses.

Adjusted standardized effect sizes for all EPVS comparisons are provided in Supplementary Table S4. The adjusted standardized effect was 0.42 (95% CI, 0.16–0.68) for total EPVS volume and 0.77 (95% CI, 0.43–1.11) for total BG-EPVS volume.

### 3.3. Choroid Plexus (CP) Phenotype Analysis

No statistically significant differences were observed between the epilepsy and control groups in either absolute or eTIV-normalized CP volumes (all FDR-adjusted *q* > 0.05). Similarly, no significant between-group differences were found in CP asymmetry or in proportional CP measures defined relative to lateral ventricular and total ventricular CSF volumes (all *q* > 0.05). Detailed results of the CP phenotype analysis are presented in Table 6.

### 3.4. Ventricular Cerebrospinal Fluid (CSF) Phenotype Analysis

No statistically significant differences were observed between the epilepsy and control groups in either absolute or eTIV-normalized ventricular CSF volumes (all FDR-adjusted *q* > 0.05). Similarly, no significant between-group differences were found in the asymmetry indices of the lateral or inferior lateral ventricles (all *q* > 0.05). Detailed results of the ventricular CSF phenotype analysis are presented in Table 7.

### 3.5. Interobserver Reproducibility of Quantitative Imaging Measurements

Interobserver reproducibility of selected EPVS phenotype measures and CP and ventricular CSF volumetric measurements ranged overall from moderate to excellent, with good reproducibility observed for most measures. For EPVS phenotype measures, ICC values ranged from 0.71 to 0.87, with good agreement for total EPVS volume (ICC = 0.87; 95% CI, 0.77–0.93). Regional BG-EPVS reproducibility was lower than that observed for total EPVS volume, with ICCs of 0.71 (95% CI, 0.52–0.83), 0.74 (95% CI, 0.57–0.85), and 0.77 (95% CI, 0.61–0.86) for right, left, and total BG-EPVS volumes, respectively. Agreement for CP and ventricular CSF measurements was predominantly good and reached excellent levels for total CP, total lateral ventricular, and total ventricular CSF volumes (ICC ≥ 0.91). Detailed results of the interobserver reproducibility analysis for quantitative imaging measurements are presented in Table 8. DSC values assessing the spatial overlap of EPVS segmentations were 0.82 ± 0.06 for the whole-brain masks, 0.78 ± 0.07 for the bilateral CSO masks, and 0.72 ± 0.09 for the bilateral BG masks. The corresponding median (IQR) DSC values were 0.83 (0.78–0.86), 0.79 (0.73–0.83), and 0.73 (0.66–0.78), respectively.

### 3.6. Clinical Associations of FDR-Significant Quantitative MRI Phenotypes

In accordance with the prespecified hierarchical analytical approach, clinical association analyses were restricted to EPVS phenotype measures that showed significant differences between the epilepsy and control groups after FDR correction. Within the epilepsy group, longer disease duration was associated with greater global EPVS burden and, more prominently, with greater BG-EPVS burden; these associations also remained significant after FDR correction for relative regional EPVS burden and CSO-to-BG distribution measures (all *q* < 0.01).

The number of ASMs and polytherapy showed more limited but significant associations, particularly with BG-EPVS burden, whereas associations with age at epilepsy onset were weaker and more selective. Neither epilepsy type nor EEG classification showed a significant omnibus effect on FDR-significant EPVS measures (all *q* > 0.05). In contrast, EEG lateralization was selectively associated with BG-EPVS measures; omnibus effects that remained significant after FDR correction were associated with higher BG-EPVS burden in the bilateral group. No significant associations were observed between EEG anatomical localization and FDR-significant EPVS measures after FDR correction. Detailed results of the adjusted associations between continuous and binary clinical variables and FDR-significant EPVS measures are presented in Table 9. Detailed results of the adjusted associations of epilepsy type and EEG characteristics with FDR-significant EPVS measures are presented in Table 10. Post hoc pairwise comparisons for the FDR-significant effects of EEG lateralization are presented in Table 11.

Exploratory assessment of the major clinical predictors confirmed substantial interrelationships among the temporally and treatment-related variables (Supplementary Table S5). Among the temporal variables, age at MRI was correlated with age at epilepsy onset (ρ = 0.35, *p* < 0.001) and disease duration (ρ = 0.27, *p* = 0.003), whereas age at epilepsy onset was inversely correlated with disease duration (ρ = −0.76, *p* < 0.001). In the diagnostic temporal predictor set, VIF values were 7.1 for age at MRI, 22.2 for age at epilepsy onset, and 24.4 for disease duration, reflecting their mathematical interdependence. Among treatment-related variables, the number of ASMs was correlated with polytherapy (ρ = 0.86, *p* < 0.001) and drug resistance (ρ = 0.56, *p* < 0.001), and polytherapy was correlated with drug resistance (ρ = 0.49, *p* < 0.001); the corresponding VIF values were 3.7, 3.4, and 1.6, respectively.

In the exploratory sensitivity analyses, disease-duration estimates remained directionally consistent and of broadly similar magnitude when age at MRI was omitted from the adjustment set: the estimate for total EPVS volume changed from B = 48.7 (95% CI 25.1 to 72.3) to B = 42.1 (95% CI 17.0 to 67.2), and the estimate for total BG-EPVS volume changed from B = 15.5 (95% CI 10.2 to 20.8) to B = 18.4 (95% CI 8.1 to 28.7). Right and left BG-EPVS estimates showed the same direction of association (Supplementary Table S5). In the treatment-related sensitivity analyses, the drug-resistance estimates remained directionally consistent after additional adjustment for ASM number or polytherapy, and the ASM-number estimate for total BG-EPVS volume remained directionally consistent after adjustment for drug resistance. In contrast, the polytherapy estimate for total BG-EPVS volume was attenuated after adjustment for drug resistance (adjusted difference, 69; 95% CI −3 to 141; *p* = 0.060). These sensitivity analyses were exploratory and were used to assess model stability rather than to establish independent clinical effects.

In the expanded sensitivity analysis removing the preceding significance-based EPVS outcome-selection criterion, the corresponding clinical associations were evaluated across an expanded 18-measure EPVS outcome set, yielding a single FDR family of 114 inferential tests (Supplementary Table S7). Several of the stronger associations, particularly those involving disease duration and BG-related EPVS measures, remained FDR-significant under the expanded multiplicity structure, whereas some weaker associations were attenuated after adjustment across the larger FDR family. This analysis was conducted as an exploratory sensitivity analysis of the outcome-selection step and was not used to redefine the primary clinical-association analysis. The complete 64-test primary exploratory clinical-association matrix, including model-specific sample sizes, effect estimates with 95% confidence intervals, raw *p* values, and FDR-adjusted *q* values, is provided in Supplementary Table S6, whereas the corresponding expanded 114-test sensitivity analysis is reported in Supplementary Table S7. The composition of both FDR families and the ordered raw *p*-value inputs at full computational precision are provided in Supplementary Table S8, and Supplementary Material S4 provides statistical code for independent reproduction of the Benjamini–Hochberg adjustments.

## 4. Discussion

The increased global EPVS burden in pediatric epilepsy, accompanied by a regional distribution that was most pronounced in the BG, suggests that EPVS may represent an imaging phenotype associated with the chronic course and clinical burden of the disease. The prominent associations with longer disease duration and drug resistance, together with more limited associations with treatment burden and the absence of significant differences according to epilepsy type, suggest that this phenotype may be more appropriately interpreted in the context of disease chronicity and clinical burden rather than as a feature specific to a particular epilepsy subtype. The preferential localization of these differences to the BG further indicates that EPVS distribution is not anatomically homogeneous and that perivascular spaces in this region may exhibit differential susceptibility to epilepsy-related processes. In contrast, the absence of accompanying volumetric differences in the CP and ventricular CSF phenotypes suggests that the observed EPVS pattern is unlikely to simply reflect a generalized alteration in CSF compartments and may instead be considered within the context of a more regionally distributed process related to perivascular–interstitial fluid dynamics [23,44]. These findings are insufficient to support the interpretation of EPVS burden as a diagnostic or prognostic imaging biomarker. Nevertheless, its quantitative assessment from routine MRI data, together with its significant associations with disease duration and indicators of clinical burden, supports further investigation of EPVS as a potential imaging phenotype associated with the chronic course of pediatric epilepsy.

Although diffusion tensor imaging (DTI) findings related to glymphatic function in epilepsy have increased in recent years, the literature remains predominantly based on adult cohorts with focal and temporal lobe epilepsy [11,12,13,14,15,52,53]. In temporal lobe epilepsy, particularly in the presence of hippocampal sclerosis, lower DTI-ALPS indices have been reported, and subsequent studies have investigated the hemispheric distribution of these alterations, their postoperative evolution, and their associations with ASM response [11,12,13,14,15,53]. Although these findings support a potential relationship between epilepsy and glymphatic function, they are based on an indirect DTI-derived imaging marker and have yielded findings that are not entirely consistent with respect to clinical associations [11,12,13,14,15,52,53]. In pediatric epilepsy, a lower DTI-ALPS index has been associated with longer disease duration in childhood absence epilepsy, while lower bilateral DTI-ALPS indices accompanied by increased white matter free-water fraction have been reported in children without an identifiable epileptogenic lesion on imaging [19,20]. Nevertheless, studies directly quantifying EPVS burden and its anatomical distribution in this age group remain limited [19,20,54]. In this context, the large adult cohort reported by Sinclair et al. provides an important point of comparison: BG-EPVS burden was higher across different epilepsy subtypes than in controls, whereas white matter EPVS burden did not differ significantly [18]. The absence of substantial differences in EPVS burden across epilepsy subtypes further suggested that this alteration may not be specific to a particular epilepsy syndrome [18]. In contrast, the lack of a significant association between EPVS burden and disease duration in that study represents an important divergence from the clinical findings of the present study [18]. This discrepancy may be related to differences in age, clinical course, and cohort characteristics and underscores the importance of examining the pediatric period separately [18,19,20,21,55]. Within this context, by quantitatively assessing global and regional EPVS burden together with CP and ventricular CSF phenotypes within the same cohort, our study provides a complementary imaging perspective to the predominantly DTI-based pediatric literature, integrating the anatomical distribution of EPVS with the assessment of CSF compartments in pediatric epilepsy.

Regional differences in EPVS burden should be interpreted in the context of the distinct anatomical environments of the perivascular spaces within the BG and CSO. Perivascular spaces extend along penetrating cerebral vessels and are involved in fluid exchange and clearance processes between CSF and interstitial fluid; however, vascular architecture and perivascular organization are not uniform across brain regions [23,44]. Perivascular spaces in the BG are primarily associated with lenticulostriate and other deep penetrating arteries, whereas those in the CSO follow the course of medullary vessels within the subcortical white matter [56,57]. Differences in the organization of periarterial structures, the characteristics of the surrounding parenchyma, and the regional perivascular microenvironment further suggest that these two compartments may not represent entirely equivalent conditions with respect to perivascular–interstitial fluid dynamics [23,44,56,57,58]. Accordingly, BG-EPVS and CSO-EPVS may be considered not merely as manifestations of total EPVS burden in different anatomical regions, but as complementary measures that may be influenced to different degrees by regional characteristics [44,58]. This anatomical framework highlights the importance of considering the regional distribution of the increased global EPVS burden observed in our study. Although the difference between the epilepsy and control groups was pronounced for BG-EPVS burden, no comparable difference was observed in absolute CSO-EPVS burden, suggesting that total EPVS volume alone may not fully capture the topographic characteristics of EPVS burden. Thus, evaluating the BG and CSO separately, in addition to global burden, may provide additional information for characterizing the regional organization of the epilepsy-associated EPVS phenotype. A similar divergence between BG and white matter EPVS measures in adult epilepsy data further supports the relevance of investigating this regional pattern across different age groups [18]. Nevertheless, EPVS volume is not a direct measure of perivascular fluid dynamics or glymphatic function, and MRI-derived quantitative EPVS burden may be influenced by multiple interrelated biological processes [23,44,58]. Therefore, the biological processes underlying the observed regional differences cannot be determined from the present data. Our findings suggest that perivascular spaces within the BG and CSO may exhibit differential regional susceptibility to epilepsy-associated alterations.

The regional differences observed in the BG may be considered within the framework of the close relationship between the neurovascular unit and perivascular fluid homeostasis. Fluid movement surrounding penetrating arteries is related to the dynamic properties of the vessel wall and the perivascular interface formed by astrocytic endfeet, and human MRI studies further support a close relationship between arterial pulsatility and perivascular CSF movement [23,44,59]. Nevertheless, CSF–interstitial fluid exchange is a complex process involving multiple vascular and cellular factors, and arterial pulsatility should not be regarded as the sole determinant of this physiology [23,44]. The polarized distribution of AQP4 at astrocytic endfeet, an important component of the perivascular interface, is also closely related to brain water homeostasis and perivascular fluid exchange [3,4,60]. In epilepsy, reported alterations in blood–brain barrier integrity and the perivascular organization of AQP4, together with astrocytic reactivity and neuroinflammatory responses that may accompany seizure activity, provide a biological framework supporting the presence of epilepsy-associated alterations within the neurovascular and perivascular microenvironment [1,2,3,4,60,61]. The relationships among these processes are complex, however, and the extent to which they are reflected in MRI-derived quantitative EPVS burden remains unclear [3,4,23]. Given the distinctive vascular and perivascular characteristics of the deep penetrating vascular bed, it is biologically plausible that vascular, astrocytic, and inflammatory alterations associated with epilepsy may be manifested differently within the perivascular microenvironment of the BG than in other anatomical regions; however, the present data do not allow this possibility to be confirmed or attributed to a specific mechanism [3,4,23,44]. Therefore, the BG-predominant EPVS finding should not be interpreted as a direct marker of glymphatic dysfunction or of a specific pathophysiological mechanism, but rather as an imaging finding consistent with the possibility that epilepsy-associated neurovascular and perivascular alterations may differ across anatomical regions.

These findings should be interpreted within the multidimensional EPVS phenotype framework used in this study. Total EPVS volume served as the principal measure of global burden, whereas regional, lateralized, asymmetry, relative-distribution, and ratio-derived measures characterized complementary aspects of anatomical distribution rather than statistically independent co-primary outcomes. While absolute EPVS volume reflects the magnitude of EPVS burden, relative regional measures provide complementary information regarding the anatomical distribution of this burden between the BG and CSO. The importance of regional EPVS assessment has also been emphasized in previous large-cohort studies. Evans et al. reported that EPVS burden across different anatomical regions may be associated with partly distinct determinants, highlighting the importance of evaluating the BG and CSO separately [45]. Similarly, analyses from the Framingham Heart Study considered BG, CSO, and mixed BG–CSO EPVS burdens separately, allowing the regional characteristics of the EPVS phenotype to be evaluated beyond total burden alone [62,63]. More recently, Yu et al. directly compared BG-EPVS and CSO-EPVS burdens and defined BG-predominant, CSO-predominant, and similarly distributed BG–CSO patterns as distinct phenotypes, thereby directly assessing the relative distribution of EPVS burden between these regions [64]. Within a similar conceptual framework, relative BG burden and relative CSO burden in our study quantify the contribution of each region to total EPVS burden, whereas the CSO/BG ratio characterizes the distribution of BG-EPVS and CSO-EPVS burdens relative to one another. Rather than simply restating differences in absolute volumes, these measures characterize how total EPVS burden is proportionally distributed between the BG and CSO. However, because they are mathematically derived from the same underlying BG-EPVS and CSO-EPVS volumes, they should not be regarded as measures independent of regional EPVS volumes or as independent confirmation of these findings. Accordingly, differences in these relative measures should not be interpreted as indicators of distinct biological processes, but rather as complementary features characterizing the relative distribution of the EPVS phenotype between the BG and CSO.

The position of our EPVS findings within the imaging literature on the glymphatic system should be considered in light of the fact that different MRI markers do not measure the same biological features. A substantial proportion of the existing epilepsy literature has relied on the DTI-ALPS approach, with studies reporting alterations related to glymphatic function across different adult and pediatric cohorts [11,13,14,19,20,52,53,54,55,65,66]. However, no direct equivalence can be established between these findings and the EPVS measures evaluated in our study. Whereas DTI-ALPS is an indirect index derived from water diffusion along the perivascular direction, EPVS burden represents a structural imaging phenotype characterizing the volumetric features of visibly enlarged perivascular spaces on MRI [22,23,44]. Thus, although both approaches may provide indirect information about processes related to perivascular fluid physiology, they should not be regarded as different measures of the same biological property. This distinction also limits the extent to which alterations observed across different MRI markers can be directly attributed to a common glymphatic mechanism. Our findings should therefore not be interpreted as an independent confirmation of the existing DTI-ALPS literature, but rather as complementary evidence regarding structural imaging features that may be related to perivascular physiology in epilepsy. Joint evaluation of EPVS burden, DTI-ALPS, and other MRI markers related to perivascular fluid physiology within the same cohorts may help clarify the relationships among these measures and determine the extent to which they reflect shared or distinct aspects of perivascular physiology. Accordingly, the principal contribution of the present study lies in the quantitative characterization of regional EPVS burden and distribution in pediatric epilepsy, particularly the BG-predominant pattern; the present structural MRI findings do not establish an underlying glymphatic mechanism.

The evaluation of the EPVS phenotype alongside CP and ventricular CSF measures enables epilepsy-associated imaging findings to be considered in the context of different CSF-related anatomical structures and compartments. The CP is the principal anatomical site of CSF production and a key component of the blood–CSF barrier, and its structural characteristics are increasingly being investigated across a range of neurological disorders [24,46,67]. Evidence in epilepsy, however, remains limited. Li et al. reported increased CP volume in temporal lobe epilepsy and associated greater CP volume with cognitive impairment and brain atrophy [17]. Nevertheless, the available evidence remains insufficient to determine whether structural CP differences represent a consistent feature across different age groups and epilepsy phenotypes. Ventricular CSF constitutes a macroscopic CSF compartment that is anatomically distinct from the perivascular spaces, and its volumetric characteristics are related not only to CSF physiology but also to ventricular anatomy, the volume of the surrounding brain parenchyma, and age-related developmental characteristics [28,29,44]. In the pediatric epilepsy literature, Jackson et al. reported differences in ventricular volumes in new-onset epilepsy, particularly in patients with idiopathic generalized epilepsy [47]; however, the extent to which these findings can be generalized across different epilepsy phenotypes and throughout the course of the disease remains uncertain. Within this framework, statistically detectable between-group differences were observed in the EPVS phenotype, whereas no statistically detectable between-group differences were observed in the CP or ventricular CSF measures examined. These null findings should not be interpreted as evidence of equivalence or as demonstrating the absence of structural differences in these compartments. For example, the adjusted between-group difference in total CP volume was 54.5 mm^3^ (95% CI, −56.1 to 165.1), and that in total lateral ventricular volume was 332.4 mm^3^ (95% CI, −195.1 to 859.9), indicating that the data remain compatible with a range of between-group differences in both directions despite the absence of statistical significance. Thus, although the observed statistical pattern differed across the EPVS, CP, and ventricular CSF measures examined, it does not establish equivalence for the latter measures or exclude more generalized CSF-related structural alterations. Moreover, the absence of statistically detectable differences in volumetric CP and ventricular CSF measures should not be interpreted as evidence that CSF production, circulation, or clearance is preserved, because these structural imaging measures do not directly assess such physiological processes [22,23,26]. Accordingly, the CP and ventricular CSF findings should be viewed not as evidence of normal CSF physiology, but as complementary structural imaging findings considered alongside the EPVS phenotype.

The findings related to disease duration warrant further consideration of the relationship between the EPVS phenotype and the chronic course of epilepsy. Because longer disease duration reflects a longer period of living with epilepsy, these findings suggest that perivascular imaging features may be associated not only with the presence of the disease but also with its course over time [3,4,65,66]. The MRI literature on glymphatic-related alterations in epilepsy provides a relevant point of comparison in this regard [3,4]. DTI-ALPS studies across different epilepsy populations have reported associations between longer disease duration and lower index values [13,53], and recent meta-analyses have shown that the inverse association between disease duration and DTI-ALPS is generally maintained across studies [65,66]. Although no direct biological equivalence can be established between these studies and our EPVS findings, the observation of disease duration-related associations across different imaging approaches supports further investigation of the relationship between the chronic course of epilepsy and MRI features related to perivascular physiology [22,23,65,66]. However, the clinical meaning of disease duration cannot be considered independently of age and age at seizure onset. Because these variables are interrelated, a longer disease duration at a given age may also reflect an earlier seizure onset [65,66]. Accordingly, the observed associations with age at epilepsy onset and disease duration should be regarded as exploratory temporal associations and not as independent effects separable from their mathematical relationship with age at MRI. This distinction is particularly important in pediatric populations, because the duration of exposure to epilepsy may span different developmental periods, making it difficult to attribute the observed associations solely to chronological disease duration [21,28,29]. Therefore, although our findings suggest that the EPVS phenotype may be associated with the chronic course of epilepsy, they do not demonstrate that EPVS burden increases over time or represents a progressive alteration [65,66]. Determining the temporal nature of this association will require longitudinal studies evaluating within-individual changes in the EPVS phenotype in conjunction with the clinical course of epilepsy [65].

The findings related to drug resistance, number of ASMs, and polytherapy should not be interpreted as independent clinical effects but rather considered together in the context of the close relationship between difficulty in achieving seizure control and treatment intensity [31,68]. The exploratory sensitivity analyses were consistent with this interpretation: the direction of the drug-resistance and ASM-number associations was generally preserved under limited mutual adjustment, whereas the polytherapy estimate was attenuated after adjustment for drug resistance, further underscoring the clinical overlap among these treatment-related characteristics. The within-epilepsy clinical-association analyses should additionally be interpreted as exploratory and post-selection because entry of EPVS outcomes into the primary clinical-association stage depended on FDR-significant differences in the preceding epilepsy–control comparison. Consequently, the FDR-adjusted *q* values from the primary clinical-association family account for multiplicity among the second-stage clinical tests but do not incorporate uncertainty introduced by the preceding data-dependent outcome-selection step. The expanded sensitivity analysis removed this significance-based outcome-selection criterion and evaluated the corresponding associations across a broader 18-measure EPVS outcome set; however, this analysis remains exploratory and does not convert the observed clinical associations into confirmatory evidence. Drug resistance is defined by the failure to achieve sustained seizure freedom after adequate trials of two appropriately chosen and tolerated ASM regimens, and greater difficulty in achieving seizure control may be accompanied in clinical practice by the use of a greater number of ASMs and polytherapy [31,68]. Accordingly, the number of ASMs and polytherapy should be interpreted not solely as pharmacological treatment characteristics but in conjunction with features related to the clinical course of epilepsy and seizure control [31,68]. The imaging literature on glymphatic-related alterations in epilepsy provides a similar clinical context with respect to treatment response [3,4,13,55]. Kim et al. associated lower DTI-ALPS indices with poorer ASM response in focal epilepsy, whereas Jin et al. reported differences in DTI-ALPS measures according to ASM response in focal cortical dysplasia-related epilepsy [13,55]. Although these studies suggest a potential association between glymphatic-related MRI features and treatment response, they do not establish whether the observed imaging differences are related to treatment itself or to difficulty in achieving seizure control and the accompanying clinical characteristics [13,55]. Within this framework, the findings associated with drug resistance, number of ASMs, and polytherapy in our study are more appropriately interpreted collectively, given that these clinically interrelated variables showed associations with the same perivascular phenotype [31,68]. In particular, the associations with the number of ASMs and polytherapy should not be attributed directly to pharmacological effects, because greater treatment intensity may covary with clinical characteristics such as difficulty in achieving seizure control and disease duration [31,68]. Consequently, the present cross-sectional data do not allow the relative contributions of epilepsy severity and treatment-related characteristics to the observed associations with the EPVS phenotype to be disentangled. Clarifying this distinction will require longitudinal studies examining the temporal relationships between changes in seizure control and treatment and the EPVS phenotype.

The EEG-related findings do not support interpreting the EPVS phenotype as a lateralized imaging feature that directly corresponds to the hemispheric direction of epileptiform activity. Although no clear distinction was observed between right- and left-lateralized activity, higher BG-EPVS measures in the group with bilateral epileptiform activity suggest that the observed association may be more appropriately considered in the context of broader electrophysiological involvement rather than a specific hemispheric orientation. However, because bilateral epileptiform activity does not directly indicate the anatomical extent of epileptiform discharges within the brain, this finding should not be interpreted as a direct imaging correlate of widespread electrophysiological involvement [69,70]. The potential for epileptiform activity to propagate beyond its site of onset and manifest bilaterally further indicates that the relationship between EEG lateralization and anatomical extent is not straightforward [69,70,71]. The absence of significant associations with epilepsy type or EEG anatomical localization further supports caution in interpreting the EPVS phenotype as an imaging feature specific to a particular epilepsy classification or anatomical focus. Taken together, the anatomical specificity of the association observed with EEG lateralization cannot be determined from the present data and warrants further investigation using more detailed electrophysiological assessments.

When the imaging phenotypes evaluated in our study are considered together, EPVS burden and its regional distribution between the BG and CSO, particularly in light of their associations with clinical characteristics, appear to constitute a perivascular imaging profile in pediatric epilepsy that is distinct from the CP and ventricular CSF phenotypes. This divergence suggests that different CSF-related anatomical structures and compartments do not necessarily undergo parallel alterations in the context of epilepsy and that the observed imaging features should not be subsumed under a single generalized morphometric alteration [17,18,44,47]. Within this framework, the BG-predominant regional perivascular phenotype may be considered a distinct imaging feature that characterizes both the magnitude and anatomical distribution of EPVS burden within its clinical context and differentiates it from the other CSF-related imaging phenotypes examined. Nevertheless, this phenotypic divergence does not imply biological independence among these structures and compartments or indicate a specific disturbance of fluid physiology; rather, it suggests that the CSF-related structures and compartments examined may not exhibit epilepsy-associated alterations to the same extent [17,18,23,44,47].

The methodological design of this study placed particular emphasis on limiting sources of clinical, technical, and analytical variability that could affect the interpretability of the imaging phenotypes. The relatively narrow pediatric age range and exclusion of epileptogenic structural lesions helped limit the potential effects of age-related developmental variability and overt structural abnormalities on imaging measurements, while acquisition of all imaging examinations on the same system reduced technical variability related to data acquisition [28,29]. For EPVS assessment, the use of multimodal imaging information and expert visual quality control of automated segmentations ensured that phenotype characterization did not rely solely on algorithmic output, while the additional assessment of interobserver reproducibility enabled direct evaluation of measurement consistency [35,38,39,40,50]. However, interobserver reproducibility was comparatively lower for regional BG-EPVS measurements than for total EPVS volume, and this greater regional measurement uncertainty should be considered when interpreting BG-specific quantitative findings. At the statistical level, the hierarchical analytical framework and control of multiple comparisons using FDR helped limit false discoveries in a study involving numerous interrelated imaging measures. In particular, restricting the clinical analyses to phenotypes that remained significant after FDR correction in the epilepsy–control comparisons allowed clinical associations to be examined specifically for phenotypes supported at the group level, rather than through broad post hoc screening across all imaging variables. This approach enabled technical measurement consistency and analytical caution to be considered jointly throughout the process, from characterization of the imaging phenotypes to evaluation of their clinical associations, thereby facilitating interpretation of the findings within a more controlled analytical framework.

Our findings should be interpreted in light of several limitations. First, the retrospective and cross-sectional design precludes determination of the temporal nature of the associations between the EPVS phenotype and clinical characteristics and does not allow within-individual changes in the EPVS phenotype to be tracked over time. The study was designed for cross-sectional quantitative imaging phenotyping and did not evaluate diagnostic accuracy, prognostic performance, treatment response, or clinical decision support. The reliance on available medical records for clinical data also limited the standardized assessment of clinical characteristics such as seizure frequency, recent seizure activity, cumulative seizure burden over time, and detailed treatment history. In particular, the exact interval between the most recent seizure and MRI acquisition could not be reliably reconstructed for every participant, precluding definition of a standardized minimum seizure-free interval. Although no participant included in the final epilepsy cohort demonstrated MRI evidence of cytotoxic or vasogenic edema on the examination used for quantitative analysis, the absence of visible edema cannot exclude more subtle or transient postictal alterations in perivascular or interstitial fluid dynamics. Accordingly, residual confounding related to recent seizure activity cannot be completely excluded. In addition, the close interrelationships among age, age at seizure onset, and disease duration, as well as the clinical interdependence of drug resistance, number of ASMs, and polytherapy, make it difficult to interpret their associations with the EPVS phenotype as independent effects. Potential contributions from other clinical and individual characteristics, including BMI/body size, blood pressure, sleep-related factors, non-antiseizure medication use, and recent acute illness, could not be systematically evaluated because of the retrospective data structure and therefore remain potential sources of residual confounding. Although predefined eligibility criteria excluded documented neurological and systemic disorders considered relevant to the quantitative MRI phenotypes, unrecognized, incompletely documented, or unmeasured conditions cannot be excluded in this retrospective clinical control cohort. In addition, the modest age differences observed between included and excluded candidates in both diagnostic groups indicate that potential selection effects associated with retrospective cohort formation cannot be completely excluded. In addition, although no control participant had a documented diagnosis of chronic tension-type headache or a vestibular disorder in the available clinical records, the retrospective absence of such diagnoses cannot be considered equivalent to their definitive exclusion. Because the control cohort was characterized using routine clinical records rather than a prospective, study-specific diagnostic assessment, unrecognized or undocumented headache or vestibular disorders cannot be completely excluded; therefore, their potential contribution to residual confounding in the between-group comparisons cannot be entirely ruled out. The evaluation of EEG findings using categorical classifications derived from clinical reports, together with the restriction of lateralization and anatomical localization analyses to smaller patient subgroups, represents an additional limitation to a more detailed and quantitative assessment of associations with electrophysiological characteristics; accordingly, these subgroup findings should be interpreted cautiously. Finally, the within-epilepsy clinical-association analyses were exploratory and post-selection, and although the expanded sensitivity analysis removed the preceding significance-based outcome-selection criterion, these findings require independent replication. Although all MRI examinations underwent neuroradiological review and participants with identifiable structural lesions were excluded according to the predefined eligibility criteria, the retrospectively available clinical imaging protocol was not fully equivalent to a contemporary dedicated high-resolution epilepsy MRI protocol, particularly with respect to the 5-mm conventional axial T2-weighted acquisition. Therefore, the possibility that subtle epileptogenic structural abnormalities below the detection capability of the available clinical acquisitions remained undetected cannot be completely excluded. From an imaging perspective, although automated EPVS segmentation was supported by multimodal imaging information, expert visual quality control, assessment of interobserver reproducibility, and additional sensitivity analyses, the pretrained model used in this study was not independently validated against a manually delineated reference standard generated without access to the SHIVA-PVS outputs in this pediatric cohort. Accordingly, the interobserver reproducibility, automated-to-corrected mask agreement, correction-burden, and sensitivity analyses support measurement consistency and robustness to the evaluated processing choices but do not establish cohort-specific segmentation accuracy. In addition, the comparatively lower interobserver reproducibility of regional BG-EPVS measurements should be considered when interpreting findings based on this regional phenotype. Independent validation against pediatric manual reference segmentations therefore remains necessary. Restricting the regional EPVS assessment to the BG and CSO also precluded evaluation of potential differences in other perivascular locations, particularly the temporal and hippocampal regions. The CP and ventricular CSF phenotypes were based on structural MRI measurements and therefore do not permit direct inferences regarding physiological processes such as CSF production, circulation, or clearance. Although interobserver reproducibility was generally good to excellent for CP and ventricular CSF volumetric measures, residual segmentation and measurement error cannot be excluded and could have reduced sensitivity to between-group differences. Furthermore, because extra-axial CSF was not quantified as a separate imaging phenotype, the relationship between the EPVS findings and CSF compartments outside the ventricular system could not be assessed. Similarly, EPVS burden should be recognized as a structural MRI phenotype rather than a direct measure of perivascular fluid physiology or glymphatic function. Finally, although examining a selected pediatric cohort within a relatively narrow age range, without epileptogenic structural lesions, at a single center using the same imaging system reduced developmental and technical variability, these features may limit the generalizability of our findings to younger age groups, structural epilepsies, and populations evaluated at other centers or using different imaging protocols. Future prospective, multicenter, and longitudinal studies should validate the EPVS phenotype across different pediatric epilepsy populations and characterize within-individual changes using serial MRI examinations. Such investigations would benefit from pediatric-validated segmentation methods, more detailed seizure and treatment data, quantitative electrophysiological measures, and imaging approaches that also encompass CSF compartments outside the ventricular system. Joint evaluation of EPVS with DTI-ALPS and complementary MRI markers sensitive to different aspects of perivascular fluid physiology within the same cohorts may further clarify the relationships among these imaging features and improve our understanding of the reproducibility, temporal evolution, and clinical relevance of the BG-predominant regional perivascular phenotype.

## 5. Conclusions

In this retrospective cross-sectional quantitative imaging study, the primary between-group analyses identified greater EPVS burden in pediatric epilepsy, with the most pronounced regional differences involving the BG and with additional differences in regional EPVS distribution. Exploratory within-epilepsy analyses identified associations between selected EPVS measures and several clinical characteristics; these associations should be interpreted as exploratory and do not establish prognostic or treatment-related significance. The principal contribution of these findings is the quantitative characterization of a BG-predominant regional EPVS phenotype in pediatric epilepsy rather than evidence for a specific glymphatic mechanism. EPVS, CP, and ventricular CSF measures are structural imaging phenotypes and do not directly measure glymphatic function. Longitudinal and multimodal studies, together with independent pediatric validation of EPVS segmentation, are needed to determine the biological and clinical relevance of these findings.

## Figures and Tables

**Table 1:** Quantitative MRI markers defining the enlarged perivascular space phenotype.

Phenotypic Domain	Quantitative MRI Marker	Definition/Calculation
**Global and hemispheric EPVS burden**	Total EPVS volume	Whole-brain EPVS volume, mm^3^
	Right hemispheric EPVS volume	EPVS volume within the right cerebral hemisphere, mm^3^
	Left hemispheric EPVS volume	EPVS volume within the left cerebral hemisphere, mm^3^
**Regional EPVS burden**	Right CSO-EPVS volume	EPVS volume within the right CSO-EPVS, mm^3^
	Left CSO-EPVS volume	EPVS volume within the left CSO-EPVS, mm^3^
	Total CSO-EPVS volume	Right CSO-EPVS volume + left CSO-EPVS volume, mm^3^
	Right BG-EPVS volume	EPVS volume within the right BG-EPVS, mm^3^
	Left BG-EPVS volume	EPVS volume within the left BG-EPVS, mm^3^
	Total BG-EPVS volume	Right BG-EPVS volume + left BG-EPVS volume, mm^3^
**EPVS asymmetry**	Hemispheric EPVS asymmetry index	(Right hemispheric EPVS volume − left hemispheric EPVS volume)/(right hemispheric EPVS volume + left hemispheric EPVS volume)
	CSO-EPVS asymmetry index	(Right CSO-EPVS volume − left CSO-EPVS volume)/(right CSO-EPVS volume + left CSO-EPVS volume)
	BG-EPVS asymmetry index	(Right BG-EPVS volume − left BG-EPVS volume)/(right BG-EPVS volume + left BG-EPVS volume)
**Relative regional EPVS burden**	Right CSO relative EPVS burden	Right CSO-EPVS volume/right hemispheric EPVS volume
	Left CSO relative EPVS burden	Left CSO-EPVS volume/left hemispheric EPVS volume
	Right BG relative EPVS burden	Right BG-EPVS volume/right hemispheric EPVS volume
	Left BG relative EPVS burden	Left BG-EPVS volume/left hemispheric EPVS volume
	Total CSO relative EPVS burden	Total CSO-EPVS volume/total EPVS volume
	Total BG relative EPVS burden	Total BG-EPVS volume/total EPVS volume
	Right hemispheric relative EPVS burden	Right hemispheric EPVS volume/total EPVS volume
	Left hemispheric relative EPVS burden	Left hemispheric EPVS volume/total EPVS volume
**Relative CSO-to-BG distribution**	Right CSO-to-BG EPVS volume ratio	Right CSO-EPVS volume/right BG-EPVS volume
	Left CSO-to-BG EPVS volume ratio	Left CSO-EPVS volume/left BG-EPVS volume

Five complementary EPVS phenotypic domains were defined: global and hemispheric EPVS burden, regional EPVS burden, EPVS asymmetry, relative regional EPVS burden, and within-hemisphere CSO-to-BG distribution. Volumetric measures are expressed in cubic millimeters. Total CSO-EPVS and total BG-EPVS volumes were calculated as the sums of the corresponding right- and left-sided regional volumes. Asymmetry indices were calculated as (R − L)/(R + L), with 0 indicating symmetry, positive values indicating right-sided predominance, and negative values indicating left-sided predominance; greater absolute values indicate a greater degree of asymmetry. Relative regional EPVS burden was calculated by normalizing regional EPVS volume to the corresponding hemispheric or whole-brain EPVS volume, as specified in the table. CSO-to-BG EPVS volume ratios represent the relative distribution of EPVS burden between the CSO and BG within each hemisphere, with values >1 indicating relative CSO predominance and values <1 indicating relative BG predominance. Abbreviations: BG, basal ganglia; CSO, centrum semiovale; EPVS, enlarged perivascular spaces; MRI, magnetic resonance imaging; R, right; L, left.

**Table 2:** Quantitative MRI Markers Defining the Choroid Plexus (CP) Phenotype.

Phenotypic Domain	Quantitative MRI Marker	Definition/Calculation
**Absolute CP volume**	Total CP volume	Right CP volume + left CP volume, mm^3^
	Right CP volume	Volume of the right CP, mm^3^
	Left CP volume	Volume of the left CP, mm^3^
**eTIV-normalized CP volume**	eTIV-normalized total CP volume	Total CP volume/eTIV
	eTIV-normalized right CP volume	Right CP volume/eTIV
	eTIV-normalized left CP volume	Left CP volume/eTIV
**CP asymmetry**	CP asymmetry index	(Right CP volume − left CP volume)/(right CP volume + left CP volume)
	Absolute CP asymmetry index	|CP asymmetry index|
**CP-to-lateral ventricle proportional measures**	Right CP-to-lateral ventricle volume ratio	Right CP volume/right lateral ventricle volume
	Left CP-to-lateral ventricle volume ratio	Left CP volume/left lateral ventricle volume
	Total CP-to-total lateral ventricle volume ratio	Total CP volume/(right lateral ventricle volume + left lateral ventricle volume)
**CP-to-ventricular CSF proportional measure**	Total CP-to-total ventricular CSF volume ratio	Total CP volume/total ventricular CSF volume

Five complementary CP phenotypic domains were defined: absolute CP volume, eTIV-normalized CP volume, CP asymmetry, CP volume relative to the lateral ventricular compartment, and CP volume relative to the overall ventricular CSF compartment. Absolute CP volumes are expressed in cubic millimeters (mm^3^), with total CP volume calculated as the sum of the right and left CP volumes. eTIV-normalized CP measures were calculated by dividing the corresponding CP volume by eTIV and are expressed as dimensionless ratios. The directional CP asymmetry index was calculated as (R − L)/(R + L), where 0 indicates bilateral symmetry, positive values indicate right-sided predominance, and negative values indicate left-sided predominance. The absolute asymmetry index, |(R − L)/(R + L)|, represents the magnitude of asymmetry irrespective of direction. Hemispheric CP-to-lateral ventricle volume ratios were calculated using the corresponding ipsilateral lateral ventricular volume, whereas the total CP-to-total lateral ventricle volume ratio was calculated using the summed right and left lateral ventricular volumes. Total ventricular CSF volume was defined as the sum of the right and left lateral ventricular, right and left inferior lateral ventricular, third ventricular, and fourth ventricular volumes. CP-to-ventricle ratios were treated as complementary proportional measures characterizing CP volume relative to the corresponding ventricular CSF compartment and were not interpreted as measures independent of ventricular size. Abbreviations: CP, choroid plexus; CSF, cerebrospinal fluid; MRI, magnetic resonance imaging; R, right; L, left; eTIV, estimated total intracranial volume.

**Table 3:** Quantitative MRI Measures of the Ventricular CSF Phenotype.

Domain	Quantitative MRI Measure	Definition/Calculation
**Absolute ventricular volume**	Right lateral ventricular volume	Right lateral ventricular volume, mm^3^
	Left lateral ventricular volume	Left lateral ventricular volume, mm^3^
	Right inferior lateral ventricular volume	Right inferior lateral ventricular volume, mm^3^
	Left inferior lateral ventricular volume	Left inferior lateral ventricular volume, mm^3^
	Third ventricular volume	Third ventricular volume, mm^3^
	Fourth ventricular volume	Fourth ventricular volume, mm^3^
**eTIV-normalized ventricular volume**	eTIV-normalized right lateral ventricular volume	Right lateral ventricular volume/eTIV
	eTIV-normalized left lateral ventricular volume	Left lateral ventricular volume/eTIV
	eTIV-normalized right inferior lateral ventricular volume	Right inferior lateral ventricular volume/eTIV
	eTIV-normalized left inferior lateral ventricular volume	Left inferior lateral ventricular volume/eTIV
	eTIV-normalized third ventricular volume	Third ventricular volume/eTIV
	eTIV-normalized fourth ventricular volume	Fourth ventricular volume/eTIV
**Ventricular asymmetry**	Lateral ventricular asymmetry index	(Right lateral ventricular volume − left lateral ventricular volume)/(right lateral ventricular volume + left lateral ventricular volume)
	Inferior lateral ventricular asymmetry index	(Right inferior lateral ventricular volume − left inferior lateral ventricular volume)/(right inferior lateral ventricular volume + left inferior lateral ventricular volume)

Quantitative ventricular CSF markers were organized into three complementary phenotypic domains: absolute ventricular volumes, eTIV-normalized ventricular volumes, and ventricular asymmetry. Absolute volumes represent compartment-specific volumes of the lateral, inferior lateral, third, and fourth ventricles and are expressed in cubic millimeters (mm^3^). Total lateral ventricular and total inferior lateral ventricular volumes were calculated as the sums of the corresponding right- and left-sided volumes. Total ventricular CSF volume was defined as the sum of the right and left lateral ventricular, right and left inferior lateral ventricular, third ventricular, and fourth ventricular volumes. eTIV-normalized measures were calculated by dividing the corresponding ventricular volume by eTIV to account for interindividual differences in intracranial size and are expressed as dimensionless ratios. Directional asymmetry indices for the lateral and inferior lateral ventricles were calculated as (R − L)/(R + L), where 0 indicates bilateral symmetry, positive values indicate right-sided predominance, and negative values indicate left-sided predominance; greater absolute values indicate a greater degree of asymmetry. Abbreviations: CSF, cerebrospinal fluid; MRI, magnetic resonance imaging; R, right; L, left; eTIV, estimated total intracranial volume.

**Table 4:** Demographic and clinical characteristics of the study groups.

Characteristic	Epilepsy (*n* = 123)	Control (*n* = 108)	*p* Value
**Demographic characteristics**			
**Age at MRI, years, mean ± SD**	14.4 ± 2.2	13.9 ± 2.3	0.103
**Sex, *n* (%)**			0.691
Female	61 (49.6)	51 (47.2)	
Male	62 (50.4)	57 (52.8)	
**eTIV, cm^3^** **, mean ± SD**	1512 ± 185	1475 ± 184	0.134
**Epilepsy-related clinical characteristics**			
**Age at epilepsy onset, years, mean ± SD**	8.4 ± 3.6	—	—
**Disease duration, years, mean ± SD**	5.9 ± 3.5	—	—
**Number of ASMs at MRI, median (IQR)**	1 (1–2)	—	—
**Treatment regimen, *n* (%)**		—	—
Monotherapy	70 (56.9)	—	—
Polytherapy	53 (43.1)	—	—
Drug-resistant epilepsy, *n* (%)	40 (32.5)	—	—
**Epilepsy type, *n* (%)**		—	—
Focal	73 (59.3)	—	—
Generalized	36 (29.3)	—	—
Combined generalized and focal	9 (7.3)	—	—
Unknown	5 (4.1)	—	—
**EEG characteristics**			
**EEG classification, *n* (%)**		—	—
Focal	62 (50.4)	—	—
Generalized	29 (23.6)	—	—
Multifocal	18 (14.6)	—	—
Normal	14 (11.4)	—	—
**EEG lateralization, *n* (%)**		—	—
Right	25 (31.3)	—	—
Left	27 (33.8)	—	—
Bilateral	28 (35.0)	—	—
**EEG anatomical localization, *n* (%)**			
**Temporal**	23 (28.8)	—	—
Right	8 (10.0)	—	—
Left	9 (11.3)	—	—
Bilateral	6 (7.5)	—	—
**Frontal**	17 (21.3)	—	—
Right	6 (7.5)	—	—
Left	5 (6.3)	—	—
Bilateral	6 (7.5)	—	—
**Centroparietal/posterior**	16 (20.0)	—	—
Right	5 (6.3)	—	—
Left	6 (7.5)	—	—
Bilateral	5 (6.3)	—	—
**Multifocal**	18 (22.5)	—	—
Right-predominant	4 (5.0)	—	—
Left-predominant	5 (6.3)	—	—
Bilateral	9 (11.3)	—	—
**Indeterminate**	6 (7.5)	—	—
Right-lateralized	2 (2.5)	—	—
Left-lateralized	2 (2.5)	—	—
Bilateral	2 (2.5)	—	—

Data are presented as mean ± SD, median (IQR), or *n* (%), as indicated. Percentages may not total 100% because of rounding. Between-group comparisons were performed only for variables applicable to both study groups. Continuous variables were compared using the independent-samples *t* test or Welch *t* test, as appropriate, and categorical variables using the Pearson χ^2^ test or Fisher exact test, as appropriate. No between-group statistical comparisons were applicable to epilepsy-specific clinical or EEG characteristics. An em dash indicates not applicable. EEG lateralization and anatomical localization were evaluated only among patients with focal or multifocal epileptiform activity (*n* = 80). Percentages for these variables, including the directional subcategories of anatomical localization, were calculated using this subgroup as the denominator. Directional subcategories indicate the hemispheric distribution of the corresponding epileptiform abnormalities. Abbreviations: ASM, antiseizure medication; EEG, electroencephalography; IQR, interquartile range; MRI, magnetic resonance imaging; SD, standard deviation; eTIV, estimated total intracranial volume.

**Table 5:** Comparison of EPVS Phenotypes Between Epilepsy and Control Groups.

Variable	Epilepsy, Adjusted EMM (95% CI)	Control, Adjusted EMM (95% CI)	Adjusted Difference (95% CI)	*p* Value	FDR-Adjusted *q* Value
**Global and hemispheric EPVS burden**					
Total EPVS volume, mm^3^	2134.9 (2028.7–2241.1)	1850.2 (1736.8–1963.6)	284.7 (105.9–463.5)	0.001	0.003
Right hemispheric EPVS volume, mm^3^	1086.7 (1030.2–1143.2)	942.1 (881.8–1002.4)	144.6 (47.8–241.4)	0.003	0.005
Left hemispheric EPVS volume, mm^3^	1048.2 (991.0–1105.4)	908.1 (847.0–969.2)	140.1 (43.2–237.0)	0.004	0.007
**Regional EPVS burden**					
Right CSO-EPVS volume, mm^3^	671.4 (634.1–708.7)	651.8 (612.0–691.6)	19.6 (−27.2–66.4)	0.412	0.432
Left CSO-EPVS volume, mm^3^	659.7 (622.8–696.6)	638.9 (599.5–678.3)	20.8 (−24.2–65.8)	0.365	0.402
Total CSO-EPVS volume, mm^3^	1331.1 (1266.7–1395.5)	1290.7 (1221.9–1359.5)	40.4 (−42.2–123.0)	0.338	0.391
Right BG-EPVS volume, mm^3^	211.9 (193.5–230.3)	127.4 (107.8–147.0)	84.5 (43.2–125.8)	<0.001	<0.001
Left BG-EPVS volume, mm^3^	196.7 (179.1–214.3)	120.3 (101.5–139.1)	76.4 (38.2–114.6)	<0.001	<0.001
Total BG-EPVS volume, mm^3^	408.6 (377.3–439.9)	247.7 (214.3–281.1)	160.9 (89.5–232.3)	<0.001	<0.001
**EPVS asymmetry**					
Hemispheric EPVS asymmetry index	0.019 (0.008–0.030)	0.015 (0.003–0.027)	0.004 (−0.003–0.011)	0.284	0.347
CSO-EPVS asymmetry index	0.012 (0.001–0.023)	0.010 (−0.002–0.022)	0.002 (−0.006–0.010)	0.611	0.611
BG-EPVS asymmetry index	0.046 (0.032–0.060)	0.033 (0.018–0.048)	0.013 (−0.001–0.027)	0.073	0.107
**Relative regional EPVS burden**					
Right CSO relative EPVS burden	0.618 (0.598–0.638)	0.692 (0.671–0.713)	−0.074 (−0.117 to −0.031)	<0.001	0.001
Left CSO relative EPVS burden	0.629 (0.608–0.650)	0.704 (0.682–0.726)	−0.074 (−0.118 to −0.031)	<0.001	0.001
Right BG relative EPVS burden	0.195 (0.182–0.208)	0.135 (0.121–0.149)	0.060 (0.032–0.088)	<0.001	<0.001
Left BG relative EPVS burden	0.188 (0.175–0.201)	0.132 (0.118–0.146)	0.055 (0.029–0.082)	<0.001	<0.001
Total CSO relative EPVS burden	0.623 (0.604–0.642)	0.698 (0.678–0.718)	−0.074 (−0.115 to −0.033)	<0.001	<0.001
Total BG relative EPVS burden	0.191 (0.179–0.203)	0.134 (0.121–0.147)	0.058 (0.032–0.083)	<0.001	<0.001
Right hemispheric relative EPVS burden	0.509 (0.499–0.519)	0.509 (0.498–0.520)	−0.0002 (−0.0006–0.0002)	0.284	0.347
Left hemispheric relative EPVS burden	0.491 (0.481–0.501)	0.491 (0.480–0.502)	0.0002 (−0.0002–0.0006)	0.284	0.347
**Relative CSO-to-BG distribution**					
Right CSO-to-BG EPVS volume ratio	3.168 (2.748–3.588)	5.116 (4.668–5.564)	−1.948 (−2.843 to −1.053)	<0.001	<0.001
Left CSO-to-BG EPVS volume ratio	3.354 (2.909–3.799)	5.311 (4.836–5.786)	−1.957 (−2.857 to −1.058)	<0.001	<0.001

Data are presented as adjusted estimated marginal means (EMMs) with 95% confidence intervals (CIs). Adjusted differences were calculated as epilepsy minus control and are reported with corresponding 95% CIs. All models were adjusted for age and sex; models of absolute EPVS volumes were additionally adjusted for estimated total intracranial volume (eTIV). Absolute EPVS volumes are expressed in mm^3^ and were adjusted for eTIV as a covariate rather than normalized to eTIV. eTIV was not included in models of asymmetry indices or dimensionless relative-distribution measures. Relative regional burden measures represent the proportional distribution of EPVS burden across the predefined anatomical compartments. Asymmetry indices, relative regional burden measures, and CSO-to-BG ratios are dimensionless. Reported *p* values are two-sided, unadjusted values for the group effect. All quantitative EPVS phenotype comparisons were treated as a single hypothesis family and adjusted using the Benjamini–Hochberg false discovery rate (FDR) procedure. FDR-adjusted *q* values are reported alongside the corresponding unadjusted *p* values, with *q* < 0.05 considered statistically significant. Abbreviations: BG, basal ganglia; CI, confidence interval; CSO, centrum semiovale; EMM, estimated marginal mean; EPVS, enlarged perivascular spaces; FDR, false discovery rate; eTIV, estimated total intracranial volume.

**Table 6:** Comparison of choroid plexus phenotypes between the epilepsy and control groups.

Variable	Epilepsy, Adjusted EMM (95% CI)	Control, Adjusted EMM (95% CI)	Adjusted Difference (95% CI)	*p* Value	FDR-Adjusted *q* Value
**Absolute CP volumes**					
Right CP volume, mm^3^	842.7 (801.4–884.0)	814.6 (770.5–858.7)	28.1 (−32.3–88.5)	0.361	0.619
Left CP volume, mm^3^	825.3 (785.0–865.6)	798.9 (755.9–841.9)	26.4 (−32.5–85.3)	0.378	0.619
Total CP volume, mm^3^	1668.0 (1592.4–1743.6)	1613.5 (1532.8–1694.2)	54.5 (−56.1–165.1)	0.333	0.619
**eTIV-normalized CP volumes**					
eTIV-normalized right CP	5.58 × 10^−4^ (5.31–5.85 × 10^−4^)	5.52 × 10^−4^ (5.23–5.81 × 10^−4^)	0.06 × 10^−4^ (−0.34–0.46 × 10^−4^)	0.768	0.922
eTIV-normalized left CP	5.46 × 10^−4^ (5.20–5.72 × 10^−4^)	5.41 × 10^−4^ (5.13–5.69 × 10^−4^)	0.05 × 10^−4^ (−0.34–0.44 × 10^−4^)	0.801	0.922
eTIV-normalized total CP	1.104 × 10^−3^ (1.055–1.153 × 10^−3^)	1.093 × 10^−3^ (1.041–1.145 × 10^−3^)	0.011 × 10^−3^ (−0.060–0.082 × 10^−3^)	0.760	0.922
**CP asymmetry**					
CP asymmetry index	0.010 (−0.006–0.026)	0.009 (−0.008–0.026)	0.001 (−0.022–0.024)	0.932	0.932
Absolute CP asymmetry index	0.074 (0.064–0.084)	0.071 (0.060–0.082)	0.003 (−0.012–0.018)	0.694	0.926
**CP-to-lateral ventricle proportional measures**					
Right CP-to-right lateral ventricle ratio	0.148 (0.135–0.161)	0.141 (0.127–0.155)	0.007 (−0.012–0.026)	0.469	0.704
Left CP-to-left lateral ventricle ratio	0.144 (0.131–0.157)	0.138 (0.124–0.152)	0.006 (−0.013–0.025)	0.535	0.713
Total CP-to-total lateral ventricle ratio	0.146 (0.134–0.158)	0.140 (0.127–0.153)	0.006 (−0.012–0.024)	0.511	0.713
**CP-to-total ventricular CSF proportional measure**					
Total CP-to-total ventricular CSF ratio	0.119 (0.109–0.129)	0.115 (0.104–0.126)	0.004 (−0.011–0.019)	0.599	0.799

Data are presented as adjusted estimated marginal means (EMMs) with 95% confidence intervals (CIs). Adjusted differences were calculated as epilepsy minus control and are reported with corresponding 95% CIs. All models were adjusted for age and sex; models of absolute CP volumes were additionally adjusted for estimated total intracranial volume (eTIV). eTIV-normalized CP measures, CP asymmetry indices, and proportional CP measures were analyzed without additional adjustment for eTIV. Absolute CP volumes are expressed in mm^3^, whereas eTIV-normalized volumes, asymmetry indices, and proportional measures are dimensionless. Reported *p* values are two-sided, unadjusted values for the group effect. All quantitative CP phenotype comparisons were treated as a single hypothesis family and adjusted using the Benjamini–Hochberg false discovery rate (FDR) procedure. FDR-adjusted *q* values are reported alongside the corresponding unadjusted *p* values, with *q* < 0.05 considered statistically significant. Abbreviations: CI, confidence interval; CP, choroid plexus; CSF, cerebrospinal fluid; EMM, estimated marginal mean; FDR, false discovery rate; eTIV, estimated total intracranial volume.

**Table 7:** Comparison of ventricular CSF phenotypes between the epilepsy and control groups.

Variable	Epilepsy, Adjusted EMM (95% CI)	Control, Adjusted EMM (95% CI)	Adjusted Difference (95% CI)	*p* Value	FDR-Adjusted *q* Value
Absolute ventricular volumes					
Right lateral ventricular volume, mm^3^	6038.4 (5787.2–6289.6)	5876.9 (5608.8–6145.0)	161.5 (−95.7–418.7)	0.218	0.623
Left lateral ventricular volume, mm^3^	6204.7 (5943.1–6466.3)	6033.8 (5754.6–6313.0)	170.9 (−120.4–462.2)	0.247	0.623
Total lateral ventricular volume, mm^3^	12,243.1 (11,755.8–12,730.4)	11,910.7 (11,390.8–12,430.6)	332.4 (−195.1–859.9)	0.224	0.623
Right inferior lateral ventricular volume, mm^3^	397.8 (366.2–429.4)	385.1 (351.4–418.8)	12.7 (−34.0–59.4)	0.412	0.623
Left inferior lateral ventricular volume, mm^3^	421.6 (388.0–455.2)	410.2 (374.4–446.0)	11.4 (−38.2–61.0)	0.465	0.623
Total inferior lateral ventricular volume, mm^3^	819.4 (758.3–880.5)	795.3 (730.1–860.5)	24.1 (−66.3–114.5)	0.438	0.623
Third ventricular volume, mm^3^	742.5 (706.8–778.2)	721.4 (683.3–759.5)	21.1 (−28.3–70.5)	0.331	0.623
Fourth ventricular volume, mm^3^	1517.9 (1458.2–1577.6)	1496.8 (1433.1–1560.5)	21.1 (−62.4–104.6)	0.286	0.623
Total ventricular CSF volume, mm^3^	15,322.9 (14,756.5–15,889.3)	14,924.2 (14,319.7–15,528.7)	398.7 (−238.5–1035.9)	0.273	0.623
eTIV-normalized ventricular volumes					
eTIV-normalized right lateral ventricular volume	0.00403 (0.00386–0.00420)	0.00398 (0.00380–0.00416)	0.00005 (−0.00006–0.00016)	0.356	0.623
eTIV-normalized left lateral ventricular volume	0.00414 (0.00396–0.00432)	0.00409 (0.00390–0.00428)	0.00005 (−0.00007–0.00017)	0.392	0.623
eTIV-normalized total lateral ventricular volume	0.00817 (0.00785–0.00849)	0.00807 (0.00773–0.00841)	0.00010 (−0.00011–0.00031)	0.371	0.623
eTIV-normalized right inferior lateral ventricular volume	0.000266 (0.000245–0.000287)	0.000261 (0.000239–0.000283)	0.000005 (−0.000024–0.000034)	0.524	0.634
eTIV-normalized left inferior lateral ventricular volume	0.000281 (0.000259–0.000303)	0.000278 (0.000255–0.000301)	0.000003 (−0.000027–0.000033)	0.571	0.634
eTIV-normalized total inferior lateral ventricular volume	0.000547 (0.000507–0.000587)	0.000539 (0.000497–0.000581)	0.000008 (−0.000047–0.000063)	0.548	0.634
eTIV-normalized third ventricular volume	0.000495 (0.000472–0.000518)	0.000489 (0.000464–0.000514)	0.000006 (−0.000026–0.000038)	0.467	0.623
eTIV-normalized fourth ventricular volume	0.001014 (0.000974–0.001054)	0.001008 (0.000965–0.001051)	0.000006 (−0.000049–0.000061)	0.419	0.623
eTIV-normalized total ventricular CSF volume	0.01023 (0.00985–0.01061)	0.01011 (0.00970–0.01052)	0.00012 (−0.00014–0.00038)	0.404	0.623
Ventricular asymmetry					
Lateral ventricular asymmetry index	−0.014 (−0.028–0.000)	−0.013 (−0.028–0.002)	−0.001 (−0.021–0.019)	0.683	0.719
Inferior lateral ventricular asymmetry index	−0.028 (−0.046 to −0.010)	−0.031 (−0.050 to −0.012)	0.003 (−0.022–0.028)	0.741	0.741

Data are presented as adjusted estimated marginal means (EMMs) with 95% confidence intervals (CIs). Adjusted differences were calculated as epilepsy minus control and are reported with corresponding 95% CIs. All models were adjusted for age and sex; models of absolute ventricular volumes were additionally adjusted for estimated total intracranial volume (eTIV). eTIV-normalized ventricular measures and ventricular asymmetry indices were analyzed without additional adjustment for eTIV. Absolute ventricular volumes are expressed in mm^3^, whereas eTIV-normalized volumes and asymmetry indices are dimensionless. Reported *p* values are two-sided, unadjusted values for the group effect. All quantitative ventricular CSF phenotype comparisons were treated as a single hypothesis family and adjusted using the Benjamini–Hochberg false discovery rate (FDR) procedure. FDR-adjusted *q* values are reported alongside the corresponding unadjusted *p* values, with *q* < 0.05 considered statistically significant. Abbreviations: CI, confidence interval; CSF, cerebrospinal fluid; EMM, estimated marginal mean; FDR, false discovery rate; eTIV, estimated total intracranial volume.

**Table 8:** Interobserver reproducibility of quantitative imaging measurements.

Quantitative Imaging Measure	ICC	95% CI
**EPVS phenotype**		
Total EPVS volume	0.87	0.77–0.93
Right hemispheric EPVS volume	0.83	0.70–0.90
Left hemispheric EPVS volume	0.85	0.74–0.92
Right CSO-EPVS volume	0.79	0.64–0.88
Left CSO-EPVS volume	0.81	0.68–0.89
Total CSO-EPVS volume	0.84	0.72–0.91
Right BG-EPVS volume	0.71	0.52–0.83
Left BG-EPVS volume	0.74	0.57–0.85
Total BG-EPVS volume	0.77	0.61–0.86
**Choroid plexus phenotype**		
Right CP volume	0.87	0.77–0.93
Left CP volume	0.89	0.80–0.94
Total CP volume	0.92	0.85–0.96
**Ventricular CSF phenotype**		
Right lateral ventricular volume	0.88	0.79–0.94
Left lateral ventricular volume	0.89	0.80–0.94
Total lateral ventricular volume	0.91	0.84–0.95
Right inferior lateral ventricular volume	0.76	0.59–0.86
Left inferior lateral ventricular volume	0.79	0.64–0.88
Total inferior lateral ventricular volume	0.82	0.69–0.90
Third ventricular volume	0.85	0.74–0.92
Fourth ventricular volume	0.84	0.72–0.91
Total ventricular CSF volume	0.93	0.87–0.96

Interobserver reproducibility was evaluated in a randomly selected subset of 40 participants (20 with epilepsy and 20 controls). Volumetric agreement between two independent observers was assessed using a two-way mixed-effects, absolute-agreement, single-measurement intraclass correlation coefficient (ICC), with 95% confidence intervals (CIs). ICC values were interpreted as <0.50, poor; 0.50–<0.75, moderate; 0.75–0.90, good; and >0.90, excellent reliability. Abbreviations: BG, basal ganglia; CI, confidence interval; CP, choroid plexus; CSF, cerebrospinal fluid; CSO, centrum semiovale; EPVS, enlarged perivascular spaces; ICC, intraclass correlation coefficient.

**Table 9:** Adjusted associations of continuous and binary clinical characteristics with FDR-significant EPVS measures in the epilepsy group.

Continuous Clinical Characteristics				
Clinical Variable	EPVS Measure	Adjusted B (95% CI)	*p* Value	FDR-Adjusted *q* Value
Age at epilepsy onset	Total EPVS volume	−29.6 (−63.8 to 4.6)	0.089	0.146
	Right BG-EPVS volume	−5.1 (−8.9 to −1.3)	0.010	0.034
	Left BG-EPVS volume	−4.7 (−8.3 to −1.1)	0.012	0.038
	Total BG-EPVS volume	−9.8 (−17.1 to −2.5)	0.009	0.032
	Total BG relative EPVS burden	−0.0028 (−0.0051 to −0.0005)	0.018	0.046
Disease duration	Total EPVS volume	48.7 (25.1 to 72.3)	<0.001	0.002
	Right hemispheric EPVS volume	25.9 (12.7 to 39.1)	<0.001	0.002
	Left hemispheric EPVS volume	23.8 (10.9 to 36.7)	<0.001	0.003
	Right BG-EPVS volume	8.1 (4.8 to 11.4)	<0.001	<0.001
	Left BG-EPVS volume	7.4 (4.2 to 10.6)	<0.001	<0.001
	Total BG-EPVS volume	15.5 (10.2 to 20.8)	<0.001	<0.001
	Total BG relative EPVS burden	0.0040 (0.0023 to 0.0057)	<0.001	<0.001
	Total CSO relative EPVS burden	−0.0045 (−0.0068 to −0.0022)	<0.001	0.001
	Right CSO-to-BG EPVS volume ratio	−0.101 (−0.151 to −0.051)	<0.001	0.002
	Left CSO-to-BG EPVS volume ratio	−0.094 (−0.147 to −0.041)	0.001	0.004
Number of ASMs at MRI	Total EPVS volume	81.7 (18.2 to 145.2)	0.012	0.051
	Right BG-EPVS volume	13.6 (5.6 to 21.6)	0.001	0.009
	Left BG-EPVS volume	12.2 (4.5 to 19.9)	0.002	0.013
	Total BG-EPVS volume	25.8 (11.0 to 40.6)	0.001	0.008
	Total BG relative EPVS burden	0.0061 (0.0019 to 0.0103)	0.005	0.024
	Right CSO-to-BG EPVS volume ratio	−0.134 (−0.241 to −0.027)	0.015	0.049
**Binary clinical characteristics**				
**Clinical variable**	**EPVS measure**	**Adjusted difference (95% CI)**	***p* value**	**FDR *q* value**
Drug-resistant epilepsy	Total EPVS volume	237.4 (96.6 to 378.2)	0.001	0.006
	Right hemispheric EPVS volume	125.8 (48.9 to 202.7)	0.002	0.010
	Left hemispheric EPVS volume	116.3 (40.8 to 191.8)	0.003	0.013
	Right BG-EPVS volume	49.9 (28.7 to 71.1)	<0.001	<0.001
	Left BG-EPVS volume	45.2 (24.9 to 65.5)	<0.001	<0.001
	Total BG-EPVS volume	95.1 (61.5 to 128.7)	<0.001	<0.001
	Total BG relative EPVS burden	0.020 (0.010 to 0.030)	<0.001	0.002
	Total CSO relative EPVS burden	−0.024 (−0.039 to −0.009)	0.002	0.010
	Right CSO-to-BG EPVS volume ratio	−0.61 (−0.99 to −0.23)	0.002	0.010
	Left CSO-to-BG EPVS volume ratio	−0.57 (−0.97 to −0.17)	0.006	0.027
Monotherapy/Polytherapy	Total EPVS volume	151.8 (20.4 to 283.2)	0.024	0.063
	Right BG-EPVS volume	33.8 (16.1 to 51.5)	<0.001	0.005
	Left BG-EPVS volume	31.1 (13.9 to 48.3)	0.001	0.007
	Total BG-EPVS volume	64.9 (31.1 to 98.7)	<0.001	0.004
	Total BG relative EPVS burden	0.014 (0.004 to 0.024)	0.006	0.027
	Right CSO-to-BG EPVS volume ratio	−0.39 (−0.73 to −0.05)	0.025	0.048
	Left CSO-to-BG EPVS volume ratio	−0.35 (−0.70 to 0.00)	0.051	0.096

Clinical association analyses were restricted to EPVS measures showing significant epilepsy–control differences after FDR correction and were performed within the epilepsy group. For continuous clinical variables, adjusted effects are presented as unstandardized regression coefficients (*B*) with 95% confidence intervals (CIs), representing the expected change in the corresponding EPVS measure per one-unit increase in the clinical variable. For binary clinical characteristics, adjusted effects are presented as model-derived between-group differences; for drug resistance, positive values indicate higher EPVS values in patients with drug-resistant epilepsy, whereas for treatment regimen, positive values indicate higher EPVS values in patients receiving polytherapy than in those receiving monotherapy. All models were adjusted for age and sex; models of absolute EPVS volumes were additionally adjusted for estimated total intracranial volume (eTIV). eTIV was not additionally included in models of relative EPVS burden measures or CSO-to-BG ratios. Reported *p* values are two-sided and unadjusted for multiple comparisons. Clinical-association *p* values were adjusted within the 64-test exploratory clinical-association FDR family using the Benjamini–Hochberg procedure; the complete predictor–outcome matrix and multiplicity structure are reported in Supplementary Table S6. FDR-adjusted *q* values are reported alongside the corresponding unadjusted *p* values, with *q* < 0.05 considered statistically significant. Abbreviations: ASM, antiseizure medication; BG, basal ganglia; CI, confidence interval; CSO, centrum semiovale; EPVS, enlarged perivascular spaces; FDR, false discovery rate; MRI, magnetic resonance imaging; eTIV, estimated total intracranial volume.

**Table 10:** Adjusted associations of epilepsy type and EEG characteristics with FDR-significant EPVS measures.

Clinical Variable/Category	EPVS Measure	Adjusted EMM (95% CI)	Omnibus *F*	*p* Value	FDR-Adjusted *q* Value
**Epilepsy type**	**Total EPVS volume**	—	1.32	0.271	0.374
Focal		2118 (2029–2207)	—	—	—
Generalized		2056 (1957–2155)	—	—	—
Combined generalized and focal		2167 (2021–2313)	—	—	—
Unknown		2094 (1961–2227)	—	—	—
**Epilepsy type**	**Total BG-EPVS volume**	—	1.81	0.149	0.252
Focal		452 (427–477)	—	—	—
Generalized		425 (397–453)	—	—	—
Combined generalized and focal		466 (425–507)	—	—	—
Unknown		438 (401–475)	—	—	—
**EEG classification**	**Total EPVS volume**	—	1.76	0.159	0.261
Normal		2054 (1948–2160)	—	—	—
Focal epileptiform		2146 (2053–2239)	—	—	—
Generalized epileptiform		2078 (1972–2184)	—	—	—
Multifocal epileptiform		2191 (2048–2334)	—	—	—
**EEG classification**	**Total BG-EPVS volume**	—	2.71	0.048	0.104
Normal		417 (386–448)	—	—	—
Focal epileptiform		451 (425–477)	—	—	—
Generalized epileptiform		431 (401–461)	—	—	—
Multifocal epileptiform		472 (432–512)	—	—	—
**EEG lateralization**	**Total EPVS volume**	—	3.24	0.045	0.094
Right		2102 (1995–2209)	—	—	—
Left		2118 (2014–2222)	—	—	—
Bilateral		2247 (2143–2351)	—	—	—
**EEG lateralization**	**Right BG-EPVS volume**	—	5.21	0.007	0.029
Right		216 (198–234)	—	—	—
Left		219 (201–237)	—	—	—
Bilateral		248 (229–267)	—	—	—
**EEG lateralization**	**Left BG-EPVS volume**	—	4.66	0.012	0.041
Right		213 (195–231)	—	—	—
Left		215 (198–232)	—	—	—
Bilateral		242 (224–260)	—	—	—
**EEG lateralization**	**Total BG-EPVS volume**	—	5.74	0.005	0.024
Right		429 (401–457)	—	—	—
Left		434 (406–462)	—	—	—
Bilateral		490 (462–518)	—	—	—
**EEG lateralization**	**Total BG relative EPVS burden**	—	4.91	0.010	0.037
Right		0.205 (0.193–0.217)	—	—	—
Left		0.207 (0.195–0.219)	—	—	—
Bilateral		0.229 (0.216–0.242)	—	—	—
**EEG anatomical localization**	**Total EPVS volume**	—	1.37	0.251	0.361
Temporal		2143 (2027–2259)	—	—	—
Frontal		2167 (2041–2293)	—	—	—
Centroparietal/posterior		2098 (1962–2234)	—	—	—
Multifocal		2214 (2072–2356)	—	—	—
Indeterminate		2112 (1973–2251)	—	—	—
**EEG anatomical localization**	**Total BG-EPVS volume**	—	2.02	0.099	0.187
Temporal		447 (414–480)	—	—	—
Frontal		455 (420–490)	—	—	—
Centroparietal/posterior		432 (394–470)	—	—	—
Multifocal		474 (434–514)	—	—	—
Indeterminate		441 (403–479)	—	—	—

Categorical clinical and EEG characteristics were evaluated using covariate-adjusted general linear models. Category-specific values are presented as adjusted estimated marginal means (EMMs) with 95% confidence intervals (CIs), whereas omnibus *F* statistics and corresponding two-sided *p* values represent the overall effect of each categorical variable on the respective EPVS measure. All models were adjusted for age and sex; models of absolute EPVS volumes were additionally adjusted for estimated total intracranial volume (eTIV). eTIV was not additionally included in models of relative EPVS burden measures. EEG lateralization and EEG anatomical localization analyses were restricted to participants for whom the corresponding characteristic could be defined and category sizes were sufficient for formal analysis. Category-specific EMMs were not interpreted as pairwise differences unless the corresponding omnibus effect remained significant after FDR correction. Pairwise EMM contrasts were performed only for omnibus effects that remained significant after FDR correction and were adjusted using the Tukey procedure. All omnibus clinical-association tests contributed to the same prespecified EPVS clinical-association hypothesis family as the analyses reported in Table 9 and were adjusted using the Benjamini–Hochberg false discovery rate (FDR) procedure. FDR-adjusted *q* values are reported alongside the corresponding unadjusted *p* values, with *q* < 0.05 considered statistically significant. Abbreviations: BG, basal ganglia; CI, confidence interval; EEG, electroencephalography; EMM, estimated marginal mean; EPVS, enlarged perivascular spaces; FDR, false discovery rate; eTIV, estimated total intracranial volume.

**Table 11:** Post-hoc comparisons for FDR-significant EEG lateralization effects on EPVS measures.

EPVS Measure	Pairwise Comparison	Adjusted Difference (95% CI)	Tukey-Adjusted *p* Value
**Right BG-EPVS volume**	Bilateral vs. Right	32 (8 to 56)	0.010
	Bilateral vs. Left	29 (5 to 53)	0.017
	Right vs. Left	−3 (−27 to 21)	0.949
**Left BG-EPVS volume**	Bilateral vs. Right	29 (5 to 53)	0.018
	Bilateral vs. Left	27 (4 to 50)	0.022
	Right vs. Left	−2 (−25 to 21)	0.978
**Total BG-EPVS volume**	Bilateral vs. Right	61 (23 to 99)	0.003
	Bilateral vs. Left	56 (18 to 94)	0.006
	Right vs. Left	−5 (−43 to 33)	0.944
**Total BG relative EPVS burden**	Bilateral vs. Right	0.024 (0.007 to 0.041)	0.006
	Bilateral vs. Left	0.022 (0.005 to 0.039)	0.011
	Right vs. Left	−0.002 (−0.019 to 0.015)	0.958

Post hoc pairwise comparisons were performed only for EPVS measures showing an FDR-significant omnibus effect of EEG lateralization. Adjusted differences were derived from estimated marginal mean contrasts and are expressed as the first category minus the second category. All models were adjusted for age and sex; models of absolute EPVS volumes were additionally adjusted for estimated total intracranial volume (eTIV). eTIV was not additionally included in the model of relative EPVS burden. *p* values for pairwise comparisons were adjusted for multiple comparisons using the Tukey procedure. Abbreviations: BG, basal ganglia; CI, confidence interval; EEG, electroencephalography; EPVS, enlarged perivascular spaces; FDR, false discovery rate; eTIV, estimated total intracranial volume.

## Data Availability

The data presented in this study are available on request from the corresponding author due to ethical and privacy restrictions.

## Supplementary Materials

The following supporting information can be downloaded at: https://www.mdpi.com/article/10.3390/diagnostics16203304/s1. Table S1: Manual Correction Burden and Automated-to-Corrected Mask Agreement of SHIVA-PVS Segmentations; Table S2: Sensitivity Analysis of EPVS Phenotypes Using Initial Unedited SHIVA-PVS Masks Compared With the Primary Expert-Corrected Masks; Table S3: Probability-Threshold Sensitivity Analysis of EPVS Phenotypes; Table S4: Model Diagnostics, Adjusted Standardized Effect Sizes, and Robustness Analyses for Between-Group EPVS Phenotype Comparisons; Table S5: Clinical Predictor Interrelationships, Collinearity Diagnostics, and Sensitivity Analyses of Clinical Associations With EPVS Measures; Table S6: Complete Clinical Predictor–Outcome Matrix and Reproducibility of False Discovery Rate Correction for the Exploratory Clinical-Association Analyses; Table S7: Selection-Independent Sensitivity Analysis of Clinical Associations Across an Expanded EPVS Outcome Set; Table S8: Full-Precision Raw *p*-Value Vectors for Reproduction of the Benjamini–Hochberg False Discovery Rate Adjustments. Files S1–S4: Statistical Code.

## Abbreviations

The following abbreviations are used in this manuscript:

3D	Three-dimensional
AI	Asymmetry index
ASM	Antiseizure medication
BG	Basal ganglia
CI	Confidence interval
CP	Choroid plexus
CSF	Cerebrospinal fluid
CSO	Centrum semiovale
DICOM	Digital Imaging and Communications in Medicine
DSC	Dice similarity coefficient
DTI	Diffusion tensor imaging
DTI-ALPS	Diffusion tensor imaging analysis along the perivascular space
DWI	Diffusion-weighted imaging
EEG	Electroencephalography
EMM	Estimated marginal mean
EPVS	Enlarged perivascular spaces
eTIV	Estimated total intracranial volume
FDR	False discovery rate
FLAIR	Fluid-attenuated inversion recovery
FOV	Field of view
ICC	Intraclass correlation coefficient
ILAE	International League Against Epilepsy
IQR	Interquartile range
MRI	Magnetic resonance imaging
NIfTI	Neuroimaging Informatics Technology Initiative
PACS	Picture Archiving and Communication System
R	Right
L	Left
SE	Spin echo
STROBE	Strengthening the Reporting of Observational Studies in Epidemiology
TE	Echo time
TI	Inversion time
TR	Repetition time
TSE	Turbo spin echo
