# Supplementary material for: Quantitative MRI Characterization of Fluid-Related Phenotypes in Pediatric Epilepsy: Deep Learning-Assisted Assessment of Enlarged Perivascular Space Burden, Regional Distribution, and Clinical Associations
Source: Diagnostics (Basel). 2026 Oct 11;16(20):3304. doi: 10.3390/diagnostics16203304 (PMC13703179; doi:10.3390/diagnostics16203304)
Supplement: Supplementary file 1 [file diagnostics-16-03304-s001.zip › Supplementary Material S1.pdf]

**Supplementary Material S1. Command-line implementation of the SHIVA-PVS-based PVS segmentation pipeline and preparation of outputs for expert quality control**

The following command-line workflow summarizes the computational pipeline used for multimodal T1-weighted/FLAIR PVS segmentation, including SHiVAi Docker deployment, SynthSeg-based brain segmentation within the SHiVAi workflow, v4/T1+FLAIR-PVS model configuration, automated PVS segmentation, and export of segmentation outputs for expert anatomical verification and manual correction in 3D Slicer.

**Step 1. Build SHiVAi Docker image**

```
cd /path/to/SHiVAi
docker build --rm \
-t shivai/shivai:0.5.9 \
-t shivai/shivai:latest \
```

**Step 2. Build SynthSeg Docker image**

```
cd /path/to/SHiVAi/apptainer
docker build --rm \
-t shivai/synthseg_shivai:latest \
-f synthseg.Dockerfile \
```

**Step 3. Verify Docker images**

```
docker images | grep shivai
```

**Step 4. Organize NIfTI input data in BIDS format**

```
mkdir -p /path/to/project/BIDS_dataset/sub-001/anat
cp /path/to/nifti/sub-001_T1w.nii.gz \
/path/to/project/BIDS_dataset/sub-001/anat/
cp /path/to/nifti/sub-001_FLAIR.nii.gz \
/path/to/project/BIDS_dataset/sub-001/anat/
```

**Step 5. Prepare the SHiVAi configuration file**

```
cat > /path/to/project/config.yml <<'EOF'
model_path: /path/to/project/shivai_models
docker_image: shivai/shivai:0.5.9
synthseg_docker_image: shivai/synthseg_shivai:latest
container_runtime: docker
parameters:
  brainmask_descriptor:
  PVS_descriptor:
  PVS2_descriptor: "T1-FLAIR.PVS/model_info_t1-flair-pvs-v4.json"
  WMH_descriptor:
  CMB_descriptor:
  LAC_descriptor:
  percentile: 99.0
  threshold: 0.5
  threshold_pvs: 0.5
  min_pvs_size: 5
  final_dimensions: [160, 214, 176]
  voxels_size: [1.0, 1.0, 1.0]
  voxels_tolerance: [0, 0, 0]
  interpolation: 'WelchWindowedSinc'
EOF
```

**Step 6. Install the SHiVAi command-line package**

```
cd /path/to/SHiVAi
python3 -m venv /path/to/shivai_env
source /path/to/shivai_env/bin/activate
pip install .
shiva --help
```

**Step 7. Run multimodal v4/T1+FLAIR-PVS segmentation using SynthSeg-based brain segmentation**

```
source /path/to/shivai_env/bin/activate
shiva \
--in /path/to/project/BIDS_dataset \
--out /path/to/project/shivai_results \
--input_type BIDS \
--prediction PVS2 \
--brain_seg synthseg \
--containerized_nodes \
--config /path/to/project/config.yml
```

**Step 8. Verify SHiVAi PVS outputs**

```
find /path/to/project/shivai_results -type f \
\( -iname "*pvs*.nii*" -o -iname "*pvs*.csv" -o -iname "*pvs*.json" \) \
-print
```

**Step 9. Copy PVS outputs for 3D Slicer quality control**

```
mkdir -p /path/to/project/slicer/sub-001
cp /path/to/project/BIDS_dataset/sub-001/anat/sub-001_T1w.nii.gz \
/path/to/project/slicer/sub-001/
cp /path/to/project/BIDS_dataset/sub-001/anat/sub-001_FLAIR.nii.gz \
/path/to/project/slicer/sub-001/
find /path/to/project/shivai_results -type f -path "*sub-001*" \
\( -iname "*pvs*.nii" -o -iname "*pvs*.nii.gz" \) \
-exec cp {} /path/to/project/slicer/sub-001/ \;
```

**Step 10. Verify files prepared for expert review**

```
ls -lh /path/to/project/slicer/sub-001
```

**Expected input for 3D Slicer**

```
sub-001_T1w.nii.gz
sub-001_FLAIR.nii.gz
SHIVA-PVS PVS segmentation output(s) (*.nii or *.nii.gz)
```

The exported NIfTI files were subsequently imported into 3D Slicer for multiplanar anatomical verification, expert visual quality control, and manual correction of false-positive or incomplete PVS segmentations prior to quantitative analysis.
