# Supplementary material for: Quantitative MRI Characterization of Fluid-Related Phenotypes in Pediatric Epilepsy: Deep Learning-Assisted Assessment of Enlarged Perivascular Space Burden, Regional Distribution, and Clinical Associations
Source: Diagnostics (Basel). 2026 Oct 11;16(20):3304. doi: 10.3390/diagnostics16203304 (PMC13703179; doi:10.3390/diagnostics16203304)
Supplement: Supplementary file 1 [file diagnostics-16-03304-s001.zip › Supplementary Material S2.pdf]

Supplementary Material S2. Command-line implementation of the FreeSurfer-based anatomical segmentation and volumetric analysis pipeline

The following command-line workflow provides the complete implementation used for FreeSurfer-based whole-brain reconstruction, Desikan–Killiany cortical parcellation, automated anatomical segmentation, extraction of individual ventricular CSF and bilateral choroid plexus volumes, extraction of estimated total intracranial volume (eTIV), and export of whole-brain and structure-specific segmentation masks for subsequent visualization and quality control. The workflow assumes that anonymized high-resolution isotropic 3D T1-weighted images have already been converted to NIfTI format before FreeSurfer processing.

Step 1. Launch the FreeSurfer 7.4.1 Docker container

```
docker run -it --rm \
-v /path/to/license.txt:/usr/local/freesurfer/license.txt:ro \
-v /path/to/project_directory:/data \
freesurfer/freesurfer:7.4.1 \
bash
```

Step 2. Define the FreeSurfer subjects directory and subject identifier

```
export SUBJECTS_DIR=/data/subjects
export SUBJECT_ID=subject_001
mkdir -p "$SUBJECTS_DIR"
mkdir -p /data/volumetry/"$SUBJECT_ID"
mkdir -p /data/slicer/"$SUBJECT_ID"
```

Step 3. Verify the input T1-weighted NIfTI image

```
ls -lh /data/nifti/${SUBJECT_ID}_T1.nii.gz
mri_info /data/nifti/${SUBJECT_ID}_T1.nii.gz
```

Step 4. Perform complete whole-brain reconstruction, cortical parcellation, and automated anatomical segmentation

```
recon-all \
-s "$SUBJECT_ID" \
-i /data/nifti/${SUBJECT_ID}_T1.nii.gz \
-all
```

Step 5. Verify successful completion of the FreeSurfer reconstruction

```
tail -n 20 "$SUBJECTS_DIR/$SUBJECT_ID/scripts/recon-all.log"
grep -n "finished without error" "$SUBJECTS_DIR/$SUBJECT_ID/scripts/recon-
all.log" || true
ls -lh "$SUBJECTS_DIR/$SUBJECT_ID/mri/aseg.mgz"
ls -lh "$SUBJECTS_DIR/$SUBJECT_ID/mri/aparc+aseg.mgz"
ls -lh "$SUBJECTS_DIR/$SUBJECT_ID/stats/aseg.stats"
ls -lh "$SUBJECTS_DIR/$SUBJECT_ID/label/lh.aparc.annot"
ls -lh "$SUBJECTS_DIR/$SUBJECT_ID/label/rh.aparc.annot"
```

Step 6. Export the complete whole-brain anatomical volumetric table

```
asegstats2table \
--subjects "$SUBJECT_ID" \
--meas volume \
--delimiter tab \
--tablefile /data/volumetry/${SUBJECT_ID}/${SUBJECT_ID}_aseg_all_volumes.tsv
```

Step 7. Extract the left lateral ventricle (FreeSurfer label 4)

```
mri_binarize \
--i "$SUBJECTS_DIR/$SUBJECT_ID/mri/aseg.mgz" \
--match 4 \
--o /data/slicer/${SUBJECT_ID}/Left_Lateral_Ventricle.mgz
mri_convert \
/data/slicer/${SUBJECT_ID}/Left_Lateral_Ventricle.mgz \
/data/slicer/${SUBJECT_ID}/Left_Lateral_Ventricle.nii.gz
```

Step 8. Extract the right lateral ventricle (FreeSurfer label 43)

```
mri_binarize \
--i "$SUBJECTS_DIR/$SUBJECT_ID/mri/aseg.mgz" \
--match 43 \
--o /data/slicer/${SUBJECT_ID}/Right_Lateral_Ventricle.mgz
mri_convert \
/data/slicer/${SUBJECT_ID}/Right_Lateral_Ventricle.mgz \
/data/slicer/${SUBJECT_ID}/Right_Lateral_Ventricle.nii.gz
```

Step 9. Extract the left inferior lateral ventricle (FreeSurfer label 5)

```
mri_binarize \
--i "$SUBJECTS_DIR/$SUBJECT_ID/mri/aseg.mgz" \
--match 5 \
--o /data/slicer/${SUBJECT_ID}/Left_Inferior_Lateral_Ventricle.mgz
```

```
mri_convert \
/data/slicer/${SUBJECT_ID}/Left_Inferior_Lateral_Ventricle.mgz \
/data/slicer/${SUBJECT_ID}/Left_Inferior_Lateral_Ventricle.nii.gz
```

Step 10. Extract the right inferior lateral ventricle (FreeSurfer label 44)

```
mri_binarize \
--i "$SUBJECTS_DIR/$SUBJECT_ID/mri/aseg.mgz" \
--match 44 \
--o /data/slicer/${SUBJECT_ID}/Right_Inferior_Lateral_Ventricle.mgz
mri_convert \
/data/slicer/${SUBJECT_ID}/Right_Inferior_Lateral_Ventricle.mgz \
/data/slicer/${SUBJECT_ID}/Right_Inferior_Lateral_Ventricle.nii.gz
```

Step 11. Extract the third ventricle (FreeSurfer label 14)

```
mri_binarize \
--i "$SUBJECTS_DIR/$SUBJECT_ID/mri/aseg.mgz" \
--match 14 \
--o /data/slicer/${SUBJECT_ID}/Third_Ventricle.mgz
mri_convert \
/data/slicer/${SUBJECT_ID}/Third_Ventricle.mgz \
/data/slicer/${SUBJECT_ID}/Third_Ventricle.nii.gz
```

Step 12. Extract the fourth ventricle (FreeSurfer label 15)

```
mri_binarize \
--i "$SUBJECTS_DIR/$SUBJECT_ID/mri/aseg.mgz" \
--match 15 \
--o /data/slicer/${SUBJECT_ID}/Fourth_Ventricle.mgz
mri_convert \
/data/slicer/${SUBJECT_ID}/Fourth_Ventricle.mgz \
/data/slicer/${SUBJECT_ID}/Fourth_Ventricle.nii.gz
```

Step 13. Extract the left choroid plexus (FreeSurfer label 31)

```
mri_binarize \
--i "$SUBJECTS_DIR/$SUBJECT_ID/mri/aseg.mgz" \
--match 31 \
--o /data/slicer/${SUBJECT_ID}/Left_Choroid_Plexus.mgz
mri_convert \
/data/slicer/${SUBJECT_ID}/Left_Choroid_Plexus.mgz \
/data/slicer/${SUBJECT_ID}/Left_Choroid_Plexus.nii.gz
```

Step 14. Extract the right choroid plexus (FreeSurfer label 63)

```
mri_binarize \
--i "$SUBJECTS_DIR/$SUBJECT_ID/mri/aseg.mgz" \
--match 63 \
--o /data/slicer/${SUBJECT_ID}/Right_Choroid_Plexus.mgz
mri_convert \
/data/slicer/${SUBJECT_ID}/Right_Choroid_Plexus.mgz \
/data/slicer/${SUBJECT_ID}/Right_Choroid_Plexus.nii.gz
```

Step 15. Export a combined ventricular CSF mask

```
mri_binarize \
--i "$SUBJECTS_DIR/$SUBJECT_ID/mri/aseg.mgz" \
--match 4 43 5 44 14 15 \
--o /data/slicer/${SUBJECT_ID}/Ventricular_CSF_Combined.mgz
mri_convert \
/data/slicer/${SUBJECT_ID}/Ventricular_CSF_Combined.mgz \
/data/slicer/${SUBJECT_ID}/Ventricular_CSF_Combined.nii.gz
```

Step 16. Export a combined bilateral choroid plexus mask

```
mri_binarize \
--i "$SUBJECTS_DIR/$SUBJECT_ID/mri/aseg.mgz" \
--match 31 63 \
--o /data/slicer/${SUBJECT_ID}/Choroid_Plexus_Bilateral.mgz
mri_convert \
/data/slicer/${SUBJECT_ID}/Choroid_Plexus_Bilateral.mgz \
/data/slicer/${SUBJECT_ID}/Choroid_Plexus_Bilateral.nii.gz
```

Step 17. Extract the study-specific ventricular and choroid plexus volumes from aseg.stats

```
grep -E \
"Left-Lateral-Ventricle|Right-Lateral-Ventricle|Left-Inf-Lat-Vent|Right-Inf-Lat-Vent|3rd-Ventricle|4th-Ventricle|Left-choroid-plexus|Right-choroid-plexus" \
"$SUBJECTS_DIR/$SUBJECT_ID/stats/aseg.stats" \
> /data/volumetry/${SUBJECT_ID}/${SUBJECT_ID}_ventricles_choroid_plexus.txt
```

Step 18. Extract estimated total intracranial volume (eTIV)

```
grep "EstimatedTotalIntraCranialVol" \
"$SUBJECTS_DIR/$SUBJECT_ID/stats/aseg.stats" \
> /data/volumetry/${SUBJECT_ID}/${SUBJECT_ID}_eTIV.txt
```

Step 19. Export T1-weighted, whole-brain aseg, and Desikan-Killiany aparc+aseg volumes for visualization

```
mri_convert \
"$SUBJECTS_DIR/$SUBJECT_ID/mri/T1.mgz" \
/data/slicer/${SUBJECT_ID}/T1_FreeSurfer.nii.gz
mri_convert \
"$SUBJECTS_DIR/$SUBJECT_ID/mri/aseg.mgz" \
/data/slicer/${SUBJECT_ID}/aseg.nii.gz
mri_convert \
"$SUBJECTS_DIR/$SUBJECT_ID/mri/aparc+aseg.mgz" \
/data/slicer/${SUBJECT_ID}/aparc_plus_aseg.nii.gz
```

Step 20. Verify all exported structure-specific masks and volumetric outputs

```
ls -lh /data/slicer/${SUBJECT_ID}
ls -lh /data/volumetry/${SUBJECT_ID}
cat
/data/volumetry/${SUBJECT_ID}/${SUBJECT_ID}_ventricles_choroid_plexus.txt
cat /data/volumetry/${SUBJECT_ID}/${SUBJECT_ID}_eTIV.txt
```
