# Supplementary material for: Quantitative MRI Characterization of Fluid-Related Phenotypes in Pediatric Epilepsy: Deep Learning-Assisted Assessment of Enlarged Perivascular Space Burden, Regional Distribution, and Clinical Associations
Source: Diagnostics (Basel). 2026 Oct 11;16(20):3304. doi: 10.3390/diagnostics16203304 (PMC13703179; doi:10.3390/diagnostics16203304)
Supplement: Supplementary file 1 [file diagnostics-16-03304-s001.zip › Supplementary Material S3.pdf]

**Supplementary Material S3. Command-line implementation of FreeSurfer-based hemispheric and regional anatomical mask definition and integration with corrected SHIVA-PVS segmentations**

The following command-line workflow describes the derivation of left and right cerebral hemispheric parenchymal masks and hemisphere-specific basal ganglia (BG) and centrum semiovale (CSO) anatomical masks from FreeSurfer-derived anatomical segmentations, followed by their integration with the final quality-controlled SHIVA-PVS segmentation masks. Cerebral hemispheric masks were defined from hemisphere-specific cerebral cortex, cerebral white matter, and telencephalic subcortical gray-matter labels, excluding ventricular and extra-axial CSF, choroid plexus, corpus callosum, cerebellum, brainstem, and diencephalic structures. The BG compartment was defined separately for each hemisphere by combining the caudate, putamen, and pallidum segmentations, with the thalamus excluded. The CSO compartment was derived separately for each hemisphere from supratentorial cerebral white matter superior to a predefined lateral-ventricular/corpus-callosum reference boundary. The resulting masks were used to partition the final SHIVA-PVS segmentation into left and right hemispheric PVS masks and hemisphere-specific BG-PVS and CSO-PVS masks. The commands below are restricted to hemispheric/regional anatomical mask construction and PVS compartmentalization; subsequent quantitative phenotype extraction was performed separately.

**Step 1. Launch the FreeSurfer 7.4.1 Docker container**

```
docker run -it --rm \
-v /path/to/license.txt:/usr/local/freesurfer/license.txt:ro \
-v /path/to/project_directory:/data \
freesurfer/freesurfer:7.4.1 \
bash
```

**Step 2. Define the FreeSurfer subject and regional-analysis directories**

```
export SUBJECTS_DIR=/data/subjects
export SUBJECT_ID=subject_001
mkdir -p /data/regional_pvs/"$SUBJECT_ID"
```

**Step 3. Verify the FreeSurfer anatomical segmentation and the final corrected SHIVA-PVS mask**

```
ls -lh "$SUBJECTS_DIR/$SUBJECT_ID/mri/aseg.mgz"
ls -lh /data/slicer/${SUBJECT_ID}/${SUBJECT_ID}_PVS_corrected.nii.gz
mri_info "$SUBJECTS_DIR/$SUBJECT_ID/mri/aseg.mgz"
mri_info /data/slicer/${SUBJECT_ID}/${SUBJECT_ID}_PVS_corrected.nii.gz
```

**Step 4. Create the left cerebral hemispheric parenchymal mask**

```
# Included FreeSurfer aseg labels:
# 2 Left-Cerebral-White-Matter; 3 Left-Cerebral-Cortex;
# 11 Left-Caudate; 12 Left-Putamen; 13 Left-Pallidum;
# 17 Left-Hippocampus; 18 Left-Amygdala; 26 Left-Accumbens-area
mri_binarize \
--i "$SUBJECTS_DIR/$SUBJECT_ID/mri/aseg.mgz" \
--match 2 3 11 12 13 17 18 26 \
--o /data/regional_pvs/${SUBJECT_ID}/Left_Hemisphere.mgz
```

**Step 5. Create the right cerebral hemispheric parenchymal mask**

```
# Included FreeSurfer aseg labels:
# 41 Right-Cerebral-White-Matter; 42 Right-Cerebral-Cortex;
# 50 Right-Caudate; 51 Right-Putamen; 52 Right-Pallidum;
# 53 Right-Hippocampus; 54 Right-Amygdala; 58 Right-Accumbens-area
mri_binarize \
--i "$SUBJECTS_DIR/$SUBJECT_ID/mri/aseg.mgz" \
--match 41 42 50 51 52 53 54 58 \
--o /data/regional_pvs/${SUBJECT_ID}/Right_Hemisphere.mgz
```

**Step 6. Create the left BG mask (caudate, putamen, and pallidum; FreeSurfer labels 11, 12, and 13)**

```
mri_binarize \
--i "$SUBJECTS_DIR/$SUBJECT_ID/mri/aseg.mgz" \
--match 11 12 13 \
--o /data/regional_pvs/${SUBJECT_ID}/Left_BG.mgz
```

**Step 7. Create the right BG mask (caudate, putamen, and pallidum; FreeSurfer labels 50, 51, and 52)**

```
mri_binarize \
--i "$SUBJECTS_DIR/$SUBJECT_ID/mri/aseg.mgz" \
--match 50 51 52 \
--o /data/regional_pvs/${SUBJECT_ID}/Right_BG.mgz
```

**Step 8. Create hemisphere-specific cerebral white-matter masks for CSO definition (FreeSurfer labels 2 and 41)**

```
mri_binarize \
```

```
--i "$SUBJECTS_DIR/$SUBJECT_ID/mri/aseg.mgz" \
--match 2 \
--o /data/regional_pvs/${SUBJECT_ID}/Left_Cerebral_WM.mgz
mri_binarize \
--i "$SUBJECTS_DIR/$SUBJECT_ID/mri/aseg.mgz" \
--match 41 \
--o /data/regional_pvs/${SUBJECT_ID}/Right_Cerebral_WM.mgz
```

**Step 9. Create the lateral-ventricular/corpus-callosum reference mask for CSO boundary definition**

```
# FreeSurfer labels 4 and 43: left and right lateral ventricles
# FreeSurfer labels 251-255: corpus-callosum subdivisions
mri_binarize \
--i "$SUBJECTS_DIR/$SUBJECT_ID/mri/aseg.mgz" \
--match 4 43 251 252 253 254 255 \
--o /data/regional_pvs/${SUBJECT_ID}/CSO_Reference.mgz
```

**Step 10. Convert hemispheric, white-matter, and CSO-reference masks to NIfTI format**

```
mri_convert /data/regional_pvs/${SUBJECT_ID}/Left_Hemisphere.mgz \
/data/regional_pvs/${SUBJECT_ID}/Left_Hemisphere.nii.gz
mri_convert /data/regional_pvs/${SUBJECT_ID}/Right_Hemisphere.mgz \
/data/regional_pvs/${SUBJECT_ID}/Right_Hemisphere.nii.gz
mri_convert /data/regional_pvs/${SUBJECT_ID}/Left_Cerebral_WM.mgz \
/data/regional_pvs/${SUBJECT_ID}/Left_Cerebral_WM.nii.gz
mri_convert /data/regional_pvs/${SUBJECT_ID}/Right_Cerebral_WM.mgz \
/data/regional_pvs/${SUBJECT_ID}/Right_Cerebral_WM.nii.gz
mri_convert /data/regional_pvs/${SUBJECT_ID}/CSO_Reference.mgz \
/data/regional_pvs/${SUBJECT_ID}/CSO_Reference.nii.gz
```

**Step 11. Derive left and right CSO masks from cerebral white matter superior to the lateral-ventricular/corpus-callosum reference boundary**

```
python3 - <<'PY'
import os
import nibabel as nib
import numpy as np
subject_id = os.environ['SUBJECT_ID']
base = f'/data/regional_pvs/{subject_id}'
ref_img = nib.load(f'{base}/CSO_Reference.nii.gz')
ref = np.asarray(ref_img.dataobj) > 0
if not np.any(ref):
    raise RuntimeError('CSO reference mask is empty.')
ref_vox = np.argwhere(ref)
ref_ras = nib.affines.apply_affine(ref_img.affine, ref_vox)
superior_cutoff = float(np.max(ref_ras[:, 2]))
for side in ('Left', 'Right'):
    wm_img = nib.load(f'{base}/{side}_Cerebral_WM.nii.gz')
    wm = np.asarray(wm_img.dataobj) > 0
    wm_vox = np.argwhere(wm)
    wm_ras = nib.affines.apply_affine(wm_img.affine, wm_vox)
    keep = wm_ras[:, 2] > superior_cutoff
    cso = np.zeros(wm.shape, dtype=np.uint8)
    kept_vox = wm_vox[keep]
    if kept_vox.size:
        cso[tuple(kept_vox.T)] = 1
    out = nib.Nifti1Image(cso, wm_img.affine, wm_img.header)
    out.set_data_dtype(np.uint8)
    nib.save(out, f'{base}/{side}_CSO.nii.gz')
print(f'CSO inferior boundary (superior extent of lateral-ventricular/callosal reference):
{superior_cutoff:.3f} mm RAS-z')
PY
```

**Step 12. Convert the BG masks to NIfTI format**

```
mri_convert /data/regional_pvs/${SUBJECT_ID}/Left_BG.mgz \
/data/regional_pvs/${SUBJECT_ID}/Left_BG.nii.gz
mri_convert /data/regional_pvs/${SUBJECT_ID}/Right_BG.mgz \
/data/regional_pvs/${SUBJECT_ID}/Right_BG.nii.gz
```

**Step 13. Resample hemispheric and regional anatomical masks to the corrected SHIVA-PVS mask geometry using nearest-neighbor interpolation**

```
for ROI in Left_Hemisphere Right_Hemisphere Left_BG Right_BG Left_CSO Right_CSO; do
mri_vol2vol \
--mov /data/regional_pvs/${SUBJECT_ID}/${ROI}.nii.gz \
```

```
--targ /data/slicer/${SUBJECT_ID}/${SUBJECT_ID}_PVS_corrected.nii.gz \
--regheader \
--interp nearest \
--o /data/regional_pvs/${SUBJECT_ID}/${ROI}_PVSspace.nii.gz
done
```

**Step 14. Verify geometry before voxelwise hemispheric and regional intersection**

```
mri_info /data/slicer/${SUBJECT_ID}/${SUBJECT_ID}_PVS_corrected.nii.gz
mri_info /data/regional_pvs/${SUBJECT_ID}/Left_Hemisphere_PVSspace.nii.gz
mri_info /data/regional_pvs/${SUBJECT_ID}/Right_Hemisphere_PVSspace.nii.gz
mri_info /data/regional_pvs/${SUBJECT_ID}/Left_BG_PVSspace.nii.gz
mri_info /data/regional_pvs/${SUBJECT_ID}/Right_BG_PVSspace.nii.gz
mri_info /data/regional_pvs/${SUBJECT_ID}/Left_CSO_PVSspace.nii.gz
mri_info /data/regional_pvs/${SUBJECT_ID}/Right_CSO_PVSspace.nii.gz
```

**Step 15. Intersect the corrected SHIVA-PVS mask with the hemispheric, BG, and CSO anatomical masks**

```
python3 - <<'PY'
import os
import nibabel as nib
import numpy as np
subject_id = os.environ['SUBJECT_ID']
base = f'/data/regional_pvs/{subject_id}'
pvs_path = f'/data/slicer/{subject_id}/{subject_id}_PVS_corrected.nii.gz'
pvs_img = nib.load(pvs_path)
pvs = np.asarray(pvs_img.dataobj) > 0
for roi in ('Left_Hemisphere', 'Right_Hemisphere',
            'Left_BG', 'Right_BG', 'Left_CSO', 'Right_CSO'):
    roi_img = nib.load(f'{base}/{roi}_PVSspace.nii.gz')
    roi_data = np.asarray(roi_img.dataobj) > 0
    if roi_data.shape != pvs.shape:
        raise RuntimeError(f'Shape mismatch for {roi}.')
    if not np.allclose(roi_img.affine, pvs_img.affine, atol=1e-4):
        raise RuntimeError(f'Affine mismatch for {roi}.')
    compartment_pvs = np.logical_and(pvs, roi_data).astype(np.uint8)
    out = nib.Nifti1Image(compartment_pvs, pvs_img.affine, pvs_img.header)
    out.set_data_dtype(np.uint8)
    nib.save(out, f'{base}/{roi}_PVS.nii.gz')
PY
```

**Step 16. Create bilateral BG-PVS and CSO-PVS masks**

```
python3 - <<'PY'
import os
import nibabel as nib
import numpy as np
subject_id = os.environ['SUBJECT_ID']
base = f'/data/regional_pvs/{subject_id}'
for region in ('BG', 'CSO'):
    left_img = nib.load(f'{base}/Left_{region}_PVS.nii.gz')
    right_img = nib.load(f'{base}/Right_{region}_PVS.nii.gz')
    left = np.asarray(left_img.dataobj) > 0
    right = np.asarray(right_img.dataobj) > 0
    if left.shape != right.shape:
        raise RuntimeError(f'Left/right shape mismatch for {region}.')
    if not np.allclose(left_img.affine, right_img.affine, atol=1e-4):
        raise RuntimeError(f'Left/right affine mismatch for {region}.')
    combined = np.logical_or(left, right).astype(np.uint8)
    out = nib.Nifti1Image(combined, left_img.affine, left_img.header)
    out.set_data_dtype(np.uint8)
    nib.save(out, f'{base}/Bilateral_{region}_PVS.nii.gz')
PY
```

**Step 17. Verify all hemispheric and regional PVS masks**

```
ls -lh /data/regional_pvs/${SUBJECT_ID}
mri_info /data/regional_pvs/${SUBJECT_ID}/Left_Hemisphere_PVS.nii.gz
mri_info /data/regional_pvs/${SUBJECT_ID}/Right_Hemisphere_PVS.nii.gz
mri_info /data/regional_pvs/${SUBJECT_ID}/Left_BG_PVS.nii.gz
mri_info /data/regional_pvs/${SUBJECT_ID}/Right_BG_PVS.nii.gz
mri_info /data/regional_pvs/${SUBJECT_ID}/Left_CSO_PVS.nii.gz
mri_info /data/regional_pvs/${SUBJECT_ID}/Right_CSO_PVS.nii.gz
mri_info /data/regional_pvs/${SUBJECT_ID}/Bilateral_BG_PVS.nii.gz
```

```
mri_info /data/regional_pvs/${SUBJECT_ID}/Bilateral_CSO_PVS.nii.gz
```
