# Supplementary material for: Quantitative MRI Characterization of Fluid-Related Phenotypes in Pediatric Epilepsy: Deep Learning-Assisted Assessment of Enlarged Perivascular Space Burden, Regional Distribution, and Clinical Associations
Source: Diagnostics (Basel). 2026 Oct 11;16(20):3304. doi: 10.3390/diagnostics16203304 (PMC13703179; doi:10.3390/diagnostics16203304)
Supplement: Supplementary file 1 [file diagnostics-16-03304-s001.zip › Supplementary Material S4.pdf]

Supplementary Material S4

Statistical Code

Code for Independent Reproduction of the Benjamini–Hochberg FDR Adjustments

```
# ==================================================================
# REPRODUCIBLE BENJAMINI-HOCHBERG FDR ADJUSTMENT WORKFLOW
# Primary exploratory clinical-association analysis and expanded
# post-selection sensitivity analysis
# ==================================================================

# R version: 4.3.3
#
# This code independently reproduces the Benjamini-Hochberg
# false discovery rate (FDR) adjustments applied to the two
# exploratory clinical-association analysis families reported in
# Supplementary Tables S6-S8.
#
# The raw P values below are entered at the full computational
# precision reported in Supplementary Table S8 and in the same
# analytical order.
#
# Family 1:
#   Primary exploratory clinical-association analysis
#   64 inferential tests (P01-P64)
#
# Family 2:
#   Expanded post-selection sensitivity analysis
#   114 inferential tests (S001-S114)
#
# For both families, category-specific estimated marginal means
# (EMMs) do not constitute additional inferential tests.
# Post hoc pairwise EMM contrasts are not included in either
# Benjamini-Hochberg family; where applicable, these contrasts
# were adjusted separately using the Tukey procedure.
#
# No additional R packages are required for the FDR calculations.


# ==================================================================
# 1. PRIMARY EXPLORATORY CLINICAL-ASSOCIATION FAMILY
# ==================================================================

# Full-precision raw P values corresponding to P01-P64 in
# Supplementary Table S8, Panel B.

p_primary_64 <- c(
  0.0891746,    # P01
  0.0996842,    # P02
  0.1096387,    # P03
  0.0096841,    # P04
  0.0117426,    # P05
  0.0087315,    # P06
  0.0176934,    # P07
  0.0396172,    # P08
  0.080538,     # P09
  0.090584,     # P10
  0.00007997,   # P11
  0.0001686,    # P12
  0.0003864,    # P13
  0.00000362,   # P14
  0.00001158,   # P15
  0.000000058,  # P16
  0.00000835,   # P17
  0.0001755,    # P18
  0.000167,     # P19
  0.001987,     # P20
  0.0118214,    # P21
```

```
  0.0296417,    # P22
  0.0302146,    # P23
  0.0009187,    # P24
  0.0018743,    # P25
  0.0007862,    # P26
  0.0048127,    # P27
  0.0297843,    # P28
  0.015123,     # P29
  0.040628,     # P30
  0.0009462,    # P31
  0.0018427,    # P32
  0.0027314,    # P33
  0.00000011,   # P34
  0.00000031,   # P35
  0.000000006,  # P36
  0.0018635,    # P37
  0.0039421,    # P38
  0.004612,     # P39
  0.006285,     # P40
  0.0398627,    # P41
  0.0401735,    # P42
  0.0496814,    # P43
  0.0022148,    # P44
  0.0007416,    # P45
  0.0001843,    # P46
  0.0057318,    # P47
  0.0395826,    # P48
  0.025238,     # P49
  0.051639,     # P50
  0.271218,     # P51
  0.148912,     # P52
  0.158764,     # P53
  0.048327,     # P54
  0.044783,     # P55
  0.018947,     # P56
  0.028816,     # P57
  0.007386,     # P58
  0.012437,     # P59
  0.004817,     # P60
  0.009846,     # P61
  0.009846,     # P62
  0.250684,     # P63
  0.099217      # P64
)

# Verify the size of the FDR family.

stopifnot(length(p_primary_64) == 64L)


# ==================================================================
# 2. BENJAMINI-HOCHBERG ADJUSTMENT FOR THE 64-TEST FAMILY
# ==================================================================

q_primary_64 <- p.adjust(
  p_primary_64,
  method = "BH"
)

primary_64_results <- data.frame(
  Test_ID = sprintf("P%02d", seq_along(p_primary_64)),
  Raw_P = p_primary_64,
  FDR_q = q_primary_64,
  FDR_significant = q_primary_64 < 0.05,
  stringsAsFactors = FALSE
)

print(
```

```r
  primary_64_results,
  row.names = FALSE,
  digits = 10
)


# ===================================================================
# 3. EXPANDED POST-SELECTION SENSITIVITY-ANALYSIS FAMILY
# ===================================================================

# Full-precision raw P values corresponding to S001-S114 in
# Supplementary Table S8, Panel C.
#
# The preceding significance-based EPVS outcome-selection
# criterion used for the primary clinical-association analysis
# was removed in this sensitivity analysis. The corresponding
# clinical associations were evaluated across the expanded
# 18-measure EPVS outcome set while otherwise retaining the
# predictor-specific analytical architecture.

p_expanded_114 <- c(
  0.0891746,     # S001
  0.0996842,     # S002
  0.1096387,     # S003
  0.4196721,     # S004
  0.3798463,     # S005
  0.3997318,     # S006
  0.0096841,     # S007
  0.0117426,     # S008
  0.0087315,     # S009
  0.6097284,     # S010
  0.7396142,     # S011
  0.2897816,     # S012
  0.0196847,     # S013
  0.0217463,     # S014
  0.0176934,     # S015
  0.0396172,     # S016
  0.0805380,     # S017
  0.0905840,     # S018

  0.00007997,    # S019
  0.0001686,     # S020
  0.0003864,     # S021
  0.3097421,     # S022
  0.2798164,     # S023
  0.2996847,     # S024
  0.00000362,    # S025
  0.00001158,    # S026
  0.000000058,   # S027
  0.4597281,     # S028
  0.5796134,     # S029
  0.1897426,     # S030
  0.00000835,    # S031
  0.00001142,    # S032
  0.00000835,    # S033
  0.0001755,     # S034
  0.0001670,     # S035
  0.0019870,     # S036

  0.0118214,     # S037
  0.0296417,     # S038
  0.0302146,     # S039
  0.3396842,     # S040
  0.3097316,     # S041
  0.3196475,     # S042
  0.0009187,     # S043
  0.0018743,     # S044
  0.0007862,     # S045
```

```
0.6697143,      # S046
0.7296385,      # S047
0.2197834,      # S048
0.0068124,      # S049
0.0087641,      # S050
0.0048127,      # S051
0.0297843,      # S052
0.0151230,      # S053
0.0406280,      # S054

0.0009462,      # S055
0.0018427,      # S056
0.0027314,      # S057
0.2896417,      # S058
0.2497836,      # S059
0.2697184,      # S060
0.00000011,     # S061
0.00000031,     # S062
0.000000006,    # S063
0.5396812,      # S064
0.6197425,      # S065
0.1697843,      # S066
0.0027318,      # S067
0.0039421,      # S068
0.0018635,      # S069
0.0039421,      # S070
0.0046120,      # S071
0.0062850,      # S072

0.0398627,      # S073
0.0401735,      # S074
0.0496814,      # S075
0.3597284,      # S076
0.3296817,      # S077
0.3497421,      # S078
0.0022148,      # S079
0.0007416,      # S080
0.0001843,      # S081
0.7096384,      # S082
0.7896417,      # S083
0.2597835,      # S084
0.0087642,      # S085
0.0107316,      # S086
0.0057318,      # S087
0.0395826,      # S088
0.0252380,      # S089
0.0516390,      # S090

0.271218,       # S091
0.148912,       # S092
0.158764,       # S093
0.048327,       # S094

0.044783,       # S095
0.018947,       # S096
0.028816,       # S097
0.309684,       # S098
0.279731,       # S099
0.299642,       # S100
0.007386,       # S101
0.012437,       # S102
0.004817,       # S103
0.409728,       # S104
0.519684,       # S105
0.159731,       # S106
0.013742,       # S107
0.015684,       # S108
0.009846,       # S109
```

```
  0.009846,      # S110
  0.079684,      # S111
  0.089731,      # S112

  0.250684,      # S113
  0.099217       # S114
)

# Verify the size of the expanded FDR family.

stopifnot(length(p_expanded_114) == 114L)


# =================================================================
# 4. BENJAMINI-HOCHBERG ADJUSTMENT FOR THE 114-TEST FAMILY
# =================================================================

q_expanded_114 <- p.adjust(
  p_expanded_114,
  method = "BH"
)

expanded_114_results <- data.frame(
  Test_ID = sprintf("S%03d", seq_along(p_expanded_114)),
  Raw_P = p_expanded_114,
  FDR_q = q_expanded_114,
  FDR_significant = q_expanded_114 < 0.05,
  stringsAsFactors = FALSE
)

print(
  expanded_114_results,
  row.names = FALSE,
  digits = 10
)


# =================================================================
# 5. REPRODUCIBILITY AND INTEGRITY CHECKS
# =================================================================

# Confirm that both FDR families contain the intended number of
# inferential tests and that all raw and adjusted P values are
# within the valid probability range.

stopifnot(
  length(p_primary_64) == 64L,
  length(q_primary_64) == 64L,
  length(p_expanded_114) == 114L,
  length(q_expanded_114) == 114L,
  all(is.finite(p_primary_64)),
  all(is.finite(q_primary_64)),
  all(is.finite(p_expanded_114)),
  all(is.finite(q_expanded_114)),
  all(p_primary_64 >= 0 & p_primary_64 <= 1),
  all(q_primary_64 >= 0 & q_primary_64 <= 1),
  all(p_expanded_114 >= 0 & p_expanded_114 <= 1),
  all(q_expanded_114 >= 0 & q_expanded_114 <= 1)
)


# =================================================================
# 6. OPTIONAL PRESENTATION-ROUNDING CHECK
# =================================================================

# P and q values reported in the manuscript and supplementary
# tables were rounded for presentation. The calculations above
# use the full-precision raw P-value vectors reported in
```

```
# Supplementary Table S8.
#
# The following commands display the reproduced FDR-adjusted
# q values rounded to three decimal places for comparison with
# presentation-rounded values in Supplementary Tables S6 and S7.

primary_64_results$FDR_q_rounded <-
  round(primary_64_results$FDR_q, 3)

expanded_114_results$FDR_q_rounded <-
  round(expanded_114_results$FDR_q, 3)

print(
  primary_64_results[
    c("Test_ID", "Raw_P", "FDR_q", "FDR_q_rounded")
  ],
  row.names = FALSE,
  digits = 10
)

print(
  expanded_114_results[
    c("Test_ID", "Raw_P", "FDR_q", "FDR_q_rounded")
  ],
  row.names = FALSE,
  digits = 10
)
```
