# Supplementary material for: Quantitative MRI Characterization of Fluid-Related Phenotypes in Pediatric Epilepsy: Deep Learning-Assisted Assessment of Enlarged Perivascular Space Burden, Regional Distribution, and Clinical Associations
Source: Diagnostics (Basel). 2026 Oct 11;16(20):3304. doi: 10.3390/diagnostics16203304 (PMC13703179; doi:10.3390/diagnostics16203304)
Supplement: Supplementary file 1 [file diagnostics-16-03304-s001.zip › Supplementary Table S1.pdf]

Supplementary Table S1. Manual Correction Burden and Automated-to-Corrected Mask Agreement of SHIVA-PVS Segmentations

**Analysis domain**	**Measure**	**Epilepsy group (n = 123)**	**Control group (n = 108)**	**Between-group *P value***	**Overall agreement**
Manual correction burden	Any voxel-level modification, n (%)	68 (55.3)	56 (51.9)	0.60[a]	—
	Correction magnitude, %, median (IQR)	5.2 (3.1–7.6)	4.9 (2.9–7.1)	0.45[b]	—
Automated-to-corrected volumetric agreement	Total EPVS volume, ICC (95% CI)	—	—	—	0.91 (0.89–0.93)[c]
Automated-to-corrected spatial overlap	Whole-brain EPVS, DSC	—	—	—	0.82[d]
	Bilateral CSO-EPVS, DSC	—	—	—	0.78[d]
	Bilateral BG-EPVS, DSC	—	—	—	0.72[d]

Table note. Manual correction burden was assessed by comparing the initial automated SHIVA-PVS masks with the final masks obtained after expert quality control and manual correction. Correction frequency was defined as the presence of any voxel-level difference between the initial automated and final corrected masks; therefore, this measure indicates whether any modification occurred, irrespective of its magnitude. Correction magnitude was quantified as the voxelwise symmetric difference between the initial automated mask (A) and final corrected mask (C), normalized to the initial automated mask volume and expressed as a percentage $[100 \times |A \Delta C|/|A|]$, thereby accounting for both added and removed voxels. [a]Between-group differences in correction frequency were evaluated using the Pearson chi-square test. [b]Between-group differences in correction magnitude were evaluated using the Mann–Whitney U test. [c]Volumetric agreement between the initial automated and final corrected total EPVS masks was evaluated using a two-way mixed-effects, absolute-agreement, single-measure intraclass correlation coefficient (ICC) with 95% confidence intervals (CIs). [d]Voxelwise spatial overlap between the initial automated and final corrected masks was assessed using the Dice similarity coefficient (DSC) for whole-brain EPVS, bilateral centrum semiovale EPVS (CSO-EPVS), and bilateral basal ganglia EPVS (BG-EPVS) masks. ICC and DSC values characterize agreement between the initial automated and final expert-reviewed masks and should not be interpreted as estimates of segmentation accuracy or as validation against an independently delineated manual reference standard.

Abbreviations: BG, basal ganglia; CI, confidence interval; CSO, centrum semiovale; DSC, Dice similarity coefficient; EPVS, enlarged perivascular spaces; ICC, intraclass correlation coefficient; IQR, interquartile range; SHIVA-PVS, SHIVA perivascular space segmentation model.
