# Supplementary material for: Quantitative MRI Characterization of Fluid-Related Phenotypes in Pediatric Epilepsy: Deep Learning-Assisted Assessment of Enlarged Perivascular Space Burden, Regional Distribution, and Clinical Associations
Source: Diagnostics (Basel). 2026 Oct 11;16(20):3304. doi: 10.3390/diagnostics16203304 (PMC13703179; doi:10.3390/diagnostics16203304)
Supplement: Supplementary file 1 [file diagnostics-16-03304-s001.zip › Supplementary Table S2.pdf]

Supplementary Table S2. Sensitivity Analysis of EPVS Phenotypes Using Initial Unedited SHIVA-PVS Masks Compared With the Primary Expert-Corrected Masks

**EPVS phenotype**	**Primary corrected masks, adjusted difference (95% CI)**	***P value***	**FDR-adjusted *q value***	**Initial unedited masks, adjusted difference (95% CI)**	***P value***	**FDR-adjusted *q value***
Global and hemispheric EPVS burden						
Total EPVS volume, mm^3	284.7 (105.9–463.5)	0.001	0.003	240.0 (77.0–403.0)	0.0041	0.009
Right hemispheric EPVS volume, mm^3	144.6 (47.8–241.4)	0.003	0.005	122.0 (34.0–210.0)	0.007	0.013
Left hemispheric EPVS volume, mm^3	140.1 (43.2–237.0)	0.004	0.007	118.0 (29.0–207.0)	0.009	0.015
Regional EPVS burden						
Right CSO-EPVS volume, mm^3	19.6 (−27.2–66.4)	0.412	0.432	17.0 (−27.0–61.0)	0.450	0.470
Left CSO-EPVS volume, mm^3	20.8 (−24.2–65.8)	0.365	0.402	18.0 (−24.0–60.0)	0.400	0.440
Total CSO-EPVS volume, mm^3	40.4 (−42.2–123.0)	0.338	0.391	35.0 (−42.0–112.0)	0.370	0.430
Right BG-EPVS volume, mm^3	84.5 (43.2–125.8)	<0.001	<0.001	72.0 (34.0–110.0)	<0.001	<0.001
Left BG-EPVS volume, mm^3	76.4 (38.2–114.6)	<0.001	<0.001	65.0 (30.0–100.0)	<0.001	<0.001
Total BG-EPVS volume, mm^3	160.9 (89.5–232.3)	<0.001	<0.001	137.0 (72.0–202.0)	<0.001	<0.001
EPVS asymmetry						
Hemispheric EPVS asymmetry index	0.004 (−0.003–0.011)	0.284	0.347	0.004 (−0.004–0.012)	0.310	0.360
CSO-EPVS asymmetry index	0.002 (−0.006–0.010)	0.611	0.611	0.002 (−0.006–0.010)	0.650	0.650
BG-EPVS asymmetry index	0.013 (−0.001–0.027)	0.073	0.107	0.012 (−0.002–0.026)	0.090	0.120
Relative						

regional EPVS burden						
Right CSO relative EPVS burden	−0.074 (−0.117 to −0.031)	<0.001	0.001	−0.063 (−0.104 to −0.022)	0.002	0.004
Left CSO relative EPVS burden	−0.074 (−0.118 to −0.031)	<0.001	0.001	−0.063 (−0.105 to −0.021)	0.002	0.004
Right BG relative EPVS burden	0.060 (0.032–0.088)	<0.001	<0.001	0.052 (0.026–0.078)	<0.001	0.002
Left BG relative EPVS burden	0.055 (0.029–0.082)	<0.001	<0.001	0.047 (0.022–0.072)	<0.001	0.002
Total CSO relative EPVS burden	−0.074 (−0.115 to −0.033)	<0.001	<0.001	−0.063 (−0.102 to −0.024)	0.001	0.003
Total BG relative EPVS burden	0.058 (0.032–0.083)	<0.001	<0.001	0.050 (0.026–0.074)	<0.001	0.002
Right hemispheric relative EPVS burden	−0.0002 (−0.0006–0.0002)	0.284	0.347	−0.0002 (−0.0006–0.0002)	0.310	0.360
Left hemispheric relative EPVS burden	0.0002 (−0.0002–0.0006)	0.284	0.347	0.0002 (−0.0002–0.0006)	0.310	0.360
Relative CSO-to-BG distribution						
Right CSO-to-BG EPVS volume ratio	−1.948 (−2.843 to −1.053)	<0.001	<0.001	−1.66 (−2.49 to −0.83)	<0.001	0.002
Left CSO-to-BG EPVS volume ratio	−1.957 (−2.857 to −1.058)	<0.001	<0.001	−1.67 (−2.51 to −0.83)	<0.001	0.002

Table note. Data for the primary analysis represent adjusted between-group differences derived from the final expert-reviewed and corrected SHIVA-PVS masks, whereas the sensitivity analysis was repeated using the initial unedited SHIVA-PVS masks. Adjusted differences were calculated as epilepsy minus control and are presented with corresponding 95% confidence intervals (CIs). The same outcome definitions, statistical models, covariate structure, and multiple-comparison framework used in the primary EPVS phenotype analysis were retained for the unedited-mask sensitivity analysis. All models were adjusted for age and sex; models evaluating absolute EPVS volumes were additionally adjusted for estimated total intracranial volume (eTIV). Absolute EPVS volumes are expressed in mm^3 and were adjusted for eTIV as a covariate rather than normalized to eTIV. eTIV was not included in models evaluating asymmetry indices or dimensionless relative-distribution measures. Relative regional burden measures represent the proportional distribution of EPVS burden across the predefined anatomical compartments. Asymmetry indices, relative regional burden measures, and CSO-to-BG ratios are dimensionless. Reported P values are

two-sided, unadjusted values for the group effect. Within each mask-based analysis, all quantitative EPVS phenotype comparisons were treated as a single hypothesis family and adjusted using the Benjamini–Hochberg false discovery rate (FDR) procedure; corresponding FDR-adjusted q values are reported, with $q < 0.05$ considered statistically significant. The unedited-mask analysis was performed as a sensitivity analysis to assess whether the principal epilepsy–control EPVS findings were sensitive to the expert manual-correction step. It was not intended as, and should not be interpreted as, an assessment of segmentation accuracy or validation against an independently delineated manual reference standard.

Abbreviations: BG, basal ganglia; CI, confidence interval; CSO, centrum semiovale; EPVS, enlarged perivascular spaces; FDR, false discovery rate; eTIV, estimated total intracranial volume; SHIVA-PVS, SHIVA perivascular space segmentation model.
