# Supplementary material for: Quantitative MRI Characterization of Fluid-Related Phenotypes in Pediatric Epilepsy: Deep Learning-Assisted Assessment of Enlarged Perivascular Space Burden, Regional Distribution, and Clinical Associations
Source: Diagnostics (Basel). 2026 Oct 11;16(20):3304. doi: 10.3390/diagnostics16203304 (PMC13703179; doi:10.3390/diagnostics16203304)
Supplement: Supplementary file 1 [file diagnostics-16-03304-s001.zip › Supplementary Table S3.pdf]

Supplementary Table S3. Probability-Threshold Sensitivity Analysis of EPVS Phenotypes

**EPVS phenotype**	**Threshold 0.40 Adjusted difference (95% CI)**	***P* value**	***q* value**	**Primary threshold 0.50 Adjusted difference (95% CI)**	***P* value**	***q* value**	**Threshold 0.60 Adjusted difference (95% CI)**	***P* value**	***q* value**
Global and hemispheric EPVS burden									
Total EPVS volume, mm³	306 (118–494)	0.0014	0.004	284.7 (105.9–463.5)	0.001	0.003	262 (91–433)	0.0032	0.007
Right hemispheric EPVS volume, mm³	156 (54–258)	0.0027	0.006	144.6 (47.8–241.4)	0.003	0.005	133 (41–225)	0.0048	0.009
Left hemispheric EPVS volume, mm³	150 (49–251)	0.0036	0.007	140.1 (43.2–237.0)	0.004	0.007	129 (36–222)	0.0066	0.011
Regional EPVS burden									
Right CSO-EPVS volume, mm³	22 (−28–72)	0.386	0.425	19.6 (−27.2–66.4)	0.412	0.432	18 (−26–62)	0.421	0.447
Left CSO-EPVS volume, mm³	23 (−25–71)	0.348	0.401	20.8 (−24.2–65.8)	0.365	0.402	19 (−23–61)	0.377	0.423
Total CSO-EPVS volume, mm³	45 (−43–133)	0.316	0.386	40.4 (−42.2–123.0)	0.338	0.391	37 (−40–114)	0.349	0.407
Right BG-EPVS volume, mm³	89(46–132)	<0.001	<0.001	84.5 (43.2–125.8)	<0.001	<0.001	78 (39–117)	<0.001	<0.001
Left BG-EPVS volume, mm³	81 (41–121)	<0.001	<0.001	76.4 (38.2–114.6)	<0.001	<0.001	70 (34–106)	<0.001	<0.001

Total BG-EPVS volume, mm^3	170 (95–245)	<0.001	<0.001	160.9 (89.5–232.3)	<0.001	<0.001	148 (80–216)	<0.001	<0.001
EPVS asymmetry									
Hemispheric EPVS asymmetry index	0.004 (−0.003–0.011)	0.27	0.34	0.004 (−0.003–0.011)	0.284	0.347	0.004 (−0.004–0.012)	0.30	0.36
CSO-EPVS asymmetry index	0.002 (−0.006–0.010)	0.59	0.61	0.002 (−0.006–0.010)	0.611	0.611	0.002 (−0.006–0.010)	0.63	0.65
BG-EPVS asymmetry index	0.014 (0.000–0.028)	0.055	0.083	0.013 (−0.001–0.027)	0.073	0.107	0.012 (−0.002–0.026)	0.092	0.126
Relative regional EPVS burden									
Right CSO relative EPVS burden	−0.078 (−0.122 to −0.034)	<0.001	0.001	−0.074 (−0.117 to −0.031)	<0.001	0.001	−0.068 (−0.109 to −0.027)	0.001	0.003
Left CSO relative EPVS burden	−0.078 (−0.123 to −0.033)	<0.001	0.001	−0.074 (−0.118 to −0.031)	<0.001	0.001	−0.068 (−0.110 to −0.026)	0.002	0.004
Right BG relative EPVS burden	0.063 (0.034–0.092)	<0.001	<0.001	0.060 (0.032–0.088)	<0.001	<0.001	0.055 (0.029–0.081)	<0.001	0.002
Left BG relative EPVS burden	0.058 (0.030–0.086)	<0.001	<0.001	0.055 (0.029–0.082)	<0.001	<0.001	0.051 (0.026–0.076)	<0.001	0.002
Total CSO relative EPVS burden	−0.078 (−0.120 to −0.036)	<0.001	<0.001	−0.074 (−0.115 to −0.033)	<0.001	<0.001	−0.068 (−0.107 to −0.029)	0.001	0.003
Total BG relative EPVS burden	0.061 (0.035–0.087)	<0.001	<0.001	0.058 (0.032–0.083)	<0.001	<0.001	0.053 (0.029–0.077)	<0.001	0.002
Right hemispheric relative EPVS burden	−0.0002 (−0.0006–0.0002)	0.27	0.34	−0.0002 (−0.0006 –0.0002)	0.284	0.347	−0.0002 (−0.0006–0.0002)	0.30	0.36
Left	0.0002	0.27	0.34	0.0002	0.284	0.347	0.0002	0.30	0.36

hemispheric relative EPVS burden	(−0.0002–0.0006)			(−0.0002–0.0006)			(−0.0002–0.0006)		
Relative CSO-to-BG distribution									
Right CSO-to-BG EPVS volume ratio	−2.08 (−3.02 to −1.14)	<0.001	<0.001	−1.948 (−2.843 to −1.053)	<0.001	<0.001	−1.79 (−2.63 to −0.95)	<0.001	<0.001
Left CSO-to-BG EPVS volume ratio	−2.09 (−3.04 to −1.14)	<0.001	<0.001	−1.957 (−2.857 to −1.058)	<0.001	<0.001	−1.80 (−2.65 to −0.95)	<0.001	<0.001

Table note. Probability-threshold sensitivity analyses were performed using the retained SHIVA-PVS probability maps to evaluate the robustness of the principal EPVS phenotype findings to the binarization threshold applied to the model output. In addition to the primary probability threshold of 0.50, the corresponding EPVS analyses were repeated using a more permissive threshold of 0.40 and a more conservative threshold of 0.60, symmetrically positioned around the primary threshold. For each threshold, probability maps were binarized using the specified cutoff, and the resulting EPVS masks were processed using the same anatomical regionalization and quantitative phenotype definitions as in the primary analysis. The same statistical models, covariate structure, and multiple-comparison framework used for the corresponding primary EPVS analyses were applied at each threshold. Adjusted differences were calculated as epilepsy minus control and are presented with corresponding 95% confidence intervals (CIs). All models were adjusted for age and sex; models evaluating absolute EPVS volumes were additionally adjusted for estimated total intracranial volume (eTIV). Absolute EPVS volumes are expressed in mm^3 and were adjusted for eTIV as a covariate rather than normalized to eTIV. eTIV was not included in models evaluating asymmetry indices or dimensionless relative-distribution measures. Relative regional burden measures represent the proportional distribution of EPVS burden across the predefined anatomical compartments. Asymmetry indices, relative regional burden measures, and CSO-to-BG ratios are dimensionless. Reported P values are two-sided, unadjusted values for the group effect. At each probability threshold, all quantitative EPVS phenotype comparisons were treated as a single hypothesis family and adjusted using the Benjamini–Hochberg false discovery rate (FDR) procedure; corresponding FDR-adjusted q values are reported, with $q < 0.05$ considered statistically significant. The alternative thresholds were evaluated as sensitivity analyses and should not be interpreted as independently validated, optimized, or diagnostically derived segmentation thresholds.

Abbreviations: BG, basal ganglia; CI, confidence interval; CSO, centrum semiovale; EPVS, enlarged perivascular spaces; FDR, false discovery rate; eTIV, estimated total intracranial volume; SHIVA-PVS, SHIVA perivascular space segmentation model.
