# Supplementary material for: Quantitative MRI Characterization of Fluid-Related Phenotypes in Pediatric Epilepsy: Deep Learning-Assisted Assessment of Enlarged Perivascular Space Burden, Regional Distribution, and Clinical Associations
Source: Diagnostics (Basel). 2026 Oct 11;16(20):3304. doi: 10.3390/diagnostics16203304 (PMC13703179; doi:10.3390/diagnostics16203304)
Supplement: Supplementary file 1 [file diagnostics-16-03304-s001.zip › Supplementary Table S4.pdf]

Supplementary Table S4. Model Diagnostics, Adjusted Standardized Effect Sizes, and Robustness Analyses for Between-Group EPVS Phenotype Comparisons

EPVS phenotype measure	Adjusted standardized effect, d_adj (95% CI)	Breusch–Pagan P value	Maximum Cook's D	HC3-adjusted P value	Bootstrap 95% CI for adjusted group difference	Diagnostic assessment	Robustness conclusion
Global and hemispheric EPVS burden							
Total EPVS volume	0.42 (0.16 to 0.68)	.183	.053	.002	112.7 to 462.9 mm³	No material assumption departure	Concordant
Right hemispheric EPVS volume	0.39 (0.13 to 0.65)	.223	.047	.004	48.7 to 241.3 mm³	No material assumption departure	Concordant
Left hemispheric EPVS volume	0.38 (0.12 to 0.64)	.163	.051	.005	44.3 to 236.7 mm³	No material assumption departure	Concordant
Regional EPVS burden							
Right CSO-EPVS volume	0.13 (−0.17 to 0.43)	.313	.041	.421	−29.3 to 67.7 mm³	No material assumption departure	Concordant; inference remained nonsignificant
Left CSO-EPVS volume	0.13 (−0.15 to 0.41)	.273	.044	.374	−25.7 to 67.1 mm³	No material assumption departure	Concordant; inference remained nonsignificant
Total CSO-EPVS volume	0.13 (−0.13 to 0.39)	.253	.046	.347	−43.9 to 125.3 mm³	No material assumption departure	Concordant; inference remained nonsignificant
Right BG-EPVS volume	0.75 (0.38 to 1.12)	.073	.079	<.001	42.3 to 127.7 mm³	Mild residual-tail departure on visual inspection; BP test nonsignificant	Concordant
Left BG-EPVS volume	0.71 (0.36 to 1.06)	.093	.074	<.001	37.3 to 116.1 mm³	Mild residual-tail departure on visual inspection; BP test nonsignificant	Concordant
Total BG-EPVS volume	0.77 (0.43 to 1.11)	.043	.087	<.001	89.7 to 234.3 mm³	Evidence of mild heteroscedasticity	Concordant
EPVS asymmetry							
Hemispheric EPVS	0.12 (−0.09 to 0.33)	.343	.049	.291	−0.0031 to 0.0113	No material assumption	Concordant; inference

asymmetry index						departure	remained nonsignificant
CSO-EPVS asymmetry index	0.06 (−0.17 to 0.29)	.413	.045	.618	−0.0063 to 0.0107	No material assumption departure	Concordant; inference remained nonsignificant
BG-EPVS asymmetry index	0.23 (−0.02 to 0.48)	.123	.063	.087	−0.0021 to 0.0283	Mild residual-tail departure on visual inspection; BP test nonsignificant	Concordant; inference remained nonsignificant
Relative regional EPVS burden							
Right CSO relative EPVS burden	−0.51 (−0.79 to −0.23)	.083	.061	.001	−0.1193 to −0.0297	Mild residual-pattern departure; BP test nonsignificant	Concordant
Left CSO relative EPVS burden	−0.49 (−0.78 to −0.21)	.103	.058	.001	−0.1203 to −0.0287	Mild residual-tail departure on visual inspection; BP test nonsignificant	Concordant
Right BG relative EPVS burden	0.61 (0.33 to 0.89)	.053	.071	<.001	0.0313 to 0.0891	Borderline evidence of heteroscedasticity	Concordant
Left BG relative EPVS burden	0.59 (0.31 to 0.87)	.073	.068	<.001	0.0283 to 0.0837	Mild residual-pattern departure; BP test nonsignificant	Concordant
Total CSO relative EPVS burden	−0.53 (−0.82 to −0.24)	.063	.067	<.001	−0.1173 to −0.0317	Mild residual-pattern departure; BP test nonsignificant	Concordant
Total BG relative EPVS burden	0.63 (0.35 to 0.91)	.043	.077	<.001	0.0317 to 0.0853	Evidence of mild heteroscedasticity	Concordant
Right hemispheric relative EPVS burden	−0.14 (−0.39 to 0.11)	.293	.048	.291	−0.00061 to 0.00023	No material assumption departure	Concordant; inference remained nonsignificant
Left hemispheric relative EPVS burden	0.14 (−0.11 to 0.39)	.293	.048	.291	−0.00023 to 0.00061	No material assumption departure	Concordant; inference remained nonsignificant
Relative CSO-to-BG distribution							
Right CSO-to-BG EPVS	−0.65 (−0.95 to −0.35)	.003	.129	<.001	−2.93 to −1.01	Clear evidence of heteroscedastici	Concordant after HC3, bootstrap,

volume ratio						ty and right-tail departure	and log-ratio sensitivity analyses
Left CSO-to-BG EPVS volume ratio	−0.63 (−0.93 to −0.33)	.006	.117	<.001	−2.91 to −1.03	Clear evidence of heteroscedasticity and right-tail departure	Concordant after HC3, bootstrap, and log-ratio sensitivity analyses

Table note. Model diagnostics were evaluated for all EPVS outcomes included in the between-group analyses reported in Table 5, irrespective of statistical significance. Original-scale general linear models were retained as the principal models to preserve the interpretability of adjusted estimated marginal means and between-group differences. All models included diagnostic group, age, and sex; models of absolute EPVS volumes additionally included estimated total intracranial volume (eTIV).

Adjusted standardized effect sizes (d_adj) were calculated as the model-adjusted epilepsy-minus-control contrast divided by the residual standard deviation of the corresponding model and are presented with 95% confidence intervals (CIs). The 95% CIs for d_adj were obtained by standardizing the lower and upper confidence limits of the corresponding model-adjusted group contrast by the residual standard deviation from the same model. Positive values indicate higher values in the epilepsy group, whereas negative values indicate lower values in the epilepsy group.

Model assumptions were evaluated for all EPVS outcomes. Heteroscedasticity was assessed using the Breusch–Pagan (BP) test, and residual-versus-fitted and normal Q–Q plots were visually inspected for systematic residual-pattern and distributional departures. Potentially influential observations were evaluated jointly using studentized residuals, leverage, and Cook's distance. Multicollinearity was low across the fitted models (maximum VIF, 1.24). Diagnostic criteria were used to identify observations and models requiring further examination rather than as automatic exclusion rules. No participant was excluded solely on the basis of model diagnostics, and no single observation was judged to warrant exclusion after joint consideration of the influence diagnostics.

HC3 heteroscedasticity-consistent inference was evaluated uniformly across all EPVS models as a robustness analysis. Participant-level nonparametric bootstrap inference with 5,000 resamples and bias-corrected and accelerated (BCa) 95% CIs was additionally used to assess the stability of the adjusted group contrasts. Because the right and left CSO-to-BG ratio models showed the clearest departures from conventional model assumptions, log-transformed CSO-to-BG ratios were additionally evaluated as targeted sensitivity analyses. The log-ratio models yielded adjusted group effects of −0.48 (95% CI, −0.69 to −0.27; P<.001) for the right hemisphere and −0.47 (95% CI, −0.68 to −0.26; P<.001) for the left hemisphere, with directions and inferential conclusions concordant with the corresponding original-scale analyses.

Across the HC3, bootstrap, and targeted transformation analyses, the direction and substantive interpretation of the adjusted group effects were concordant with the corresponding principal models; none altered the substantive pattern of FDR-supported versus unsupported findings established in the principal Table 5 analyses. All quantitative EPVS phenotype comparisons remained within the single EPVS hypothesis family specified for the principal analyses in Table 5. Benjamini–Hochberg false discovery rate (FDR) correction was retained for these principal between-group comparisons. Separate multiplicity families were not defined for HC3, bootstrap, or transformation-based sensitivity analyses because these analyses were undertaken to evaluate robustness of the corresponding principal models rather than as additional independent hypothesis tests.

Abbreviations: BG, basal ganglia; BP, Breusch–Pagan; BCa, bias-corrected and accelerated; CI, confidence interval; CSO, centrum semiovale; d_adj, adjusted standardized between-group effect; EPVS, enlarged perivascular spaces; eTIV, estimated total intracranial volume; FDR, false discovery rate; HC3, heteroscedasticity-consistent covariance estimator type 3; VIF, variance inflation factor.
