# Supplementary material for: Quantitative MRI Characterization of Fluid-Related Phenotypes in Pediatric Epilepsy: Deep Learning-Assisted Assessment of Enlarged Perivascular Space Burden, Regional Distribution, and Clinical Associations
Source: Diagnostics (Basel). 2026 Oct 11;16(20):3304. doi: 10.3390/diagnostics16203304 (PMC13703179; doi:10.3390/diagnostics16203304)
Supplement: Supplementary file 1 [file diagnostics-16-03304-s001.zip › Supplementary Table S5.pdf]

Supplementary Table S5. Clinical Predictor Interrelationships, Collinearity Diagnostics, and Sensitivity Analyses of Clinical Associations With EPVS Measures

Panel A. Pairwise Associations Among Major Clinical Predictors in the Epilepsy Group

Clinical predictor pair	Spearman's ϱ	95% CI	P value
**Temporally related clinical variables**			
Age at MRI – age at epilepsy onset	0.35	0.18 to 0.50	<0.001
Age at MRI – disease duration	0.27	0.10 to 0.43	0.003
Age at epilepsy onset – disease duration	−0.76	−0.82 to −0.67	<0.001
**Treatment-related clinical variables**			
Number of ASMs at MRI – polytherapy	0.86	0.81 to 0.90	<0.001
Number of ASMs at MRI – drug resistance	0.56	0.42 to 0.67	<0.001
Polytherapy – drug resistance	0.49	0.34 to 0.61	<0.001

Panel B. Collinearity Diagnostics for Temporally and Treatment-Related Clinical Predictor Sets

Predictor set	Clinical predictor	Tolerance	VIF
Temporal predictor set	Age at MRI	0.14	7.1
	Age at epilepsy onset	0.045	22.2
	Disease duration	0.041	24.4
Treatment-related predictor set	Number of ASMs at MRI	0.27	3.7
	Polytherapy	0.29	3.4
	Drug resistance	0.64	1.6

Panel C. Selected Sensitivity Analyses for Major Clinical Predictors

Clinical predictor	EPVS measure	Principal adjusted estimate (95% CI)	P value	Sensitivity-model estimate (95% CI)	P value	Additional clinical adjustment
Disease duration	Total EPVS volume	48.7 (25.1 to 72.3)	<0.001	42.1 (17.0 to 67.2)	0.001	Age at MRI omitted
	Right BG-EPVS volume	8.1 (4.8 to 11.4)	<0.001	9.6 (4.1 to 15.1)	0.001	Age at MRI omitted
	Left BG-EPVS volume	7.4 (4.2 to 10.6)	<0.001	8.8 (3.6 to 14.0)	0.001	Age at MRI omitted
	Total BG-EPVS volume	15.5 (10.2 to 20.8)	<0.001	18.4 (8.1 to 28.7)	<0.001	Age at MRI omitted
Drug resistance	Total EPVS volume	237.4 (96.6 to 378.2)	0.001	205 (31 to 379)	0.021	Number of ASMs
	Total BG-EPVS	95.1 (61.5 to 128.7)	<0.001	118 (48 to 188)	0.001	Number of ASMs

	volume					
Drug resistance	Total BG-EPVS volume	95.1 (61.5 to 128.7)	<0.001	126 (53 to 199)	<0.001	Polytherapy
Number of ASMs	Total BG-EPVS volume	25.8 (11.0 to 40.6)	0.001	34 (5 to 63)	0.022	Drug resistance
Polytherapy	Total BG-EPVS volume	64.9 (31.1 to 98.7)	<0.001	69 (−3 to 141)	0.060	Drug resistance

Table note: Panel A summarizes pairwise associations among the major temporally and treatment-related clinical predictors in the epilepsy group. Associations were evaluated using Spearman rank correlation coefficients and are presented with 95% confidence intervals. Binary clinical variables were coded dichotomously for the rank-based analyses. Panel B presents collinearity diagnostics for the temporal predictor set (age at MRI, age at epilepsy onset, and disease duration) and the treatment-related predictor set (number of ASMs at MRI, treatment regimen, and drug resistance). Tolerance and variance inflation factor (VIF) values were used to characterize predictor collinearity. The temporal predictor set was evaluated for diagnostic purposes to quantify the expected mathematical dependence among age at MRI, age at epilepsy onset, and disease duration and was not specified as a simultaneous etiological model. Panel C presents selected sensitivity analyses undertaken to evaluate the stability of the principal clinical associations under alternative adjustment for major related clinical predictors. Sensitivity analyses were focused on total EPVS volume as the principal measure of global EPVS burden and total BG-EPVS volume as the bilateral aggregate measure of the regional phenotype most consistently associated with the major clinical characteristics. For disease duration, right and left BG-EPVS volumes were additionally evaluated to assess the directional consistency of the regional association across hemispheres. Because age at MRI, age at epilepsy onset, and disease duration are mathematically interdependent, disease-duration sensitivity models were repeated without simultaneous adjustment for age at MRI; sex was retained as a covariate, and eTIV was additionally retained for absolute-volume outcomes. Treatment-related sensitivity models retained adjustment for age and sex, with eTIV additionally included for absolute-volume outcomes, and used limited mutual adjustment to evaluate the extent to which associations with drug resistance, ASM number, and treatment regimen were altered after accounting for closely related treatment characteristics. Principal adjusted estimates correspond to the models reported in the main clinical-association analysis, whereas sensitivity-model estimates correspond to the additional models described above. Regression coefficients (B) are reported for continuous predictors and adjusted between-group differences for binary predictors, together with 95% confidence intervals. These sensitivity analyses were exploratory and were undertaken to assess the direction, magnitude, and stability of the corresponding associations rather than to estimate independent causal or clinical effects.

Abbreviations: ASM, antiseizure medication; BG, basal ganglia; CI, confidence interval; EPVS, enlarged perivascular spaces; eTIV, estimated total intracranial volume; MRI, magnetic resonance imaging; VIF, variance inflation factor.
