# Supplementary material for: Quantitative MRI Characterization of Fluid-Related Phenotypes in Pediatric Epilepsy: Deep Learning-Assisted Assessment of Enlarged Perivascular Space Burden, Regional Distribution, and Clinical Associations
Source: Diagnostics (Basel). 2026 Oct 11;16(20):3304. doi: 10.3390/diagnostics16203304 (PMC13703179; doi:10.3390/diagnostics16203304)
Supplement: Supplementary file 1 [file diagnostics-16-03304-s001.zip › Supplementary Table S6.pdf]

Supplementary Table S6. Complete Clinical Predictor–Outcome Matrix and Reproducibility of False Discovery Rate Correction for the Exploratory Clinical-Association Analyses

Panel A. Continuous Clinical Predictors

Clinical predictor	EPVS outcome	Model n	Adjusted B (95% CI)	Raw P value	FDR-adjusted q value
Age at epilepsy onset	Total EPVS volume	123	−29.6 (−63.8 to 4.6)	0.089	0.146
	Right hemispheric EPVS volume	123	−15 (−33 to 3)	0.10	0.16
	Left hemispheric EPVS volume	123	−14 (−31 to 3)	0.11	0.17
	Right BG-EPVS volume	123	−5.1 (−8.9 to −1.3)	0.010	0.034
	Left BG-EPVS volume	123	−4.7 (−8.3 to −1.1)	0.012	0.038
	Total BG-EPVS volume	123	−9.8 (−17.1 to −2.5)	0.009	0.032
	Total BG relative EPVS burden	123	−0.0028 (−0.0051 to −0.0005)	0.018	0.046
	Total CSO relative EPVS burden	123	0.0025 (0.0001 to 0.0049)	0.04	0.07
	Right CSO-to-BG EPVS volume ratio	123	0.07 (−0.01 to 0.15)	0.08	0.13
	Left CSO-to-BG EPVS volume ratio	123	0.07 (−0.01 to 0.15)	0.09	0.14
Disease duration	Total EPVS volume	123	48.7 (25.1 to 72.3)	<0.001	0.002
	Right hemispheric EPVS volume	123	25.9 (12.7 to 39.1)	<0.001	0.002
	Left hemispheric EPVS volume	123	23.8 (10.9 to 36.7)	<0.001	0.003
	Right BG-EPVS volume	123	8.1 (4.8 to 11.4)	<0.001	<0.001
	Left BG-EPVS volume	123	7.4 (4.2 to 10.6)	<0.001	<0.001
	Total BG-EPVS volume	123	15.5 (10.2 to 20.8)	<0.001	<0.001
	Total BG relative EPVS burden	123	0.0040 (0.0023 to 0.0057)	<0.001	<0.001
	Total CSO relative EPVS burden	123	−0.0045 (−0.0068 to −0.0022)	<0.001	0.001
	Right CSO-to-BG EPVS volume ratio	123	−0.101 (−0.151 to −0.051)	<0.001	0.002
	Left CSO-to-BG EPVS volume ratio	123	−0.094 (−0.147 to −0.041)	0.001	0.004
Number of ASMs at MRI	Total EPVS volume	123	81.7 (18.2 to 145.2)	0.012	0.051
	Right hemispheric EPVS volume	123	40 (4 to 76)	0.03	0.06
	Left hemispheric EPVS volume	123	40 (4 to 76)	0.03	0.06
	Right BG-EPVS volume	123	13.6 (5.6 to 21.6)	0.001	0.009
	Left BG-EPVS volume	123	12.2 (4.5 to 19.9)	0.002	0.013
	Total BG-EPVS volume	123	25.8 (11.0 to 40.6)	0.001	0.008

	Total BG relative EPVS burden	123	0.0061 (0.0019 to 0.0103)	0.005	0.024
	Total CSO relative EPVS burden	123	−0.005 (−0.0095 to −0.0005)	0.03	0.06
	Right CSO-to-BG EPVS volume ratio	123	−0.134 (−0.241 to −0.027)	0.015	0.049
	Left CSO-to-BG EPVS volume ratio	123	−0.12 (−0.23 to −0.01)	0.04	0.07

Panel B. Binary Clinical Predictors

**Clinical predictor**	**EPVS outcome**	**Model n**	**Adjusted difference (95% CI)**	**Raw P value**	**FDR-adjusted q value**
Drug-resistant epilepsy	Total EPVS volume	123	237.4 (96.6 to 378.2)	0.001	0.007
	Right hemispheric EPVS volume	123	120 (44 to 196)	0.002	0.013
	Left hemispheric EPVS volume	123	115 (39 to 191)	0.003	0.016
	Right BG-EPVS volume	123	50 (32 to 68)	<0.001	0.002
	Left BG-EPVS volume	123	45 (28 to 62)	<0.001	0.002
	Total BG-EPVS volume	123	95.1 (61.5 to 128.7)	<0.001	<0.001
	Total BG relative EPVS burden	123	0.020 (0.007 to 0.033)	0.002	0.013
	Total CSO relative EPVS burden	123	−0.020 (−0.034 to −0.006)	0.004	0.021
	Right CSO-to-BG EPVS volume ratio	123	−0.45 (−0.76 to −0.14)	0.004	0.021
	Left CSO-to-BG EPVS volume ratio	123	−0.42 (−0.72 to −0.12)	0.006	0.027
Treatment regimen (polytherapy vs monotherapy)	Total EPVS volume	123	175 (8 to 342)	0.04	0.07
	Right hemispheric EPVS volume	123	90 (4 to 176)	0.04	0.07
	Left hemispheric EPVS volume	123	85 (0 to 170)	0.05	0.09
	Right BG-EPVS volume	123	34 (12 to 56)	0.002	0.013
	Left BG-EPVS volume	123	31.1 (13.9 to 48.3)	0.001	0.007
	Total BG-EPVS volume	123	64.9 (31.1 to 98.7)	<0.001	0.004
	Total BG relative EPVS burden	123	0.014 (0.004 to 0.024)	0.006	0.027
	Total CSO relative EPVS burden	123	−0.013 (−0.025 to −0.001)	0.04	0.07
	Right CSO-to-BG EPVS volume	123	−0.39 (−0.73 to −0.05)	0.025	0.048

	ratio				
	Left CSO-to-BG EPVS volume ratio	123	−0.35 (−0.70 to 0.00)	0.051	0.096

Panel C. Multicategorical Clinical and EEG Predictors

Clinical predictor	EPVS outcome	Model n	Category	Adjusted EMM (95% CI)	Omnibus F	Raw P value	FDR-adjusted q value
**Epilepsy type**	**Total EPVS volume**	123	Focal	2118 (2029–2207)	1.32	0.271	0.374
			Generalized	2056 (1957–2155)			
			Combined generalized and focal	2167 (2021–2313)			
			Unknown	2094 (1961–2227)			
	**Total BG-EPVS volume**	123	Focal	452 (427–477)	1.81	0.149	0.252
			Generalized	425 (397–453)			
			Combined generalized and focal	466 (425–507)			
			Unknown	438 (401–475)			
**EEG classification**	**Total EPVS volume**	123	Normal	2054 (1948–2160)	1.76	0.159	0.261
			Focal epileptiform	2146 (2053–2239)			
			Generalized epileptiform	2078 (1972–2184)			
			Multifocal epileptiform	2191 (2048–2334)			
	**Total BG-EPVS volume**	123	Normal	417 (386–448)	2.71	0.048	0.104
			Focal epileptiform	451 (425–477)			
			Generalized epileptiform	431 (401–461)			
			Multifocal epileptiform	472 (432–512)			
**EEG lateralization**	**Total EPVS volume**	80	Right	2102 (1995–2209)	3.24	0.045	0.094
			Left	2118 (2014–2222)			
			Bilateral	2247 (2143–2351)			
	**Right hemispheric EPVS volume**	80	Right	1058 (1000–1116)	4.18	0.019	0.054
			Left	1067 (1010–1124)			

			Bilateral	1145 (1088–1202)			
	**Left hemispheric EPVS volume**	80	Right	1044 (987–1101)	3.71	0.029	0.067
			Left	1051 (995–1107)			
			Bilateral	1102 (1046–1158)			
	**Right BG-EPVS volume**	80	Right	216 (198–234)	5.21	0.007	0.029
			Left	219 (201–237)			
			Bilateral	248 (229–267)			
	**Left BG-EPVS volume**	80	Right	213 (195–231)	4.66	0.012	0.041
			Left	215 (198–232)			
			Bilateral	242 (224–260)			
	**Total BG-EPVS volume**	80	Right	429 (401–457)	5.74	0.005	0.024
			Left	434 (406–462)			
			Bilateral	490 (462–518)			
	**Total BG relative EPVS burden**	80	Right	0.205 (0.193–0.217)	4.91	0.010	0.037
			Left	0.207 (0.195–0.219)			
			Bilateral	0.229 (0.216–0.242)			
	**Total CSO relative EPVS burden**	80	Right	0.795 (0.783–0.807)	4.91	0.010	0.037
			Left	0.793 (0.781–0.805)			
			Bilateral	0.771 (0.758–0.784)			
**EEG anatomical localization**	**Total EPVS volume**	80	Temporal	2143 (2027–2259)	1.37	0.251	0.361
			Frontal	2167 (2041–2293)			
			Centroparietal/ posterior	2098 (1962–2234)			
			Multifocal	2214 (2072–2356)			
			Indeterminate	2112 (1973–2251)			
	**Total BG-EPVS volume**	80	Temporal	447 (414–480)	2.02	0.099	0.187
			Frontal	455 (420–490)			
			Centroparietal/ posterior	432 (394–470)			
			Multifocal	474 (434–514)			
			Indeterminate	441 (403–479)			

Table note. This table provides the complete inferential test matrix used for the exploratory clinical-association analyses and documents the multiplicity structure underlying the reported false discovery

rate (FDR)-adjusted results. Clinical-association analyses were performed within the epilepsy group and were restricted to EPVS measures that had shown significant epilepsy–control differences after FDR correction in the preceding between-group analysis. Continuous clinical predictors and binary disease- and treatment-related characteristics were evaluated across the full set of EPVS measures entering the clinical-association stage. For the multicategorical variables of epilepsy type, EEG classification, and EEG anatomical localization, analyses focused on the principal global burden measure (total EPVS volume) and the principal regional burden measure (total BG-EPVS volume). EEG lateralization was evaluated across a broader set of global, hemispheric, BG, and relative regional-distribution measures because this variable specifically encodes the laterality of electrophysiological abnormalities. For continuous clinical predictors, effect estimates are presented as unstandardized regression coefficients (B) with 95% confidence intervals (CIs); for binary clinical predictors, effects are presented as model-derived adjusted between-group differences with 95% CIs; and for multicategorical clinical and EEG predictors, the corresponding omnibus F statistics are reported. All models were adjusted for age and sex, with estimated total intracranial volume (eTIV) additionally included for absolute EPVS-volume outcomes. eTIV was not additionally included for relative EPVS-burden measures or CSO-to-BG ratios. No missing data were imputed, and model-specific sample sizes reflect participants with complete data for all variables required for the corresponding analysis. EEG lateralization and anatomical-localization analyses were restricted to participants for whom the respective characteristic could be defined and category sizes were sufficient for formal analysis; participants outside these eligible subgroups were not classified as having missing EEG lateralization or localization data solely because the corresponding analysis was not applicable. The two-sided raw P values for the clinical-variable coefficients in continuous-predictor models, group effects in binary-predictor models, and omnibus effects in multicategorical-predictor models constituted a single clinical-association FDR family comprising 64 inferential tests and were adjusted jointly using the Benjamini–Hochberg procedure. Category-specific estimated marginal means did not constitute additional tests in this FDR family. Post hoc pairwise estimated-marginal-mean contrasts were performed only for omnibus effects that remained significant after FDR correction and were adjusted separately using the Tukey procedure; these contrasts were therefore not included in the 64-test Benjamini–Hochberg family. Because entry of EPVS outcomes into the clinical-association stage depended on statistical significance in the preceding epilepsy–control comparison, these analyses are post-selection and exploratory. Accordingly, the reported q values account for multiplicity within the second-stage clinical-association family but do not account for uncertainty introduced by the preceding data-dependent outcome-selection step.

Abbreviations: ASM, antiseizure medication; BG, basal ganglia; CI, confidence interval; CSO, centrum semiovale; EEG, electroencephalography; EPVS, enlarged perivascular spaces; FDR, false discovery rate; MRI, magnetic resonance imaging; eTIV, estimated total intracranial volume.
