# Supplementary material for: Quantitative MRI Characterization of Fluid-Related Phenotypes in Pediatric Epilepsy: Deep Learning-Assisted Assessment of Enlarged Perivascular Space Burden, Regional Distribution, and Clinical Associations
Source: Diagnostics (Basel). 2026 Oct 11;16(20):3304. doi: 10.3390/diagnostics16203304 (PMC13703179; doi:10.3390/diagnostics16203304)
Supplement: Supplementary file 1 [file diagnostics-16-03304-s001.zip › Supplementary Table S7.pdf]

Supplementary Table S7. Selection-Independent Sensitivity Analysis of Clinical Associations Across an Expanded EPVS Outcome Set

Panel A. Continuous clinical predictors

Clinical predictor	EPVS outcome	Model n	Adjusted B (95% CI)	Raw P value	Expanded-family FDR q value
Age at epilepsy onset	Total EPVS volume	123	−29.6 (−63.8 to 4.6)	0.089	0.145
	Right hemispheric EPVS volume	123	−15 (−33 to 3)	0.100	0.156
	Left hemispheric EPVS volume	123	−14 (−31 to 3)	0.110	0.169
	Right CSO-EPVS volume	123	−3.9 (−11.5 to 3.7)	0.420	0.465
	Left CSO-EPVS volume	123	−4.1 (−11.6 to 3.4)	0.380	0.433
	Total CSO-EPVS volume	123	−8.0 (−22.8 to 6.8)	0.400	0.451
	Right BG-EPVS volume	123	−5.1 (−8.9 to −1.3)	0.010	0.028
	Left BG-EPVS volume	123	−4.7 (−8.3 to −1.1)	0.012	0.030
	Total BG-EPVS volume	123	−9.8 (−17.1 to −2.5)	0.009	0.027
	Hemispheric EPVS asymmetry index	123	−0.003 (−0.015 to 0.009)	0.610	0.644
	CSO-EPVS asymmetry index	123	0.002 (−0.010 to 0.014)	0.740	0.747
	BG-EPVS asymmetry index	123	−0.007 (−0.020 to 0.006)	0.290	0.371
	Right BG relative EPVS burden	123	−0.0027 (−0.0053 to −0.0001)	0.020	0.045
	Left BG relative EPVS burden	123	−0.0029 (−0.0056 to −0.0002)	0.022	0.048
	Total BG relative EPVS burden	123	−0.0028 (−0.0051 to −0.0005)	0.018	0.042
	Total CSO relative EPVS burden	123	0.0025 (0.0001 to 0.0049)	0.040	0.074
	Right CSO-to-BG EPVS volume ratio	123	0.07 (−0.01 to 0.15)	0.080	0.134
	Left CSO-to-BG EPVS volume ratio	123	0.07 (−0.01 to 0.15)	0.090	0.145
Disease duration	Total EPVS volume	123	48.7 (25.1 to 72.3)	<0.001	0.004
	Right hemispheric EPVS volume	123	25.9 (12.7 to 39.1)	<0.001	0.004
	Left hemispheric EPVS volume	123	23.8 (10.9 to 36.7)	<0.001	0.004
	Right CSO-EPVS volume	123	3.1 (−2.9 to 9.1)	0.310	0.376
	Left CSO-EPVS volume	123	3.3 (−2.7 to 9.3)	0.280	0.367
	Total CSO-EPVS volume	123	6.4 (−5.7 to 18.5)	0.300	0.376

	Right BG-EPVS volume	123	8.1 (4.8 to 11.4)	<0.001	0.004
	Left BG-EPVS volume	123	7.4 (4.2 to 10.6)	<0.001	0.004
	Total BG-EPVS volume	123	15.5 (10.2 to 20.8)	<0.001	0.004
	Hemispheric EPVS asymmetry index	123	0.003 (−0.005 to 0.011)	0.460	0.504
	CSO-EPVS asymmetry index	123	−0.002 (−0.010 to 0.006)	0.580	0.618
	BG-EPVS asymmetry index	123	0.006 (−0.003 to 0.015)	0.190	0.274
	Right BG relative EPVS burden	123	0.0040 (0.0021 to 0.0059)	<0.001	0.004
	Left BG relative EPVS burden	123	0.0041 (0.0021 to 0.0061)	<0.001	0.005
	Total BG relative EPVS burden	123	0.0040 (0.0023 to 0.0057)	<0.001	0.004
	Total CSO relative EPVS burden	123	−0.0045 (−0.0068 to −0.0022)	<0.001	0.005
	Right CSO-to-BG EPVS volume ratio	123	−0.101 (−0.151 to −0.051)	<0.001	0.004
	Left CSO-to-BG EPVS volume ratio	123	−0.094 (−0.147 to −0.041)	0.001	0.006
Number of ASMs at MRI	Total EPVS volume	123	81.7 (18.2 to 145.2)	0.012	0.030
	Right hemispheric EPVS volume	123	40 (4 to 76)	0.030	0.060
	Left hemispheric EPVS volume	123	40 (4 to 76)	0.030	0.060
	Right CSO-EPVS volume	123	8.2 (−8.7 to 25.1)	0.340	0.400
	Left CSO-EPVS volume	123	8.8 (−8.3 to 25.9)	0.310	0.376
	Total CSO-EPVS volume	123	17.0 (−16.8 to 50.8)	0.320	0.384
	Right BG-EPVS volume	123	13.6 (5.6 to 21.6)	0.001	0.006
	Left BG-EPVS volume	123	12.2 (4.5 to 19.9)	0.002	0.010
	Total BG-EPVS volume	123	25.8 (11.0 to 40.6)	0.001	0.006
	Hemispheric EPVS asymmetry index	123	0.005 (−0.018 to 0.028)	0.670	0.694
	CSO-EPVS asymmetry index	123	−0.004 (−0.027 to 0.019)	0.730	0.743
	BG-EPVS asymmetry index	123	0.015 (−0.009 to 0.039)	0.220	0.314
	Right BG relative EPVS burden	123	0.0062 (0.0018 to 0.0106)	0.007	0.023
	Left BG relative EPVS burden	123	0.0060 (0.0015 to 0.0105)	0.009	0.027
	Total BG relative EPVS burden	123	0.0061 (0.0019 to 0.0103)	0.005	0.018
	Total CSO relative EPVS burden	123	−0.005 (−0.0095 to −0.0005)	0.030	0.060
	Right CSO-to-BG	123	−0.134 (−0.241 to	0.015	0.036

	EPVS volume ratio		−0.027)		
	Left CSO-to-BG EPVS volume ratio	123	−0.12 (−0.23 to −0.01)	0.040	0.074

Panel B. Binary Clinical Predictors

Clinical predictor	EPVS outcome	Model n	Adjusted difference (95% CI)	Raw P value	Expanded-family FDR q value
Drug-resistant epilepsy	Total EPVS volume	123	237.4 (96.6 to 378.2)	0.001	0.006
	Right hemispheric EPVS volume	123	120 (44 to 196)	0.002	0.010
	Left hemispheric EPVS volume	123	115 (39 to 191)	0.003	0.013
	Right CSO-EPVS volume	123	27 (−23 to 77)	0.290	0.371
	Left CSO-EPVS volume	123	29 (−21 to 79)	0.250	0.349
	Total CSO-EPVS volume	123	56 (−44 to 156)	0.270	0.363
	Right BG-EPVS volume	123	50 (32 to 68)	<0.001	0.004
	Left BG-EPVS volume	123	45 (28 to 62)	<0.001	0.004
	Total BG-EPVS volume	123	95.1 (61.5 to 128.7)	<0.001	0.004
	Hemispheric EPVS asymmetry index	123	0.019 (−0.042 to 0.080)	0.540	0.581
	CSO-EPVS asymmetry index	123	−0.015 (−0.075 to 0.045)	0.620	0.648
	BG-EPVS asymmetry index	123	0.043 (−0.018 to 0.104)	0.170	0.248
	Right BG relative EPVS burden	123	0.021 (0.007 to 0.035)	0.003	0.013
	Left BG relative EPVS burden	123	0.019 (0.006 to 0.032)	0.004	0.016
	Total BG relative EPVS burden	123	0.020 (0.007 to 0.033)	0.002	0.010
	Total CSO relative EPVS burden	123	−0.020 (−0.034 to −0.006)	0.004	0.016
	Right CSO-to-BG EPVS volume ratio	123	−0.45 (−0.76 to −0.14)	0.004	0.016
	Left CSO-to-BG EPVS volume ratio	123	−0.42 (−0.72 to −0.12)	0.006	0.021
Polytherapy vs monotherapy	Total EPVS volume	123	175 (8 to 342)	0.040	0.074
	Right hemispheric EPVS volume	123	90 (4 to 176)	0.040	0.074
	Left hemispheric EPVS volume	123	85 (0 to 170)	0.050	0.088
	Right CSO-EPVS volume	123	25 (−29 to 79)	0.360	0.415
	Left CSO-EPVS volume	123	27 (−26 to 80)	0.330	0.392

	Total CSO-EPVS volume	123	52 (−57 to 161)	0.350	0.407
	Right BG-EPVS volume	123	34 (12 to 56)	0.002	0.010
	Left BG-EPVS volume	123	31.1 (13.9 to 48.3)	0.001	0.006
	Total BG-EPVS volume	123	64.9 (31.1 to 98.7)	<0.001	0.004
	Hemispheric EPVS asymmetry index	123	0.013 (−0.056 to 0.082)	0.710	0.729
	CSO-EPVS asymmetry index	123	−0.009 (−0.077 to 0.059)	0.790	0.790
	BG-EPVS asymmetry index	123	0.039 (−0.029 to 0.107)	0.260	0.357
	Right BG relative EPVS burden	123	0.015 (0.004 to 0.026)	0.009	0.027
	Left BG relative EPVS burden	123	0.014 (0.003 to 0.025)	0.011	0.030
	Total BG relative EPVS burden	123	0.014 (0.004 to 0.024)	0.006	0.021
	Total CSO relative EPVS burden	123	−0.013 (−0.025 to −0.001)	0.040	0.074
	Right CSO-to-BG EPVS volume ratio	123	−0.39 (−0.73 to −0.05)	0.025	0.054
	Left CSO-to-BG EPVS volume ratio	123	−0.35 (−0.70 to 0.00)	0.051	0.088

Panel C. Multicategorical Clinical and EEG Predictors

Clinical predictor	EPVS outcome	Model n	Category	Adjusted EMM (95% CI)	Omnibus F	Raw P	Expanded q
Epilepsy type	Total EPVS volume	123	Focal	2118 (2029–2207)	1.32	0.271	0.363
			Generalized	2056 (1957–2155)			
			Combined generalized and focal	2167 (2021–2313)			
			Unknown	2094 (1961–2227)			
Epilepsy type	Total BG-EPVS volume	123	Focal	452 (427–477)	1.81	0.149	0.226
			Generalized	425 (397–453)			
			Combined generalized and focal	466 (425–507)			
			Unknown	438 (401–475)			

Clinical predictor	EPVS outcome	Model n	Category	Adjusted EMM (95% CI)	Omnibus F	Raw P	Expanded q
EEG classification	Total EPVS volume	123	Normal	2054 (1948–2160)	1.76	0.159	0.237
			Focal epileptiform	2146 (2053–2239)			
			Generalized epileptiform	2078 (1972–2184)			
			Multifocal epileptiform	2191 (2048–2334)			
EEG classification	Total BG-EPVS volume	123	Normal	417 (386–448)	2.71	0.048	0.086
			Focal epileptiform	451 (425–477)			
			Generalized epileptiform	431 (401–461)			
			Multifocal epileptiform	472 (432–512)			

EPVS outcome	Model n	Category	Adjusted EMM (95% CI)	Omnibus F	Raw P	Expanded q
Total EPVS volume	80	Right	2102 (1995–2209)	3.24	0.045	0.081
		Left	2118 (2014–2222)			
		Bilateral	2247 (2143–2351)			
Right hemispheric EPVS volume	80	Right	1058 (1000–1116)	4.18	0.019	0.043
		Left	1067 (1010–1124)			
		Bilateral	1145 (1088–1202)			
Left hemispheric EPVS volume	80	Right	1044 (987–1101)	3.71	0.029	0.060
		Left	1051 (995–1107)			
		Bilateral	1102 (1046–1158)			
Right CSO-EPVS volume	80	Right	398 (369–427)	1.18	0.310	0.376
		Left	405 (376–434)			
		Bilateral	416 (386–446)			
Left CSO-EPVS volume	80	Right	401 (372–430)	1.29	0.280	0.367
		Left	407 (378–436)			
		Bilateral	420 (390–450)			
Total CSO-EPVS volume	80	Right	799 (744–854)	1.22	0.300	0.376
		Left	812 (757–867)			
		Bilateral	836 (779–893)			
Right BG-EPVS volume	80	Right	216 (198–234)	5.21	0.007	0.023
		Left	219 (201–237)			
		Bilateral	248 (229–267)			
Left BG-EPVS volume	80	Right	213 (195–231)	4.66	0.012	0.030
		Left	215 (198–232)			

		Bilateral	242 (224–260)			
Total BG-EPVS volume	80	Right	429 (401–457)	5.74	0.005	0.018
		Left	434 (406–462)			
		Bilateral	490 (462–518)			
Hemispheric EPVS asymmetry index	80	Right	0.012 (−0.017 to 0.041)	0.90	0.410	0.458
		Left	−0.009 (−0.038 to 0.020)			
		Bilateral	0.003 (−0.026 to 0.032)			
CSO-EPVS asymmetry index	80	Right	0.016 (−0.016 to 0.048)	0.66	0.520	0.565
		Left	−0.012 (−0.044 to 0.020)			
		Bilateral	0.004 (−0.028 to 0.036)			
BG-EPVS asymmetry index	80	Right	0.014 (−0.018 to 0.046)	1.87	0.160	0.237
		Left	−0.011 (−0.043 to 0.021)			
		Bilateral	0.002 (−0.030 to 0.034)			
Right BG relative EPVS burden	80	Right	0.207 (0.194–0.220)	4.45	0.014	0.035
		Left	0.204 (0.192–0.216)			
		Bilateral	0.229 (0.216–0.242)			
Left BG relative EPVS burden	80	Right	0.203 (0.191–0.215)	4.30	0.016	0.038
		Left	0.210 (0.197–0.223)			
		Bilateral	0.229 (0.216–0.242)			
Total BG relative EPVS burden	80	Right	0.205 (0.193–0.217)	4.91	0.010	0.028
		Left	0.207 (0.195–0.219)			
		Bilateral	0.229 (0.216–0.242)			
Total CSO relative EPVS burden	80	Right	0.795 (0.783–0.807)	4.91	0.010	0.028
		Left	0.793 (0.781–0.805)			
		Bilateral	0.771 (0.758–0.784)			
Right CSO-to-BG EPVS volume ratio	80	Right	3.78 (3.42–4.14)	2.61	0.080	0.134
		Left	3.83 (3.47–4.19)			
		Bilateral	3.41 (3.05–3.77)			
Left CSO-to-BG EPVS volume ratio	80	Right	3.86 (3.49–4.23)	2.49	0.090	0.145
		Left	3.79 (3.43–4.15)			
		Bilateral	3.45 (3.08–3.82)			

Clinical predictor	EPVS outcome	Model n	Category	Adjusted EMM (95% CI)	Omnibus F	Raw P	Expanded q
EEG anatomical localization	Total EPVS volume	80	Temporal	2143 (2027–2259)	1.37	0.251	0.349
			Frontal	2167 (2041–2293)			
			Centroparietal/posterior	2098 (1962–2234)			
			Multifocal	2214 (2072–2356)			
			Indeterminate	2112 (1973–2251)			
EEG anatomical localization	Total BG-EPVS volume	80	Temporal	447 (414–480)	2.02	0.099	0.156
			Frontal	455 (420–490)			
			Centroparietal/posterior	432 (394–470)			
			Multifocal	474 (434–514)			
			Indeterminate	441 (403–479)			

Table Note: This sensitivity analysis was performed to evaluate the robustness of the exploratory clinical-association findings to the data-dependent EPVS outcome-selection step used in the primary clinical analyses. In contrast to the primary clinical-association analysis, in which clinical models were restricted to EPVS measures showing FDR-significant epilepsy–control differences in the preceding between-group analysis, the present sensitivity analysis removed this significance-based selection criterion and evaluated the corresponding clinical associations across an expanded 18-measure EPVS outcome set, while otherwise retaining the predictor-specific analytical architecture used in the primary clinical analyses. Continuous clinical predictors and binary disease- and treatment-related characteristics were evaluated across all 18 EPVS measures in this expanded outcome set. Analyses of epilepsy type, EEG classification, and EEG anatomical localization retained their focus on the principal global burden measure (total EPVS volume) and the principal regional burden measure (total BG-EPVS volume), whereas EEG lateralization was evaluated across the same expanded 18-measure EPVS outcome set because this variable specifically encodes the hemispheric distribution of electrophysiological abnormalities. Continuous-predictor effects are presented as unstandardized regression coefficients (B) with 95% confidence intervals (CIs), binary-predictor effects as model-derived adjusted between-group differences with 95% CIs, and multicategorical clinical and EEG predictors as category-specific adjusted estimated marginal means (EMMs) with 95% CIs together with the corresponding omnibus F statistics. All models were adjusted for age and sex, with estimated total intracranial volume

(eTIV) additionally included for absolute EPVS-volume outcomes. eTIV was not additionally included for asymmetry indices, relative EPVS-burden measures, or CSO-to-BG ratios. No missing data were imputed, and model-specific sample sizes reflect participants with complete data for all variables required for the corresponding analysis. EEG lateralization and anatomical-localization analyses were restricted to participants for whom the respective characteristic could be defined and category sizes were sufficient for formal analysis; participants outside these eligible subgroups were not classified as having missing data solely because the corresponding analysis was not applicable. The two-sided raw P values for the clinical-variable coefficients in continuous-predictor models, group effects in binary-predictor models, and omnibus effects in multicategorical-predictor models constituted a single expanded sensitivity-analysis FDR family comprising 114 inferential tests and were adjusted jointly using the Benjamini–Hochberg procedure. Category-specific EMMs did not constitute additional tests in this FDR family. Post hoc pairwise EMM contrasts, where applicable, were not included in the 114-test Benjamini–Hochberg family. Because the expanded sensitivity-analysis family differs from the 64-test selected-outcome clinical-association family reported in Supplementary Table S6, the corresponding FDR-adjusted q values are not expected to be identical. This analysis was conducted as an exploratory sensitivity analysis to characterize the influence of the preceding outcome-selection step and was not used to redefine the primary clinical-association analysis.

Abbreviations: ASM, antiseizure medication; B, unstandardized regression coefficient; BG, basal ganglia; CI, confidence interval; CSO, centrum semiovale; EEG, electroencephalography; EMM, estimated marginal mean; EPVS, enlarged perivascular spaces; eTIV, estimated total intracranial volume; FDR, false discovery rate; MRI, magnetic resonance imaging.
