# Supplementary material for: Quantitative MRI Characterization of Fluid-Related Phenotypes in Pediatric Epilepsy: Deep Learning-Assisted Assessment of Enlarged Perivascular Space Burden, Regional Distribution, and Clinical Associations
Source: Diagnostics (Basel). 2026 Oct 11;16(20):3304. doi: 10.3390/diagnostics16203304 (PMC13703179; doi:10.3390/diagnostics16203304)
Supplement: Supplementary file 1 [file diagnostics-16-03304-s001.zip › Supplementary Table S8.pdf]

Supplementary Table S8. Full-Precision Raw P-Value Vectors for Reproduction of the Benjamini–Hochberg False Discovery Rate Adjustments

Panel A. Specification of the FDR Families

Analysis family	Outcome-selection rule	Inferential P values included	Number of tests	Excluded from FDR family	Multiplicity adjustment
Primary exploratory clinical-association analysis	Clinical analyses were restricted to EPVS measures showing FDR-significant epilepsy–control differences in the preceding between-group analysis; the predictor-specific analytical architecture reported in Supplementary Table S6 was then applied.	Two-sided raw P values for the clinical-variable coefficients in continuous-predictor models, group effects in binary-predictor models, and omnibus effects in multicategorical-predictor models.	64	Category-specific EMMs and post hoc pairwise EMM contrasts. Post hoc contrasts, where applicable, were adjusted separately using the Tukey procedure.	Benjamini–Hochberg
Expanded post-selection sensitivity analysis	The preceding significance-based EPVS outcome-selection criterion was removed, and the corresponding clinical associations were evaluated across an expanded 18-measure EPVS outcome set while otherwise retaining the predictor-specific analytical architecture used in the primary clinical analyses.	Two-sided raw P values for the clinical-variable coefficients in continuous-predictor models, group effects in binary-predictor models, and omnibus effects in multicategorical-predictor models.	114	Category-specific EMMs and post hoc pairwise EMM contrasts.	Benjamini–Hochberg

Panel B. Primary Exploratory Clinical-Association Family

Test ID	Clinical predictor	EPVS outcome	Raw P value, full computational precision
P01	Age at epilepsy onset	Total EPVS volume	0.0891746
P02	Age at epilepsy onset	Right hemispheric EPVS volume	0.0996842
P03	Age at epilepsy onset	Left hemispheric EPVS volume	0.1096387
P04	Age at epilepsy onset	Right BG-EPVS volume	0.0096841
P05	Age at epilepsy onset	Left BG-EPVS volume	0.0117426

P06	Age at epilepsy onset	Total BG-EPVS volume	0.0087315
P07	Age at epilepsy onset	Total BG relative EPVS burden	0.0176934
P08	Age at epilepsy onset	Total CSO relative EPVS burden	0.0396172
P09	Age at epilepsy onset	Right CSO-to-BG EPVS volume ratio	0.080538
P10	Age at epilepsy onset	Left CSO-to-BG EPVS volume ratio	0.090584
P11	Disease duration	Total EPVS volume	0.00007997
P12	Disease duration	Right hemispheric EPVS volume	0.0001686
P13	Disease duration	Left hemispheric EPVS volume	0.0003864
P14	Disease duration	Right BG-EPVS volume	0.00000362
P15	Disease duration	Left BG-EPVS volume	0.00001158
P16	Disease duration	Total BG-EPVS volume	0.000000058
P17	Disease duration	Total BG relative EPVS burden	0.00000835
P18	Disease duration	Total CSO relative EPVS burden	0.0001755
P19	Disease duration	Right CSO-to-BG EPVS volume ratio	0.000167
P20	Disease duration	Left CSO-to-BG EPVS volume ratio	0.001987
P21	Number of ASMs at MRI	Total EPVS volume	0.0118214
P22	Number of ASMs at MRI	Right hemispheric EPVS volume	0.0296417
P23	Number of ASMs at MRI	Left hemispheric EPVS volume	0.0302146
P24	Number of ASMs at MRI	Right BG-EPVS volume	0.0009187
P25	Number of ASMs at MRI	Left BG-EPVS volume	0.0018743
P26	Number of ASMs at MRI	Total BG-EPVS volume	0.0007862
P27	Number of ASMs at MRI	Total BG relative EPVS burden	0.0048127
P28	Number of ASMs at MRI	Total CSO relative EPVS burden	0.0297843
P29	Number of ASMs at MRI	Right CSO-to-BG EPVS volume ratio	0.015123
P30	Number of ASMs at MRI	Left CSO-to-BG EPVS volume ratio	0.040628
P31	Drug-resistant epilepsy	Total EPVS volume	0.0009462
P32	Drug-resistant epilepsy	Right hemispheric EPVS volume	0.0018427
P33	Drug-resistant epilepsy	Left hemispheric EPVS volume	0.0027314
P34	Drug-resistant epilepsy	Right BG-EPVS volume	0.00000011
P35	Drug-resistant epilepsy	Left BG-EPVS volume	0.00000031
P36	Drug-resistant epilepsy	Total BG-EPVS volume	0.000000006
P37	Drug-resistant epilepsy	Total BG relative EPVS burden	0.0018635
P38	Drug-resistant epilepsy	Total CSO relative EPVS burden	0.0039421

P39	Drug-resistant epilepsy	Right CSO-to-BG EPVS volume ratio	0.004612
P40	Drug-resistant epilepsy	Left CSO-to-BG EPVS volume ratio	0.006285
P41	Polytherapy vs monotherapy	Total EPVS volume	0.0398627
P42	Polytherapy vs monotherapy	Right hemispheric EPVS volume	0.0401735
P43	Polytherapy vs monotherapy	Left hemispheric EPVS volume	0.0496814
P44	Polytherapy vs monotherapy	Right BG-EPVS volume	0.0022148
P45	Polytherapy vs monotherapy	Left BG-EPVS volume	0.0007416
P46	Polytherapy vs monotherapy	Total BG-EPVS volume	0.0001843
P47	Polytherapy vs monotherapy	Total BG relative EPVS burden	0.0057318
P48	Polytherapy vs monotherapy	Total CSO relative EPVS burden	0.0395826
P49	Polytherapy vs monotherapy	Right CSO-to-BG EPVS volume ratio	0.025238
P50	Polytherapy vs monotherapy	Left CSO-to-BG EPVS volume ratio	0.051639
P51	Epilepsy type	Total EPVS volume	0.271218
P52	Epilepsy type	Total BG-EPVS volume	0.148912
P53	EEG classification	Total EPVS volume	0.158764
P54	EEG classification	Total BG-EPVS volume	0.048327
P55	EEG lateralization	Total EPVS volume	0.044783
P56	EEG lateralization	Right hemispheric EPVS volume	0.018947
P57	EEG lateralization	Left hemispheric EPVS volume	0.028816
P58	EEG lateralization	Right BG-EPVS volume	0.007386
P59	EEG lateralization	Left BG-EPVS volume	0.012437
P60	EEG lateralization	Total BG-EPVS volume	0.004817
P61	EEG lateralization	Total BG relative EPVS burden	0.009846
P62	EEG lateralization	Total CSO relative EPVS burden	0.009846
P63	EEG anatomical localization	Total EPVS volume	0.250684
P64	EEG anatomical localization	Total BG-EPVS volume	0.099217

Panel C. Expanded Post-Selection Sensitivity-Analysis Family

Test ID	Clinical predictor	EPVS outcome	Raw P value, full computational precision
S001	Age at epilepsy onset	Total EPVS volume	0.0891746
S002	Age at epilepsy onset	Right hemispheric EPVS volume	0.0996842
S003	Age at epilepsy onset	Left hemispheric EPVS volume	0.1096387
S004	Age at epilepsy onset	Right CSO-EPVS volume	0.4196721
S005	Age at epilepsy onset	Left CSO-EPVS volume	0.3798463

S006	Age at epilepsy onset	Total CSO-EPVS volume	0.3997318
S007	Age at epilepsy onset	Right BG-EPVS volume	0.0096841
S008	Age at epilepsy onset	Left BG-EPVS volume	0.0117426
S009	Age at epilepsy onset	Total BG-EPVS volume	0.0087315
S010	Age at epilepsy onset	Hemispheric EPVS asymmetry index	0.6097284
S011	Age at epilepsy onset	CSO-EPVS asymmetry index	0.7396142
S012	Age at epilepsy onset	BG-EPVS asymmetry index	0.2897816
S013	Age at epilepsy onset	Right BG relative EPVS burden	0.0196847
S014	Age at epilepsy onset	Left BG relative EPVS burden	0.0217463
S015	Age at epilepsy onset	Total BG relative EPVS burden	0.0176934
S016	Age at epilepsy onset	Total CSO relative EPVS burden	0.0396172
S017	Age at epilepsy onset	Right CSO-to-BG EPVS volume ratio	0.0805380
S018	Age at epilepsy onset	Left CSO-to-BG EPVS volume ratio	0.0905840
S019	Disease duration	Total EPVS volume	0.00007997
S020	Disease duration	Right hemispheric EPVS volume	0.0001686
S021	Disease duration	Left hemispheric EPVS volume	0.0003864
S022	Disease duration	Right CSO-EPVS volume	0.3097421
S023	Disease duration	Left CSO-EPVS volume	0.2798164
S024	Disease duration	Total CSO-EPVS volume	0.2996847
S025	Disease duration	Right BG-EPVS volume	0.00000362
S026	Disease duration	Left BG-EPVS volume	0.00001158
S027	Disease duration	Total BG-EPVS volume	0.000000058
S028	Disease duration	Hemispheric EPVS asymmetry index	0.4597281
S029	Disease duration	CSO-EPVS asymmetry index	0.5796134
S030	Disease duration	BG-EPVS asymmetry index	0.1897426
S031	Disease duration	Right BG relative EPVS burden	0.00000835
S032	Disease duration	Left BG relative EPVS burden	0.00001142
S033	Disease duration	Total BG relative EPVS burden	0.00000835
S034	Disease duration	Total CSO relative EPVS burden	0.0001755
S035	Disease duration	Right CSO-to-BG EPVS volume ratio	0.0001670
S036	Disease duration	Left CSO-to-BG EPVS volume ratio	0.0019870
S037	Number of ASMs at MRI	Total EPVS volume	0.0118214
S038	Number of ASMs at MRI	Right hemispheric EPVS volume	0.0296417
S039	Number of ASMs at MRI	Left hemispheric EPVS volume	0.0302146
S040	Number of ASMs at MRI	Right CSO-EPVS volume	0.3396842
S041	Number of ASMs at MRI	Left CSO-EPVS volume	0.3097316

S042	Number of ASMs at MRI	Total CSO-EPVS volume	0.3196475
S043	Number of ASMs at MRI	Right BG-EPVS volume	0.0009187
S044	Number of ASMs at MRI	Left BG-EPVS volume	0.0018743
S045	Number of ASMs at MRI	Total BG-EPVS volume	0.0007862
S046	Number of ASMs at MRI	Hemispheric EPVS asymmetry index	0.6697143
S047	Number of ASMs at MRI	CSO-EPVS asymmetry index	0.7296385
S048	Number of ASMs at MRI	BG-EPVS asymmetry index	0.2197834
S049	Number of ASMs at MRI	Right BG relative EPVS burden	0.0068124
S050	Number of ASMs at MRI	Left BG relative EPVS burden	0.0087641
S051	Number of ASMs at MRI	Total BG relative EPVS burden	0.0048127
S052	Number of ASMs at MRI	Total CSO relative EPVS burden	0.0297843
S053	Number of ASMs at MRI	Right CSO-to-BG EPVS volume ratio	0.0151230
S054	Number of ASMs at MRI	Left CSO-to-BG EPVS volume ratio	0.0406280
S055	Drug-resistant epilepsy	Total EPVS volume	0.0009462
S056	Drug-resistant epilepsy	Right hemispheric EPVS volume	0.0018427
S057	Drug-resistant epilepsy	Left hemispheric EPVS volume	0.0027314
S058	Drug-resistant epilepsy	Right CSO-EPVS volume	0.2896417
S059	Drug-resistant epilepsy	Left CSO-EPVS volume	0.2497836
S060	Drug-resistant epilepsy	Total CSO-EPVS volume	0.2697184
S061	Drug-resistant epilepsy	Right BG-EPVS volume	0.00000011
S062	Drug-resistant epilepsy	Left BG-EPVS volume	0.00000031
S063	Drug-resistant epilepsy	Total BG-EPVS volume	0.000000006
S064	Drug-resistant epilepsy	Hemispheric EPVS asymmetry index	0.5396812
S065	Drug-resistant epilepsy	CSO-EPVS asymmetry index	0.6197425
S066	Drug-resistant epilepsy	BG-EPVS asymmetry index	0.1697843
S067	Drug-resistant epilepsy	Right BG relative EPVS burden	0.0027318
S068	Drug-resistant epilepsy	Left BG relative EPVS burden	0.0039421
S069	Drug-resistant epilepsy	Total BG relative EPVS burden	0.0018635
S070	Drug-resistant epilepsy	Total CSO relative EPVS burden	0.0039421
S071	Drug-resistant epilepsy	Right CSO-to-BG EPVS volume ratio	0.0046120
S072	Drug-resistant epilepsy	Left CSO-to-BG EPVS volume ratio	0.0062850
S073	Polytherapy vs monotherapy	Total EPVS volume	0.0398627
S074	Polytherapy vs monotherapy	Right hemispheric EPVS volume	0.0401735

S075	Polytherapy vs monotherapy	Left hemispheric EPVS volume	0.0496814
S076	Polytherapy vs monotherapy	Right CSO-EPVS volume	0.3597284
S077	Polytherapy vs monotherapy	Left CSO-EPVS volume	0.3296817
S078	Polytherapy vs monotherapy	Total CSO-EPVS volume	0.3497421
S079	Polytherapy vs monotherapy	Right BG-EPVS volume	0.0022148
S080	Polytherapy vs monotherapy	Left BG-EPVS volume	0.0007416
S081	Polytherapy vs monotherapy	Total BG-EPVS volume	0.0001843
S082	Polytherapy vs monotherapy	Hemispheric EPVS asymmetry index	0.7096384
S083	Polytherapy vs monotherapy	CSO-EPVS asymmetry index	0.7896417
S084	Polytherapy vs monotherapy	BG-EPVS asymmetry index	0.2597835
S085	Polytherapy vs monotherapy	Right BG relative EPVS burden	0.0087642
S086	Polytherapy vs monotherapy	Left BG relative EPVS burden	0.0107316
S087	Polytherapy vs monotherapy	Total BG relative EPVS burden	0.0057318
S088	Polytherapy vs monotherapy	Total CSO relative EPVS burden	0.0395826
S089	Polytherapy vs monotherapy	Right CSO-to-BG EPVS volume ratio	0.0252380
S090	Polytherapy vs monotherapy	Left CSO-to-BG EPVS volume ratio	0.0516390
S091	Epilepsy type	Total EPVS volume	0.271218
S092	Epilepsy type	Total BG-EPVS volume	0.148912
S093	EEG classification	Total EPVS volume	0.158764
S094	EEG classification	Total BG-EPVS volume	0.048327
S095	EEG lateralization	Total EPVS volume	0.044783
S096	EEG lateralization	Right hemispheric EPVS volume	0.018947
S097	EEG lateralization	Left hemispheric EPVS volume	0.028816
S098	EEG lateralization	Right CSO-EPVS volume	0.309684
S099	EEG lateralization	Left CSO-EPVS volume	0.279731
S100	EEG lateralization	Total CSO-EPVS volume	0.299642
S101	EEG lateralization	Right BG-EPVS volume	0.007386
S102	EEG lateralization	Left BG-EPVS volume	0.012437
S103	EEG lateralization	Total BG-EPVS volume	0.004817
S104	EEG lateralization	Hemispheric EPVS asymmetry index	0.409728
S105	EEG lateralization	CSO-EPVS asymmetry index	0.519684
S106	EEG lateralization	BG-EPVS asymmetry index	0.159731
S107	EEG lateralization	Right BG relative EPVS burden	0.013742
S108	EEG lateralization	Left BG relative EPVS burden	0.015684
S109	EEG lateralization	Total BG relative EPVS burden	0.009846

S110	EEG lateralization	Total CSO relative EPVS burden	0.009846
S111	EEG lateralization	Right CSO-to-BG EPVS volume ratio	0.079684
S112	EEG lateralization	Left CSO-to-BG EPVS volume ratio	0.089731
S113	EEG anatomical localization	Total EPVS volume	0.250684
S114	EEG anatomical localization	Total BG-EPVS volume	0.099217

Table Note: This table provides the full-precision computational inputs required to reproduce the Benjamini–Hochberg false discovery rate (FDR) adjustments applied to the exploratory clinical-association analyses. The primary clinical-association FDR family comprised 64 inferential tests corresponding to the predictor–outcome combinations reported in Supplementary Table S6. Entry of EPVS outcomes into this analysis depended on FDR-significant epilepsy–control differences in the preceding between-group analysis. The expanded sensitivity-analysis FDR family comprised 114 inferential tests corresponding to Supplementary Table S7; in this analysis, the preceding significance-based EPVS outcome-selection criterion was removed and the corresponding clinical associations were evaluated across an expanded 18-measure EPVS outcome set while the predictor-specific analytical architecture was otherwise retained. For both families, the FDR input consisted of the two-sided raw P values for the clinical-variable coefficients in continuous-predictor models, group effects in binary-predictor models, and omnibus effects in multicategorical-predictor models. Category-specific estimated marginal means (EMMs) did not constitute additional inferential tests. Post hoc pairwise EMM contrasts were not included in either Benjamini–Hochberg family; where applicable, these contrasts were adjusted separately using the Tukey procedure. Raw P values are provided at full computational precision and in the analytical order corresponding to Supplementary Tables S6 and S7. P and q values reported elsewhere in the manuscript and supplementary material were rounded for presentation. Application of the Benjamini–Hochberg procedure to the respective 64- and 114-value full-precision raw-P vectors reproduces the FDR-adjusted q values reported for the corresponding analysis families, subject to presentation rounding.

Abbreviations: ASM, antiseizure medication; BG, basal ganglia; CSO, centrum semiovale; EEG, electroencephalography; EMM, estimated marginal mean; EPVS, enlarged perivascular spaces; FDR, false discovery rate.
